# Combining Sulfonylureas with Anticancer Drugs: Evidence of Synergistic Efficacy with Doxorubicin In Vitro and In Vivo

**DOI:** 10.3390/ijms26041429

**Published:** 2025-02-08

**Authors:** Mateusz D. Tomczyk, Karolina Matczak, Marta Denel-Bobrowska, Grzegorz Dzido, Anna Kubicka, Daria Gendosz de Carrillo, Tomasz Cichoń, Marlena Golec, Beata Powieczko, Waldemar Rzetelny, Agnieszka B. Olejniczak, Horacio Pérez-Sánchez

**Affiliations:** 1 Department of Organic Chemistry, Bioorganic Chemistry and Biotechnology, Faculty of Chemistry, Silesian University of Technology, Krzywoustego 4, 44-100 Gliwice, Poland; bp313220@student.polsl.pl; 2 Department of Medical Biophysics, Faculty of Biology and Environmental Protection, University of Łódź, 141/143, 90-236 Łódź, Poland; karolina.matczak@biol.uni.lodz.pl (K.M.); anna.kubicka@edu.uni.lodz.pl (A.K.); 3 Screening Laboratory, Institute of Medical Biology, Polish Academy of Sciences, Lodowa 106, 93-232 Łódź, Poland; mdenel-bobrowska@cbm.pan.pl (M.D.-B.); aolejniczak@cbm.pan.pl (A.B.O.); 4 Department of Chemical Engineering and Process Design, Silesian University of Technology, Strzody 9, 44-100 Gliwice, Poland; grzegorz.dzido@polsl.pl; 5 Department of Physiology, Faculty of Medical Sciences, Medical University of Silesia, Medyków 18, 40-055 Katowice, Poland; dgendosz@sum.edu.pl; 6 Department of Histology and Cell Pathology, Faculty of Medical Sciences, Medical University of Silesia, Jordana 19, 41-808 Zabrze, Poland; 7 Center for Translational Research and Molecular Biology of Cancer, Maria Skłodowska-Curie National Research Institute of Oncology, Gliwice Branch, Wybrzeże Armii Krajowej Street 15, 44-102 Gliwice, Poland; tomasz.cichon@gliwice.nio.gov.pl; 8 Department of Radiopharmacy and Preclinical PET Imaging, Maria Skłodowska-Curie National Research Institute of Oncology, Gliwice Branch, Wybrzeże Armii Krajowej Street 15, 44-102 Gliwice, Poland; marlena.golec@gliwice.nio.gov.pl; 9 Department of Chemotherapy, Hospital of the Ministry of Interior and Administration in Łódź, Północna 42, 91-425 Łódź, Poland; wrzetelny@gmail.com; 10 Structural Bioinformatics and High Performance Computing Research Group (BIO-HPC), Computer Engineering Department, Universidad Católica de Murcia (UCAM), Campus de los Jerónimos 135, 30107 Murcia, Spain; hperez@ucam.edu

**Keywords:** synergistic cytotoxicity, combination therapy, combination index, drug repurposing, sulfonylureas, doxorubicin

## Abstract

Sulfonylureas (SUs)—a class of drugs primarily used to treat type 2 diabetes—have recently attracted interest for their potential anticancer properties. While some studies have explored the chemical modification or design of new SU derivatives, our work instead centers on biological evaluations of all commercially available SUs in combination with doxorubicin (DOXO). These antidiabetic agents act by stimulating insulin secretion via K_ATP_ channel inhibition, and because K_ATP_ channels share structural features with ATP-binding cassette (ABC) transporters involved in multidrug resistance (e.g., P-glycoprotein, MRP1, and MRP2), SUs may also reduce cancer cell drug efflux. In this study, we systematically examined each commercially available SU for potential synergy with DOXO in a panel of human cancer cell lines. Notably, combining DOXO with glimepiride (GLIM), the newest SU, results in a 4.4-fold increase in cytotoxicity against MCF-7 breast cancer cells relative to DOXO alone. Mechanistic studies suggest that the observed synergy may arise from increased intracellular accumulation of DOXO. Preliminary in vivo experiments support these findings, showing that DOXO (5 mg/kg, i.v.) plus GLIM (4 mg/kg, i.p.) is more effective at inhibiting 4T1 tumor growth in mice than DOXO alone. Additionally, we show that adding a small amount of the surfactant Tween-80 to culture media affects SU binding to bovine serum albumin (BSA), potentially unmasking anticancer effects of SUs that strongly bind to proteins. Overall, these results underscore the potential of repurposing existing SUs to enhance standard chemotherapy regimens.

## 1. Introduction

The main goal of our study was to investigate the potential anticancer effects of combining standard chemotherapeutic agents with sulfonylureas (SUs). SUs are a class of oral antidiabetic drugs commonly used to treat type 2 diabetes mellitus (T2DM). We found that several SUs, particularly glimepiride (GLIM), exhibit strong synergistic cytotoxic effects when combined with doxorubicin (DOXO) in various cancer cell lines. Our interest in SUs was initially inspired by the promising results observed with metformin, another antidiabetic drug known to inhibit tumor growth and enhance the efficacy of standard chemotherapy [1,2,3]. Although metformin and SUs differ considerably in their chemical structures and primary mechanisms of action, both are frequently prescribed to diabetic patients, including those undergoing cancer treatment. Thus, research on SUs is important both for their use in new cancer treatments and for understanding their effects on diabetic patients receiving chemotherapy.

Recent epidemiological studies suggest a correlation between diabetes and elevated cancer risk [4,5,6,7], which has increased interest in the anticancer properties of antidiabetic drugs such as metformin and SUs. As of early 2024, more than 170 clinical trials investigating the effects of metformin in cancer patients were registered on the NIH’s ClinicalTrials.gov website. In contrast, no comparable trials were registered for GLIM or other SUs, even though preclinical data suggest these drugs also may have anticancer activity. This discrepancy underscores the urgent need to investigate the potential benefits of SUs in cancer treatment—a gap that our study aims to address.

SUs primarily function by mimicking the role of ATP in pancreatic beta cells, acting as insulin secretagogues to effectively lower blood glucose in patients with T2DM [8]. They bind to sulfonylurea receptors (SURs) on the surface of beta cells. These receptors, together with Kir6.x-type subunits and other proteins, form ATP-dependent potassium (K_ATP_) channels. When SUs bind to SURs, they block the K_ATP_ channels, causing an intracellular accumulation of K^+^ ions, membrane depolarization, and the subsequent opening of voltage-gated calcium channels. The resulting increase in cytosolic Ca^2+^ levels stimulates insulin secretion and reduces hyperglycemia [9]. Beyond their glucose-lowering action, SUs are also reported to exhibit cytotoxic and anticancer effects.

Among SUs, glibenclamide (GLIB) has been studied most extensively for its anticancer properties. Preliminary data indicate that GLIB can directly inhibit the growth of various human cancer cells in vitro, such as melanoma, lung cancer, cervical cancer, hepatocellular carcinoma, bladder cancer, leukemia, breast cancer, prostate cancer, pancreatic cancer, and glioma [10,11,12,13,14,15,16]. One possible mechanism underlying its anticancer effect is the inhibition of K_ATP_ channels. Several studies have demonstrated that blocking K_ATP_ channels reduces cancer cell proliferation both in vitro and in vivo, especially in cells that overexpress these channels [17,18,19]. For example, Zhou et al. reported that inhibiting K_ATP_ channels with GLIB reduces proliferation in hepatocellular carcinoma cells [20]. Suzuki et al. found that GLIB enhances the sensitivity of melanoma cells to TRAIL-induced apoptosis by activating essential caspases and causing plasma membrane depolarization through the inhibition of K_ATP_ channels [21]. Pasello et al. later confirmed these synergistic apoptotic effects in malignant pleural mesothelioma cell lines [22].

Another possible mechanism for the anticancer activity of SUs is the inhibition of multidrug resistance proteins (MRPs). SURs belong to the ATP-binding cassette (ABC) transporter family, which also includes MRPs, such as P-glycoprotein (P-gp), MRP1, and MRP2. MRPs function as efflux pumps, actively removing drugs from cells and contributing to cancer multidrug resistance (MDR). Because SURs and MRPs have similar structures, SUs are also believed to inhibit MRPs and help overcome MDR. This inhibitory effect has been supported by several studies. For example, Golstein et al. demonstrated that GLIB and related sulfonylcyanoguanidines inhibit P-glycoprotein, leading to increased accumulation of colchicine in leukemia cells and enhanced its cytotoxic activity [23]. Similarly, Payen et al. showed that GLIB inhibits MRP1 in lung cancer cells, leading to increased intracellular accumulation of the MRP1 substrate vincristine. They also found that GLIB suppresses MRP2 activity in rat hepatocytes (which express only MRP2), resulting in higher levels of the MRP2 substrate carboxy-2′,7′-dichlorofluorescein [24]. Other studies have shown that GLIB can enhance the effectiveness of chemotherapeutic agents such as DOXO [11] and tamoxifen [25], both of which are substrates for different ABC transporters. These findings suggest that GLIB may act as a general inhibitor of ABC transporters, potentially improving cancer treatment efficacy by preventing drug efflux and increasing intracellular drug retention in cancer cells.

Evidence suggests that not only GLIB but also other SUs—such as GLIM [10,26,27,28,29,30,31] and glipizide (GLIP) [32,33,34,35,36]—can directly inhibit the growth of various human cancer cell lines and may also enhance the effectiveness of cancer therapies, potentially by inhibiting multidrug efflux pumps. However, the precise mechanisms underlying their synergy with chemotherapeutics remain unclear, and the anticancer potential of other SUs is still poorly understood. To help fill these gaps, we conducted the first comprehensive analysis of cytotoxic and synergistic effects of all commercially available SUs (glimepiride, gliquidone, glipizide, glibenclamide, carbutamide, chlorpropamide, tolazamide, tolbutamide) and one synthetic SU analog in combination with DOXO in a panel of human cell lines. The structures and names of all compounds tested are provided in the Supplementary Materials (Figure S1).

## 2. Results and Discussion

### 2.1. Effect of Tween-80 on Sulfonylurea Solubility

As noted by Hendriks et al., the interpretation of in vitro results on the anticancer activity of SUs is often complicated by several factors [37]. First, SUs tend to bind to proteins in the cell medium, which can result in lower free drug concentrations at the target site than reported. Second, the in vitro concentrations of SUs are often relatively high compared to the therapeutic concentrations achievable in patients’ serum. In addition, second-generation SUs can form colloidal aggregates in vitro, even at low micromolar ranges, due to their limited solubility. Shoichet’s team observed a significant reduction in the anticancer activity of many drugs when they were in colloidal form compared to their free form [38]. However, they also found that adding small amounts of non-ionic surfactants, such as Tween-80, to the cell medium can effectively disrupt the formation of large drug aggregates. This, in turn, can increase cell exposure to the drug and restore its activity.

Figure S2 in the Supplementary Materials presents literature data on the solubility of SUs and their binding affinity for human serum albumin (HSA) [39,40,41,42,43,44,45,46,47,48,49,50,51,52,53,54,55,56,57,58,59,60,61,62,63,64,65,66,67,68,69,70,71]. The data indicate that second-generation SUs have a higher binding affinity for HSA than first-generation SUs, but they are significantly less soluble. Given the similarity in size and shape between human and bovine serum albumins (BSA), it is likely that SUs will also bind to BSA in a similar manner. Indeed, Proks et al. demonstrated that high concentrations of BSA in the cell medium dramatically reduced GLIB’s ability to block K_ATP_ channels, while only moderately affecting the inhibitory effect of gliclazide (GLIC) [72]. This difference was attributed to the stronger binding of GLIB to BSA ($K_{d}$ = 0.67 × 10^−6^ M) compared to GLIC ($K_{d}$ = 1.55 × 10^−4^ M), resulting in a significant decrease in the concentration of free GLIB in the presence of BSA. This phenomenon could potentially cause false-negative results in in vitro studies, including our screening for synergistic effects.

**Figure 1:**
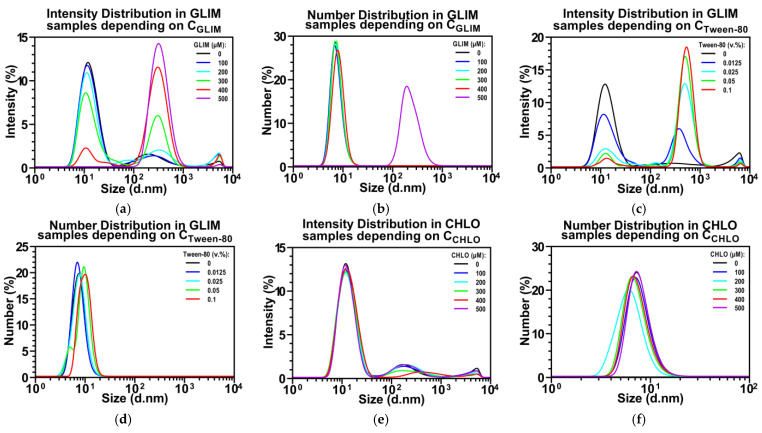
Effect of drug concentrations and Tween-80 on particle size distribution. Experiments were conducted in PBS (pH 7.4) at 37 °C, with 0.5% DMSO and 4.5 mg/mL BSA. This study examined 100–500 µM GLIM without Tween-80 (**a**,**b**); 300 µM GLIM with 0.0125–0.1% Tween-80 (**c**,**d**); and 100–500 µM CHLO with 0.05% Tween-80 (**e**,**f**). Size distribution histograms for GLIM samples in PBS alone and with 0.05% Tween-80, along with detailed DLS results, are provided in the Supplementary Materials (Figures S3–S5). Concentrations reported are expected concentrations (C_exp_), based on the total drug added. This preliminary experiment was conducted once (*n* = 1) to evaluate feasibility; additional replicates are needed to confirm findings and ensure reproducibility.

With this in mind, we investigated the behavior of selected SUs when mixed with 4.5 mg/mL BSA (corresponding to the protein level in our cell medium) and varying amounts of Tween-80. Figure 1 illustrates how the particle size distribution changes with different mixture compositions. Literature reports that at low concentrations (<10 mg/mL), BSA primarily exists as monomers with an intensity-weighted mean hydrodynamic diameter (${\bar{d}}_{I}$) of 10.5 nm [73], or a number-weighted mean hydrodynamic diameter (${\bar{d}}_{N}$) of 7.8 nm [74]. In our study, we found a ${\bar{d}}_{I}$ of 13.1 nm for BSA, with some larger agglomerates measuring 256.6 nm (Figure 1a). However, the number distribution showed a ${\bar{d}}_{N}$ of 7.38 nm, indicating the presence of BSA monomers (Figure 1b). The larger agglomerates did not appear in the number distribution, indicating that they represent a negligible percentage of the sample.

Figure 1a shows that as the concentration of GLIM increases, the intensity of the BSA particles gradually decreases, while their ${\bar{d}}_{I}$ increases from 13.01 nm to 16.59 nm. This increase in BSA size is likely due to protein unfolding upon interacting with multiple drug molecules. We observed the onset of BSA saturation with drug molecules at the expected GLIM concentration (C_exp_) of 200 µM. Beyond this concentration, the amount of free GLIM in the sample exceeds the critical aggregation concentration, leading to the formation of a distinct fraction of drug aggregates with a ${\bar{d}}_{I}$ of approx. 353 nm. As the GLIM concentration continues to rise, more drug aggregates are formed, eventually causing the BSA particles to disappear from the intensity distribution. A similar trend is observed in the number distribution (Figure 1b).

**Figure 2:**
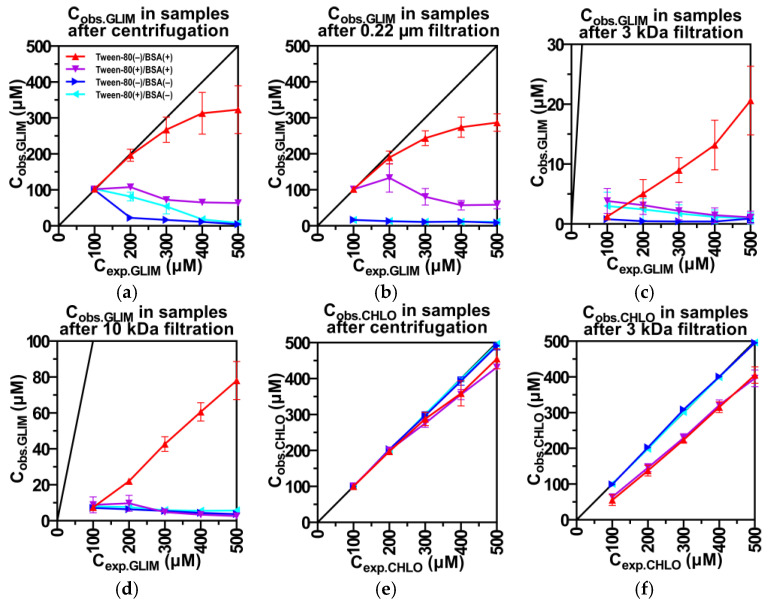
Impact of BSA and Tween-80 on observed drug concentrations (C_obs_) determined by HPLC using different separation techniques. C_obs_ of GLIM under varying conditions (**a**–**d**); C_obs_ of CHLO (**e**,**f**). In each panel, Tween-80(–) indicates the absence and Tween-80(+) the presence of the surfactant, while BSA(–) denotes the absence and BSA(+) the presence of the protein. The results are expressed as mean ± SD (*n* = 3).

Figure 2a shows that after centrifugation of samples containing only GLIM (blue ▶), the observed concentration (C_obs_) of the drug matches the expected concentration up to 100 µM. However, when this threshold is exceeded, C_obs_ drops to approx. 5 µM. Since the size of the drug aggregates remains relatively constant between 100 and 500 µM (Figure S3), the decrease in C_obs_ is likely due to the sedimentation of large clumps of precipitated drug that may be overlooked by DLS or considered artifacts. Using syringe filters with a 0.22 µm pore size to remove these aggregates stabilizes C_obs_ within the 8.6–16.6 µM range across all tested samples (Figure 2b).

In the presence of BSA (red ▲), solutions containing high concentrations of GLIM that were previously turbid become clear, and C_obs_ increases significantly (Figure 2a). We attribute these changes to the formation of drug–protein complexes that reduce the amount of free GLIM and limit its aggregation. These small-sized complexes remain suspended in the supernatant, which is then used for HPLC analysis, resulting in a higher observed C_obs_. When we used 0.22 µm syringe filters, the resulting C_obs_ values were similar to those obtained from centrifuged samples (Figure 2b), probably due to minimal removal of BSA by the filters. HPLC results indicate that about 95% of the BSA particles pass through the 0.22 µm filter together with bound drug molecules (Figure S6). At a C_exp_ of 200 µM, BSA begins to saturate with drug molecules, leading to a gradual deviation between C_exp_ and C_obs_ (Figure 1a).

Increasing the concentration of Tween-80 results in a higher intensity of the drug particles (Figure 1c) and a larger diameter of the BSA fraction (Figure 1d). The presence of Tween-80 in samples containing BSA (purple ▼) significantly reduces C_obs_ values at C_exp_ above 100 µM, regardless of the filtration method used. These observations suggest that Tween-80 reduces the binding of GLIM molecules to BSA, causing the unbound drug molecules to rapidly form large aggregates. This aligns with literature reports indicating that surfactants can induce partial protein unfolding and disrupt drug–protein interactions [75,76,77]. Such effects should be carefully considered when interpreting in vitro results, as they may impact the observed activity of tightly binding drugs.

Ultrafiltration using a 3 kDa molecular weight cut-off (MWCO) membrane significantly reduces the C_obs_ of GLIM in samples containing BSA (Figure 2c). This is because the 3 kDa membrane removes approx. 97% of BSA particles, including those bound to drug molecules and large drug aggregates (Figure S6). Interestingly, ultrafiltration with a 10 kDa membrane (Figure 2d) also effectively removes BSA particles (Figure S6), but results in much higher C_obs_ of GLIM in BSA-containing samples. Given the consistently low C_obs_ levels in BSA-free samples filtered with both membranes, we suggest that the higher C_obs_ observed with the 10 kDa membrane in BSA-containing samples are due to larger drug–protein complexes passing through the membrane, carrying more drug molecules. The addition of Tween-80 to BSA-containing samples dramatically reduces the C_obs_ of GLIM without significantly altering the amount of BSA in the filtrate (Figure S6). This reduction in C_obs_ is not seen in BSA-free samples; instead, adding Tween-80 (cyan ◀) slightly increases C_obs_ after ultrafiltration, likely because Tween-80 partially reduces the size of aggregates.

For comparison, we performed DLS analysis on chlorpropamide (CHLO), a first-generation SU with better solubility (1.0 mM vs. 12.2 µM) but lower binding affinity to HSA compared to GLIM (${nK}_{a}$ = 0.62 × 10^5^ vs. 9.1 × 10^5^ M^−1^). Increasing the concentration of CHLO did not affect the intensity of the BSA particles or result in the formation of drug aggregates, as indicated by size distribution histograms (Figure 1e,f). The addition of Tween-80 also did not affect the concentration of free CHLO in the presence of BSA (Figure 2e,f). Furthermore, no differences between C_exp_ and C_obs_ were observed in BSA-free samples, regardless of the filtration method or the presence of surfactant (Figure 2e,f). These results suggest that Tween-80 has a less competitive effect on CHLO binding than on second-generation GLIM and that unbound CHLO molecules do not form aggregates, even at relatively high concentrations.

In summary, at high drug concentrations, multiple GLIM molecules can bind to BSA, reducing aggregate formation and increasing C_obs_ in samples filtered by methods that allow small drug–protein complexes to pass through. In the absence of BSA, GLIM readily forms large aggregates with diameters over 300 nm. The addition of Tween-80 significantly reduces the size of these aggregates in both the absence and presence of BSA, potentially enhancing drug uptake by cells. However, Tween-80 may also reduce the number of drug molecules bound to BSA, increasing the concentration of free drug molecules and potentially promoting the formation of aggregates not observed in surfactant-free samples. Since the effects of these phenomena on the biological activity of SUs is currently unknown, our next step is to investigate how Tween-80 affects cell viability and the activity of co-administered drugs.

### 2.2. Effect of Tween-80 on Drug-Induced Cytotoxicity

Recent studies suggest that the increased cytotoxicity of poorly soluble drugs in the presence of surfactants may result from enhanced drug solubility, disruption of large drug aggregates, or increased drug uptake by cells [78,79,80,81]. Additionally, some surfactants may increase the sensitivity of cancer cells to cytostatic drugs. For example, Tween-80 has been shown to increase the uptake of epirubicin, a structural analog of doxorubicin, by enhancing cell permeability and reducing its excretion by inhibiting P-gp activity [82].

**Figure 3:**
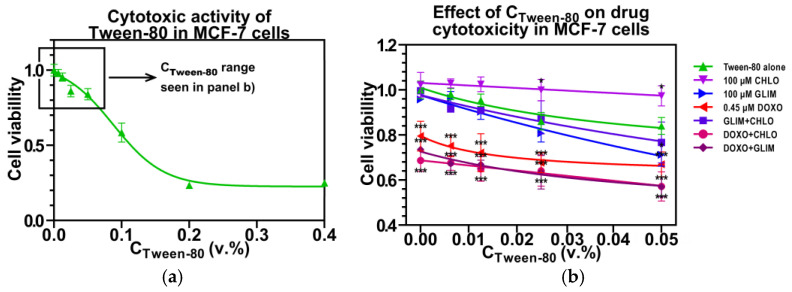
Effect of Tween-80 on the viability of MCF-7 breast cancer cells (**a**) and on the cytotoxicity of co-administered drugs (**b**). Cells were exposed to the compounds for 72 h. Results are presented as means ± SD (*n* = 3). *, *p* < 0.05 vs. Tween-80 alone; ***, *p* < 0.001 vs. Tween-80 alone.

Before examining how Tween-80 affects the cytotoxicity of co-administered drugs, we first evaluated its own impact on cancer cell survival, as high levels of surfactants can also exhibit cytotoxic effects. As shown in Figure 3a, Tween-80 significantly reduced the survival of MCF-7 cells at concentrations above 0.05% (*v*/*v*). Therefore, we limited our cell studies to this concentration range.

Figure 3b shows that co-administration of Tween-80 increases the cytotoxicity of second-generation GLIM in a dose-dependent manner, while having no significant effect on first-generation CHLO. The 24.6% decrease in cell viability observed with GLIM in the presence of 0.05% Tween-80 may be due to the disruption of its binding to BSA and the breakdown of large drug aggregates. In contrast, the lack of a similar effect on CHLO could be explained by its higher solubility and the minimal impact of Tween-80 on its interaction with BSA. Interestingly, CHLO significantly reduced the cytotoxicity of Tween-80 alone but did not affect the cytotoxicity of the GLIM + CHLO combination, which remained comparable to that of GLIM alone. This suggests that the cytoprotective effects of CHLO are primarily mediated through cellular mechanisms rather than interactions with surfactant molecules in the culture medium.

Figure 3b demonstrates that Tween-80 also enhances the cytotoxicity of DOXO in a dose-dependent manner, reducing MCF-7 cell viability by an additional 12.6% at a concentration of 0.05% Tween-80. Similar effects were observed for the DOXO + GLIM and DOXO + CHLO combinations, although these effects were less pronounced compared to GLIM alone. The increased cytotoxicity of drug combinations with Tween-80 can be partly attributed to the surfactant’s ability to prevent drug binding to BSA, which would otherwise diminish their activity (as observed with GLIM), and its potential influence on DOXO metabolism in cancer cells. Notably, both DOXO + GLIM and DOXO + CHLO exhibited greater cytotoxicity than their respective single agents, regardless of the presence of Tween-80. This suggests that the increased cytotoxicity is likely due to a synergistic interaction between the drugs themselves rather than the influence of the surfactant.

Although the exact mechanisms of the surfactant’s effects remain unclear and warrant further investigation, we believe that incorporating Tween-80 can improve the assessment of cytotoxicity and the potential synergistic effects of SUs by minimizing the likelihood of false-negative results in screening studies. Encouraged by these observations, we have decided to maintain 0.05% Tween-80 in the culture media for subsequent experiments.

### 2.3. Preliminary Screening for Cytotoxic Synergy

Before studying the synergistic cytotoxic effects, we first evaluated the stability of SUs and their mixtures. We monitored their concentrations in equimolar mixtures with DOXO by incubating them in PBS (pH 7.4) at 37 °C for 7 to 14 days. Most SUs demonstrated high stability under these conditions, and no new adducts were detected in any of the samples. The only exceptions were gliclazide (GLIC) and tolazamide (TOLA), which exhibited slow but noticeable degradation. This finding is consistent with literature data suggesting that these two SUs may degrade over time [83,84]. However, the degradation rates were low enough that they are unlikely to significantly impact the in vitro studies. Detailed results and conclusions from the stability studies can be found in the Supplementary Materials (Figure S7).

Existing literature provides limited data on the cytotoxicity of SUs, focusing mainly on their impact on breast cancer cells. Alkhalil et al. showed that GLIM, GLIB, and GLIP exhibited relatively low cytotoxicity against MCF-7 cells, with IC_50_ values ranging from 216.3 to 325.2 µM [10]. Another study reported an IC_50_ = 50 µM for GLIM against MCF-7 cells [13]. Based on the results of Faridi et al., we calculated an IC_50_ = 79.8 µM for GLIM against MCF-7 cells [26]. Overall, SUs can be considered safe drugs with low toxicity, as reflected in their minimal in vitro cytotoxicity.

We first determined the IC_50_ values for all tested compounds after 72 h of incubation with MCF-7 cells. Detailed results and the response curves used to calculate the IC_50_ values are provided in the Supplementary Materials (Figure S8). The IC_50_ values obtained are summarized in Table 1. The IC_50_ value for DOXO treatment alone was determined to be 0.46 µM. Most SUs did not achieve IC_50_ values below 500 µM or achieved values close to this level. The highest cytotoxicity was observed with second-generation SUs, particularly GLIM and gliquidone (GLIQ).

We then examined the response of MCF-7 cells to combinations of DOXO and SUs using the Chou–Talalay method [85]. This method calculates the combination index (CI) for two drugs as follows:

(1) $$\mathrm{CI}=\frac{({D)}_{1}}{{\left(D_{x}\right)}_{1}}+\frac{({D)}_{2}}{{\left(D_{x}\right)}_{2}}$$

where (D_x_)_1_ and (D_x_)_2_ are the doses of each drug required alone to achieve an x% effect, while (D)_1_ and (D)_2_ are the doses of the drugs when used together to achieve the same effect.

**Figure 4:**
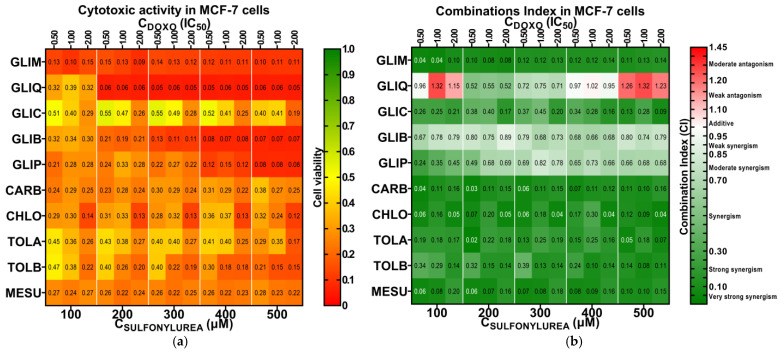
Heat maps illustrate the effects of combined treatment on the survival of MCF-7 cells (**a**) and the synergistic effects observed for these treatments (**b**). In the CI heat map, green indicates very strong synergy (CI < 0.1), strong synergy (0.1 ≤ CI < 0.3), and synergy (0.3 ≤ CI < 0.9), white indicates additive effects (0.9 ≤ CI < 1.1) and red indicates weak antagonism (1.1 ≤ CI < 1.2), moderate antagonism (1.2 ≤ CI < 1.45), and antagonism (CI > 1.45). Detailed information, including median effect plots and CI values, can be found in the Supplementary Materials (Figures S9–S12).

Figure 4a shows the fraction of viable cells relative to untreated controls, while Figure 4b illustrates the synergistic effects based on the Chou–Talalay method. A comparison of these two figures reveals that combination treatments generally result in increased cytotoxicity compared to single treatments (i.e., treatment involving only one drug), often accompanied by significant synergistic effects. Looking at the survival heat map (Figure 4a) from left to right, we observe that increasing the concentration of both drugs typically leads to a dose-dependent decrease in MCF-7 cell survival. In general, combinations of DOXO with second-generation SUs exhibited higher cytotoxicity than those with first-generation SUs, consistent with the differences in cytotoxicity observed between the SU generations (Table 1). The most pronounced cytotoxic effects in MCF-7 cells were observed with the DOXO + GLIM and DOXO + GLIQ combinations, which reduced cell viability to approx. 11% and 5%, respectively.

When analyzing the synergy heat map (Figure 4b) from top to bottom, we notice that first-generation SUs show very strong synergy with DOXO, as reflected by consistently low average combination index ($\bar{CI}$) values. The strongest synergies were observed for DOXO + CARB ($\bar{CI}$ = 0.10), DOXO + CHLO ($\bar{CI}$ = 0.11), and DOXO + MESU ($\bar{CI}$ = 0.11), followed by DOXO + TOLA ($\bar{CI}$ = 0.16) and DOXO + TOLB ($\bar{CI}$ = 0.19). In contrast, second-generation SUs generally showed moderate synergy with DOXO, with some combinations even displaying antagonistic effects. The exception was the DOXO + GLIM combination, which showed strong synergy ($\bar{CI}$ = 0.11). Other second-generation combinations, such as DOXO + GLIC ($\bar{CI}$ = 0.25), DOXO + GLIP ($\bar{CI}$ = 0.65), DOXO + GLIB ($\bar{CI}$ = 0.75), and DOXO + GLIQ ($\bar{CI}$ = 0.93), exhibited weaker synergy. The strong synergy observed with first-generation SUs can likely be attributed to their ability to significantly enhance the cytotoxicity of DOXO relative to their own minimal activity, resulting in very low CI values. In contrast, second-generation SUs, which are more cytotoxic on their own, yield higher CI values when combined with DOXO, thus displaying weaker synergy.

We selected GLIM, GLIQ, and CHLO for further studies due to their diverse effects. GLIM alone exhibited low cytotoxicity in MCF-7 cells (IC_50_ = 186.2 μM), but its combination with DOXO resulted in strong synergy ($\bar{CI}$ = 0.11) and significant cytotoxicity. In contrast, the DOXO + GLIQ combination effectively reduced MCF-7 cell viability but primarily showed additive to moderately antagonistic effects ($\bar{CI}$ = 0.93). The DOXO + CHLO combination also showed strong synergy ($\bar{CI}$ = 0.11), despite CHLO being cytotoxically inactive in MCF-7 cells (IC_50_ > 500 μM).

### 2.4. Expanded Screening of Selected Drug Combinations

**Figure 5:**
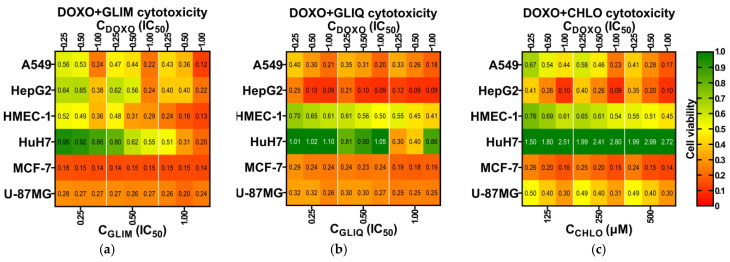

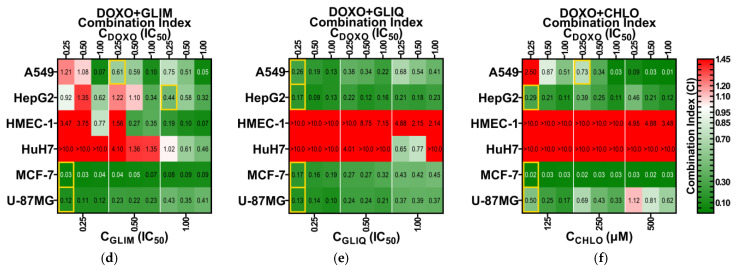
Heat maps illustrate selected drug combinations’ effects on cell panel viability (**a**–**c**) and the synergistic effects observed for these treatments (**d**–**f**). For CHLO, which did not achieve an IC_50_ below 500 µM, three successive drug dilutions were applied. In the CI heat maps, green indicates very strong synergism (CI < 0.1), strong synergism (CI < 0.3), and synergism (CI < 0.9). White indicates additive effects (0.9 < CI < 1.1), while red indicates weak antagonism (1.1 < CI < 1.2), moderate antagonism (1.2 < CI < 1.45), antagonism (1.45 < CI < 3.3), strong antagonism (3.3 < CI < 10), and very strong antagonism (CI > 10). Cells marked in yellow indicate dose combinations selected for the cell cycle studies discussed in the next section. Additional information on cytotoxicity and CI values can be found in the Supplementary Materials (Figures S13–S15).

In the second phase of our cytotoxicity synergy assessment, we used the same approach as in the initial study but administered lower doses of the drugs, corresponding to fractions of their IC_50_ values. The results of MTT assays on a panel of cell lines (Figure 5a–c) showed a predominant dose-dependent decrease in cell viability with increasing concentrations of both drugs. Combined treatment typically resulted in a synergistic increase in cytotoxicity compared to DOXO monotherapy (Figure S13), as indicated by low CI values on synergy heat maps (Figure 5d–f). Based on these values, we identified cells (highlighted in yellow) that represent the most effective combination treatments. These combinations correspond to the lowest doses of drugs that show synergistic cytotoxicity in their respective cell lines. Among these, the MCF-7 cell line showed exceptional sensitivity to the DOXO + GLIM combination. Specifically, administration of ¼ IC_50_ DOXO (0.12 µM) and ¼ IC_50_ GLIM (45.1 µM) resulted in a 4.4-fold improvement in cytotoxicity, reducing MCF-7 cell viability to 16.0% compared to 70.2% for DOXO alone (Figure S13).

When analyzing the heat maps from top to bottom, we observe diverse responses of cell lines to different drug combinations and doses. In some cases, these combinations proved to be ineffective or even antagonistic. Such opposing effects were particularly evident in HuH7 cells, where combination treatments led to a significant increase in proliferation compared to untreated controls. Cytoprotective effects of SUs were also observed in non-cancerous HMEC-1 cells, as a partial reduction in DOXO cytotoxicity resulted in a smaller decrease in proliferation compared to DOXO monotherapy. These varied cellular responses to identical combination treatments underscore the complexity and challenges in developing effective combination therapies that incorporate SUs.

### 2.5. Effect of Combined Treatment on Cell Cycle Progression

**Figure 6:**
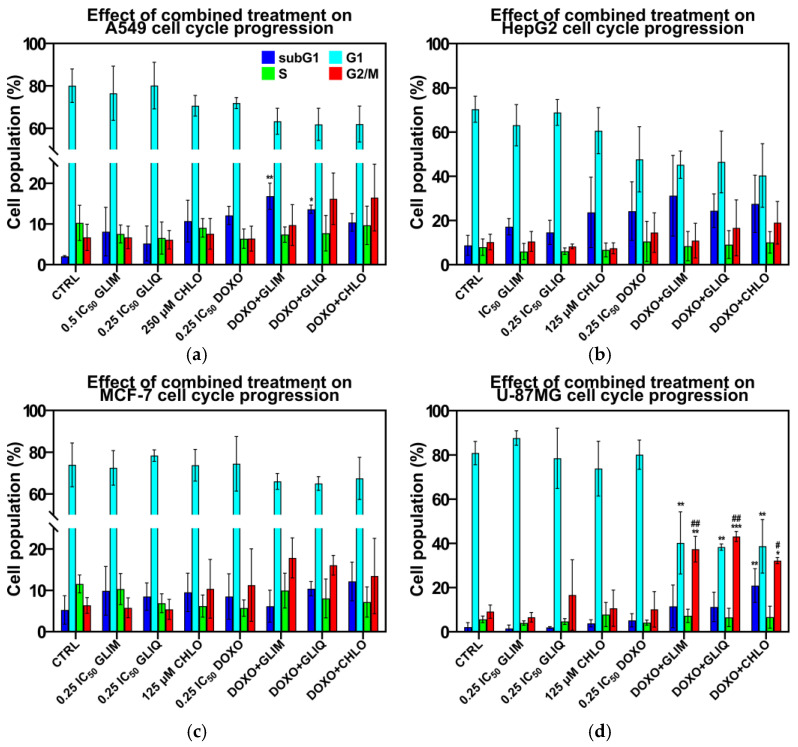
Cell cycle phase distribution analysis in A549 (**a**), HepG2 (**b**), MCF-7 (**c**), and U-87MG (**d**) cells following treatment with single drugs and combinations for 72 h, assessed by propidium iodide staining and flow cytometry. Results are expressed as the mean ± SD (n = 3). *, *p* < 0.05 vs. CTRL; **, *p* < 0.01 vs. CTRL; ***, *p* < 0.001 vs. CTRL; #, *p* < 0.05 vs. 0.25 IC_50_ DOXO; ##, *p* < 0.01 vs. 0.25 IC_50_ DOXO. Detailed data can be found in the Supplementary Materials (Figure S16).

Combined treatments with DOXO and SUs can inhibit cell growth by affecting specific cellular processes. To better understand these effects, we performed flow cytometry studies on the most effective combinations previously identified (highlighted in yellow in Figure 5d–f). Figure 6a–d show the cell cycle distribution after 72 h of treatment with these regimens.

In general, the combined treatments had a more pronounced effect on cell cycle arrest, particularly in the subG1 and G2/M phases, compared to single treatments with either DOXO or SUs. In the U-87MG cell line, the combined treatments significantly increased the population of cells in the G2/M phase, from 9.1% in the control to 32.2–43.1%, and the population in the subG1 phase, from 2.1% in the control to 11.5–20.9%. Similar effects were observed in the MCF-7 and A549 cell lines, where combined treatments induced a strong G2/M arrest and an even more pronounced increase in the subG1 phase population. However, the increase in the subG1 phase was less pronounced than the sum of the effects of single treatments. In HepG2 cells, combined treatments resulted in a less pronounced G2/M arrest compared to other cell lines, but caused a significant increase in the subG1 phase population, from 8.8% in control to 24.4–31.2%.

Cell cycle arrest in the G2/M phase may indicate an apoptotic mode of cell death, while the consistency of these effects across different cell lines suggests a potential common mechanism behind the synergy of DOXO and SUs. However, this mechanism remains uncertain and requires further investigation. The effects observed for SUs in our study are similar to those reported for structurally related diarylsulfonylureas, which have demonstrated antiproliferative effects by inhibiting tubulin polymerization and causing cell cycle arrest at the G2/M phase [86].

### 2.6. Effect of Combined Treatment on Doxorubicin Accumulation

Previous studies indicate that GLIB may act as a general inhibitor of ABC transporters, potentially leading to increased accumulation of anticancer drugs [23,24]. Payen et al. observed that GLIB induced a dose-dependent increase in the intracellular accumulation of calcein, an MRP1 substrate, at concentrations as low as 3.12 µM [24]. However, this effect was not observed with all SUs. For example, TOLB did not increase calcein accumulation, even at concentrations as high as 500 µM. This suggests that the inhibitory effect of SUs may depend on specific structural features of each drug, rather than being a general trait of all SUs. To determine if the differences in synergistic effects observed in our studies were related to increased DOXO accumulation, we tested the effect of various concentrations of GLIM, GLIQ, and CHLO on DOXO accumulation in whole-cell assays.

**Figure 7:**
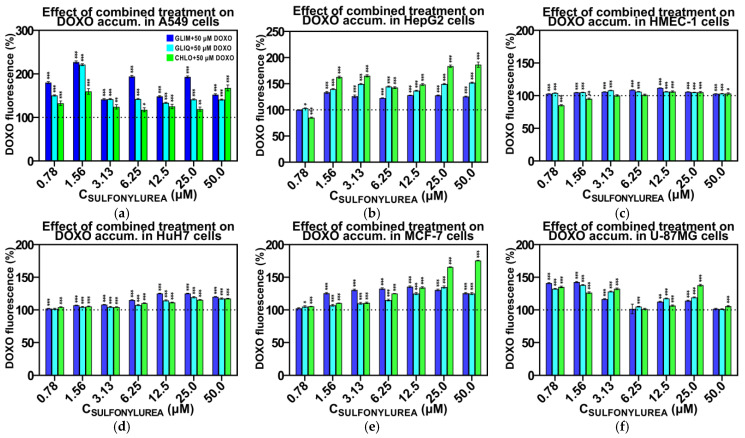
Effects of selected SUs on intracellular DOXO accumulation in A549 (**a**), HepG2 (**b**), HMEC-1 (**c**), HUH7 (**d**), MCF-7 (**e**), and U-87MG cells (**f**). Cells were treated with 50 µM DOXO and various concentrations of SUs (from 0.78 to 50 µM) for 3 h. Intracellular DOXO levels were measured by fluorometry and are expressed as a percentage relative to the control (DOXO alone). The bars represent the mean ± SD (n = 3). *, *p* < 0.05 vs. control; **, *p* < 0.01 vs. control; ***, *p* < 0.001 vs. control.

Figure 7a–f show that the co-administration of SUs significantly increased DOXO levels in the synergistic cell lines A549, HepG2, MCF-7, and U-87MG. However, no similar effect was observed in the antagonistic cell lines HMEC-1 and HuH7. Interestingly, the effect of DOXO accumulation in sensitive cell lines was consistent for different SUs and started at relatively low concentrations of 0.78–1.56 µM, similar to those found in patients’ blood samples (Figure S2). Higher doses of SUs did not further increase DOXO accumulation. On average, DOXO accumulation in the sensitive cell lines increased by 120–150%. In some cases, the effects were even more pronounced—for example, co-administration of 50 µM DOXO and 1.56 µM GLIM in A549 cells resulted in a 227% increase in DOXO level compared to DOXO monotherapy. Although we did not observe a clear dose–response relationship, our results suggest a close correlation between increased DOXO levels and the synergistic effects observed in the MTT assay.

### 2.7. Ex Vivo Toxicity and Microscopy

**Figure 8:**
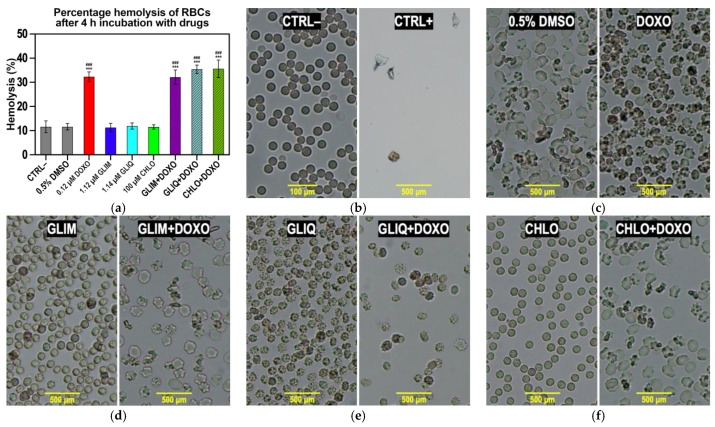
Percentage of hemolysis caused by SUs, naïve DOXO, and their combinations after 4 h of incubation at RT under open-air conditions (**a**). Microscopic images showing the impact of the drugs and their combinations on RBCs after 15 min of incubation at RT in open-air conditions (**b**–**f**). The negative control (CTRL−) is untreated blood in PBS, while the positive control (CTRL+) is blood incubated in deionized water. Each experimental sample contains 0.5% DMSO, except for the control samples. The bars represent the mean ± SD (*n* = 3). ***, *p* < 0.0001 vs. CTRL− ###, *p* < 0.0001 vs. 0.5% DMSO.

Ex vivo erythrocyte membrane toxicity studies serve as a bridge between in vitro and in vivo research, providing a more physiologically relevant assessment of a compound’s effects and identifying potential toxic interactions before in vivo trials. Figure 8a shows hemolysis results using Drabkin’s method [87]. The negative control (CTRL−), consisting of RBCs incubated for 4 h in PBS, exhibited 11.6% hemolysis with preserved erythrocyte shape. The positive control (CTRL+), consisting of RBCs incubated in demineralized water, showed complete cell lysis, which was considered as 100% hemolysis.

The observed morphological changes in erythrocytes provide valuable insights into the effects of the tested substances on cellular structures (Figure 8b–f). In CTRL−, erythrocytes maintained their characteristic biconcave shape, indicating membrane stability under experimental conditions, consistent with previous studies on erythrocyte morphology in isotonic environments [88]. In contrast, CTRL+ exhibited complete hemolysis and total membrane disruption, confirming the method’s capability to evaluate extreme toxic effects.

Slight erythrocyte shrinkage was observed in the 0.5% DMSO sample (Figure 8c), but the level of hemolysis by DMSO (12.2%) remained comparable to CTRL− (11.6%). This is consistent with previous studies suggesting that DMSO has minimal effect on hemolysis under controlled conditions while potentially altering osmotic balance [89]. Reported hemolytic concentrations of DMSO vary widely due to differences in experimental conditions. For example, Yi et al. observed hemolysis at 0.2% DMSO [90], whereas other studies found no significant hemolysis even at 25% DMSO in 0.6% saline [91]. Such discrepancies may be due to differences in the extracellular environment; in our case, PBS (containing 0.9% NaCl) may mitigate hemolytic effects of 0.5% DMSO. In contrast, naïve DOXO caused significant erythrocyte rupture and morphological changes (Figure 8c), consistent with its known high toxicity. These effects, likely due to disruption of membrane and cytoskeletal structures, have been extensively documented [92]. The observed hemolysis rate of 32.3% is also consistent with literature data [93,94].

Hemolysis is a known side effect of SUs. The most common cause is extravascular hemolysis associated with G6PD deficiency (e.g., for GLIB) [95] or immune complex-mediated mechanisms (e.g., for CHLO, GLIB, and TOLB) [96,97]. In addition, there are reports of in vitro photohemolytic properties induced by exposure to sunlight (e.g., for CHLO, GLIP, GLIQ, and TOLB) [98]. In our study, we incubated RBCs with GLIM, GLIQ, and CHLO at clinically relevant concentrations (Figure S2) for 4 h at RT in the dark. There was no substantial increase in hemolysis (11.2–11.9%) compared to the negative control (Figure 8a), except for changes in cell morphology with GLIQ (Figure 8e), which remain unexplained. When SUs were co-administered with DOXO, the levels of hemolysis (32.1–35.6%) and cell shape changes were similar to those observed with naïve DOXO (32.3%), indicating no synergistic effect of SUs on erythrocyte toxicity

### 2.8. Findings from In Vivo Studies

Evidence that SUs can enhance the anticancer activity of other cytostatics in vivo remains limited, and available studies lack systematic comparisons among different SUs. To date, only two such studies have been published. Cocca et al. were the first to examine the anticancer effect of GLIB (0.06 mg/kg/day, orally), alone or combined with tamoxifen (1 mg/kg/day, s.c.), on mammary tumors in non-diabetic and diabetic rats [25]. After 20 days of treatment, in non-diabetic rats, 64% of tumors responded to GLIB (they either regressed or remained stable), 57% of tumors responded to tamoxifen, and all tumors responded to the combined treatment (58% regressed and 42% remained stable). In diabetic rats, no response to GLIB alone was observed, while 75% of tumors responded to tamoxifen, and 89% of tumors responded to the combined treatment (68% regressed and 21% remained stable). The reason for the lack of GLIB response in diabetic rats is unclear, but it may be linked to distinct histological tumor features and differences in K^+^ channel expression between diabetic and non-diabetic animals.

A recent study by Zhan et al. investigated how combining GLIM (5 mg/kg every 2 days, i.p.) with anti-PD1 therapy affects colon and melanoma tumors in mice [30]. In the CT26 colon cancer model, GLIM alone increased CD8^+^ T cell infiltration by about 100% and reduced tumor mass by 45%, while anti-PD1 alone reduced tumor mass by about 56%. However, when GLIM and anti-PD1 were combined, the treatment unexpectedly resulted in only a 2% decrease in tumor mass. In contrast, in the MC38 melanoma model, GLIM alone had little effect on CD8^+^ T cell numbers (<10% increase) but still reduced tumor mass by 57%, which was greater than the 33% reduction achieved by anti-PD1 alone. The combination therapy in this model reduced tumor mass by 34% and increased CD8^+^ T cells to levels similar to anti-PD1 therapy alone. These results suggest that combining GLIM with anti-PD1 or other chemotherapies may produce different outcomes depending on the tumor type.

Notably, sulfonylureas can exhibit differential effects not only across various cancer types but also within the same cancer model. For example, Qi et al. demonstrated that GLIP, but not GLIM, inhibited tumor growth and metastasis in 4T1 breast cancer and B16F10 melanoma xenografts, as well as in transgenic MMTV-PyMT mouse models of breast cancer [32].

**Figure 9:**
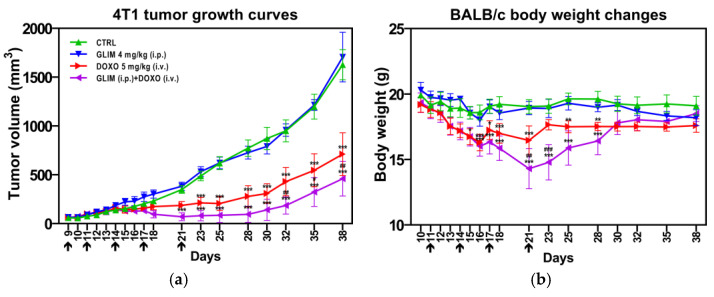
Therapeutic efficacy of DOXO alone and in combination with GLIM against 4T1 breast cancer in vivo. Tumor growth curves (**a**) and body weight changes (**b**) in BALB/c mice bearing 4T1 tumors. Beginning nine days after tumor inoculation, mice with well-developed tumors were treated with either monotherapies or combination therapy. GLIM was administered intraperitoneally (i.p.) at 4 mg/kg, and DOXO was administered intravenously (i.v.) at 5 mg/kg. Both treatments were given simultaneously on days 9, 11, 14, 17, and 21, as indicated by the arrows in the figure. Tumor volume and body weight were measured daily for the first 8 days and then three times per week. Data are presented as mean ± SD (*n* = 5–6). *, *p* < 0.05 vs. CTRL; **, *p* < 0.01 vs. CTRL; ***, *p* < 0.001 vs. CTRL; #, *p* < 0.05 vs. DOXO; ##, *p* < 0.01 vs. DOXO 5 mg/kg (i.v.); ###, *p* < 0.001 vs. DOXO 5 mg/kg (i.v.).

In our in vivo study, we observed a significant inhibition of tumor growth in the group treated with the DOXO + GLIM combination compared to those receiving monotherapies (Figure 9a). By the end of this study, tumor volume in the combination group was 35% smaller than in the DOXO-only group and 72% smaller than in the control group. In contrast, mice treated with GLIM alone showed no reduction in tumor size compared to untreated controls, consistent with previous findings by Qi et al. [32], who also observed no effect of GLIM on 4T1 tumors in BALB/c mice. During the treatment phase, we noted substantial weight loss in the combination therapy group; however, the mice fully regained their initial weight after the cessation of compound administration (Figure 9b). We also found that mice treated with GLIM alone showed no significant change in body weight compared to controls, in agreement with other mouse and rat studies [99,100].

## 3. Materials and Methods

### 3.1. HPLC Analysis

The drug content in the samples was determined using a previously described HPLC method [101]. The analysis was performed on an Ultimate 3000 HPLC system (Dionex Co., Germering, Germany), equipped with a UV–Vis detector and a TSKgel ODS-100V column (particle size: 5 µm; dimensions: 4.6 mm i.d. × 15 cm; Tosoh Co., Tokyo, Japan). The mobile phase consisted of MeCN and 20 mM KH_2_PO_4_ buffer (pH 3.0) in an appropriate ratio. The flow rate was maintained at 1.0 mL/min, with the column temperature set to 30 °C. All compounds were detected at 228 nm.

Detailed information, including retention time (*t*_R_), the linear equation, and the correlation coefficient (R^2^) for each drug, along with the specific mobile phase compositions, is provided in the Supplementary Materials (Figure S17). Standard curves were generated by linear regression analysis of peak area versus analyte concentration within a range of 15.63–1000 µM. Stock solutions were prepared by dissolving solid drugs in DMSO to a concentration of 100 mM, and standard curve samples were prepared through serial half-dilutions in DMSO. Additional details on the analysis of DOXO are provided in Figure S18, and information on the analysis of BSA can be found in Figure S19.

### 3.2. DLS Measurements

Dynamic light scattering (DLS) measurements were performed using a Zetasizer Nano ZS instrument (Malvern Instruments, Malvern, UK). Samples of 2 mL were placed in disposable PMMA cuvettes (cat. #634-0677; VWR, Gdańsk, Poland) and measured at 37 °C. To ensure accuracy, each sample was measured at least three times. The data were processed using DTS v6.2 software (Malvern Instruments).

### 3.3. Analysis of Drug Solubility

Drug solubility was analyzed by preparing samples where 1–5 µL of a 100 mM drug stock in DMSO was added to 995 µL of PBS (pH 7.4). Some samples included 0.05% (*v*/*v*) Tween-80. For experiments involving BSA, a separate solution was prepared by dissolving 4.5 mg/mL of BSA (cat. #422381B, VWR) in the same PBS (pH 7.4), with or without 0.05% (*v*/*v*) Tween-80. All samples were incubated at 37 °C with shaking for 24 h. After incubation, the samples were either centrifuged at 13,500 rpm (RCF = 12,225 g) for 15 min using a Micro Star 12 microcentrifuge (VWR), and 150 µL of the supernatant was collected for HPLC analysis, or filtered using 0.22 µm PVDF syringe filters (13 mm i.d., cat. #514-1251, VWR) or AcroPrep™ Advance 96-well filter plates (Pall Corporation, Dreieich, Germany) with various molecular weight cut-offs.

### 3.4. Analysis of Drug Stability

To evaluate drug interactions and stability, 1.5 mL samples were prepared with either a combination of SUs and DOXO, each at a concentration of 100 µM, or with each drug individually at the same concentration. The samples were prepared in PBS (pH 7.4) containing 0.05% (*v*/*v*) Tween-80 and 0.5% (*v*/*v*) DMSO. Additionally, the stability of DOXO was analyzed under different pH conditions. The samples were placed into 2 mL HPLC vials, sealed, and incubated at 37 °C for 7 to 14 days. Every 24 h, 20 µL aliquots were taken for HPLC analysis.

### 3.5. Preliminary Cytotoxic Assay

MCF-7 human breast cancer cells were obtained from the American Type Culture Collection (ATCC; Rockville, MD, USA) and cultured in Eagle’s Minimum Essential Medium (MEM; Sigma-Aldrich, Darmstadt, Germany) supplemented with 10% fetal bovine serum (FBS; Sigma-Aldrich) and 1% penicillin/streptomycin mixture (10,000 U/mL penicillin and 10 mg/mL streptomycin; Sigma-Aldrich).

Stock solutions of the tested compounds were first prepared in DMSO at 200-fold the intended final concentration. They were then diluted in MEM supplemented with 10% (*v*/*v*) FBS, antibiotics and 0.00625–0.05% Tween 80 (*v*/*v*), resulting in a final DMSO concentration of 0.5%. Once the cells reached 80–90% confluence, they were harvested using 0.25% trypsin in 1 mM EDTA (Life Technologies, Warsaw, Poland) and seeded into 96-well plates at a density of 8 × 10^3^ cells per well. After overnight incubation at 37 °C in a humidified atmosphere with 5% CO_2_, the medium was replaced with 100 µL of freshly prepared medium containing the tested compounds. The cells were treated for 72 h under the same conditions.

Following treatment, 25 μL of MTT solution was added to each well, and the plates were incubated for 2 h at 37 °C. Subsequently, 100 µL of solubilizing solution (45 mL DMF, 13.5 g SDS, and 55 mL distilled water) was added to each well [102]. After overnight incubation at 37 °C, optical density was measured at 550 nm with a reference wavelength of 670 nm using a Varioskan Lux reader (Thermo Fisher Scientific, Waltham, MA, USA). IC₅₀ values, indicating the concentration required to reduce cell viability by 50% compared to untreated controls (set as 100% viability), were calculated using GraphPad Prism 9 software (GraphPad Software, San Diego, CA, USA). All data from this preliminary cytotoxic assay were analyzed by ANOVA followed by Tukey’s post hoc test for multiple comparisons, with significance set at *p* < 0.05. A similar statistical approach was used for the rest of the experimental work presented in this study.

### 3.6. Synergistic Cytotoxicity Assay

The synergistic effects of drug combinations were studied in various cancer cell lines, including MCF-7 (human breast cancer), A549 (human lung cancer), U-87 MG (human malignant glioma), HepG2 (human liver cancer), and HUH7 (human liver cancer). HMEC-1, an immortalized human microvascular endothelial cell line, was used as a model for normal cells. All cell lines were purchased from ATCC (Rockville, MD, USA).

The cancer cell lines were cultured in Dulbecco’s Modified Eagle’s Medium (DMEM; Sigma-Aldrich) supplemented with 10% FBS and 1% penicillin/streptomycin mixture (10,000 U/mL penicillin and 10 mg/mL streptomycin; Sigma-Aldrich) at 37 °C in a humidified atmosphere with 5% CO_2_. HMEC-1 cells were grown in MCDB 131 medium containing 10% FBS, 10 ng/mL EGF, 1 µg/mL hydrocortisone, and 1% penicillin/streptomycin mixture under the same incubation conditions. All cultures were routinely checked for mycoplasma contamination and passaged three times a week using 0.25% trypsin in 1 mM EDTA.

For the MTT assay, exponentially growing cells were seeded into 96-well plates at a density of 5 × 10³ cells per well. After 24 h of incubation, the medium was replaced with 100 µL of fresh medium containing the tested compounds. After 72 h of treatment, cell viability was assessed using the MTT assay as described earlier. Absorbance was measured at 580 nm with a reference wavelength of 720 nm using a PowerWave HT microplate reader (BioTek Instruments, Winooski, VT, USA). Synergistic effects were evaluated using CompuSyn v1.0 software [103]. IC_50_ values were calculated using GraphPad Prism 9 software (GraphPad Software).

### 3.7. Flow Cytometric Measurements

Cells were seeded in 60 mm diameter dishes at a density of 1 × 10^6^ cells per dish in 3 mL of DMEM supplemented with 10% FBS and 1% penicillin/streptomycin mixture (10,000 U/mL penicillin and 10 mg/mL streptomycin). After 24 h of incubation at 37 °C in a humidified atmosphere with 5% CO_2_, the medium was replaced with fresh medium containing the test drugs, prepared as described earlier.

After 72 h of drug treatment, cells were detached using 0.25% trypsin in 1 mM EDTA, centrifuged at 1000 rpm (RCF = 173 g) for 5 min at 4 °C in a Sigma 3K15 centrifuge (SIGMA Laborzentrifugen, Osterode am Harz, Germany), resuspended in 100 µL of ice-cold PBS (pH 7.4), and fixed with 1 mL of cold 70% ethanol. After fixation, cells were centrifuged at 2000 rpm (RCF = 693 g) for 10 min at 4 °C to remove the ethanol, washed with PBS, and centrifuged again under the same conditions. The final pellet was resuspended in 300 µL of PBS containing 4 mg/mL propidium iodide and 20 µg/mL RNase A.

Cell cycle analysis was performed using a BD LSR II™ flow cytometer (Becton Dickinson, San Jose, CA, USA), analyzing 10,000 cells to determine their distribution across different cell cycle phases. Data were analyzed using FlowJo v7.6 software (FlowJo, LLC, Ashland, OR, USA).

### 3.8. Doxorubicin Accumulation Assay

Intracellular accumulation of DOXO was measured based on its autofluorescent properties. Cells were seeded at a density of 2 × 10^4^ cells per well in black 96-well plates as described in Section 3.6. After 24 h of incubation at 37 °C in a humidified atmosphere with 5% CO₂, the medium was replaced with fresh medium containing 50 μM DOXO, varying concentrations of SUs (from 0 to 50 μM), 0.05% (*v*/*v*) Tween-80, and 0.5% (*v*/*v*) DMSO. After 3 h of incubation, the cells were washed twice with 100 µL of PBS (pH 7.4) and intracellular DOXO levels were quantified by measuring total fluorescence in each well using a Fluoroskan Ascent FL microplate reader (Labsystem, Stockholm, Sweden). The excitation and emission wavelengths were set at 485 nm and 538 nm, respectively, to closely match the absorption at 470 nm and emission at 560 nm of DOXO in biological systems [104].

### 3.9. Hemolysis and Membrane Integrity Evaluation

Blood samples were collected from healthy human volunteers into Vacutainer tubes containing 3% sodium citrate (BD Biosciences, Warsaw, Poland). Whole blood was centrifuged at 1500 rpm (RCF = 214 g) for 10 min in a CompactStar CS4 centrifuge (VWR) at RT to separate erythrocytes, which were washed twice with PBS (pH 7.4) and centrifuged at 1500 rpm for 5 min at RT after each wash. Hematocrit was measured to adjust the final RBC concentration in PBS (pH 7.4) to 40%. The drugs were then dissolved in the RBC suspension at the final concentrations outlined in Figure 8. RBCs incubated in dH_2_O were used as a positive control (100% hemolysis), while RBCs in PBS were used as the negative control. A blank sample of PBS without RBCs or drugs provided a baseline for absorbance measurements. Experimental samples, along with positive, negative, and vehicle (0.5% DMSO) controls, were incubated for 4 h at RT in the dark. After incubation, the samples were centrifuged at 1500 rpm for 5 min at RT, and the supernatant was collected to measure hemoglobin release using Drabkin’s method [87]. Absorbance was measured at 540 nm with a Jenway 6305 UV/Vis spectrophotometer (Bibby Scientific, Staffordshire, UK). This study was conducted in triplicate. The percentage of hemolysis was calculated by comparing the absorbance at 540 nm (OD_540_) of the test sample with that of the positive control using the following formula:

(2) $$\%\;\mathrm{Hemolysis}=\frac{{OD}_{540}\left(Test\right)-{OD}_{540}(Blank)}{{OD}_{540}\left(Total\;Lysis\right)-{OD}_{540}(Blank)}\times 100\%$$

### 3.10. In Vivo Experimental Procedures

Six- to eight-week-old female BALB/c mice (Charles River Breeding Laboratories, Wilmington, MA, USA) were bred at the Maria Sklodowska-Curie National Research Institute of Oncology, Gliwice Branch (Gliwice, Poland), under a HEPA-filtered Allentown’s IVC System (Allentown Caging Equipment Co., Allentown, NJ, USA). All efforts to minimize animal suffering were made by qualified personnel. This study was carried out in strict accordance with the recommendations in the Guide for the Care and Use of Laboratory Animals of the National Institutes of Health. The protocols were approved by the Committee on the Ethics of Animal Experiments of the Local Ethics Commission (Medical University of Silesia, Katowice, Poland; Permit No. 22/2024).

BALB/c mice were injected subcutaneously in the lower flank with 2 × 10^5^ 4T1 cells suspended in 100 μL PBS. Growing tumors were measured with calipers, and tumor volumes were determined using the following formula:

(3) $$\mathrm{Volume}={Width}^{2}\times Length\times 0.52$$

Mice with well-developed tumors were randomly divided into four treatment groups: control (untreated), GLIM (4 mg/kg, i.p.), DOXO (5 mg/kg, i.v.) alone, and combination therapy with GLIM (4 mg/kg, i.p.) and DOXO (5 mg/kg, i.v.). Each treatment was administered a total of five times.

## 4. Conclusions

SUs are generally considered safe drugs, with minimal cytotoxicity even at high micromolar concentrations. Our findings show that second-generation SUs are more cytotoxic than first-generation ones, with the latter often failing to reach IC_50_ values below 500 µM. To date, only Alkhalil et al. [10] have performed a comparative cytotoxicity evaluation of selected second-generation SUs (GLIM, GLIB, and GLIP) in multiple cell lines. In Figure S20, we compare their results with the limited data from other studies [11,16,26], and our own findings. The cytotoxicity of GLIM in our study (135.2–472.9 µM) aligns with the range reported by Alkhalil et al. and others (89.4–426.1 µM); however, we observed some cell line-specific differences. Such differences may be due to variations in culture media composition. For example, the surfactant we used to prevent drug aggregation may also influence cytotoxicity by affecting drug retention in certain cell lines.

**Figure 10:**
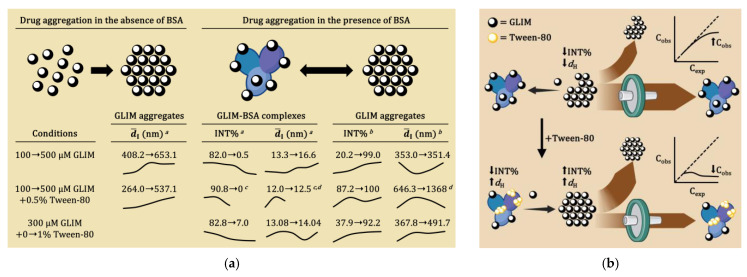
Effects of Tween-80 on GLIM aggregation in the absence and presence of BSA, and potential in vitro implications. Panel (**a**) illustrates aggregation parameters as a function of mixture composition, with initial and final values indicated. *^a^* Changes observed within the range of 0–500 µM GLIM; *^b^* changes observed within the range of 100–500 µM GLIM; *^c^* at GLIM concentrations exceeding 200 µM, the contribution of the BSA fraction became negligible; *^d^* initial and maximum values on the curve are provided. INT%—the relative contribution of specific size distributions to the total scattered light intensity in the sample. Panel (**b**) illustrates the potential implications of these findings for in vitro studies.

To explore the anticancer potential of SUs, we tested them in vitro at relatively high concentrations (100–500 µM) in culture media supplemented with 0.05% Tween-80 to prevent the formation of large drug aggregates. GLIM has a strong tendency to aggregate and precipitate in buffers and culture media, forming particles exceeding 300 nm in diameter (Figure 10a). Previous research by Shoichet demonstrated that such large aggregates can limit cellular availability and disrupt the biological activity of drugs. Similarly, our results suggest that BSA in the culture medium may also decrease the availability of strongly binding drugs like GLIM, thereby diminishing their biological activity and potentially leading to false-negative results.

We began investigating drug aggregation after observing that GLIM, which forms a visibly cloudy suspension in PBS at low micromolar levels, appeared to dissolve completely in culture medium even at high concentrations. Surprisingly, when we added 0.05% Tween-80 to this medium to prevent anticipated aggregation, the drug precipitated again, creating a cloudy mixture. Figure 10b proposes the potential mechanism for these observations. GLIM is quite lipophilic (logP = 3.81) [105] and binds extensively to plasma proteins (99.4% protein-bound in plasma) [106]. In culture media containing BSA, GLIM forms soluble drug–protein complexes of about 13.3–14.9 nm (${\bar{d}}_{I})$, which increase its observed concentration (C_obs_) and reduce tendency to aggregate compared to free (unbound) molecules. However, in media containing Tween-80, the surfactant competes with GLIM for binding to BSA, displacing the drug and increasing its free concentration. This increase in free drug concentration promotes aggregation due to overreaching critical aggregation concentration, ultimately decreasing the C_obs_ of the soluble drug. Like other researchers, we also found that BSA binding can diminish the activity of certain SUs in cell assays, such as cytotoxicity or the ability to block K_ATP_ channels [72]. In one of our experiments, the addition of 0.05% Tween-80 effectively displaced GLIM from BSA, resulting in a lower C_obs_ but significantly increasing its cytotoxicity against MCF-7 cells (viability dropped from 95.9% to 71.2%). In contrast, 0.05% Tween-80 had minimal impact on the cytotoxicity of CHLO (viability decrease by only 3.2%), likely due to its weaker binding to BSA. Although the effects of surfactants on the bioavailability of poorly soluble drugs are not fully understood, we suggest that their use may help avoid false-negative results and improve the reliability of drug screening assays for strongly protein-binding compounds.

We investigated the combined cytotoxic effects of DOXO and SUs in two stages. First, various combinations of DOXO with commercially available SUs were tested on MCF-7 breast cancer cells. While all combinations significantly reduced cell viability compared to DOXO alone, not all demonstrated synergistic effects. Combinations with first-generation SUs generally showed better combination indices with DOXO but were less cytotoxic compared to combinations involving second-generation SUs. In the second stage, we examined DOXO combined with selected SUs—specifically GLIM, GLIQ, and CHLO—across a broader panel of cell lines. Most cancer cell lines (A549, HepG2, MCF-7, and U-87MG) became more sensitive to the combined treatments, but some (HMEC-1 and HuH7) primarily showed antagonistic responses.

The observed synergy may result from SUs inhibiting MRPs, which normally expel anticancer drugs like DOXO from cancer cells. Since MRPs belong to the same ABC transporter subfamily (ABCC) as SURs—the clinical targets of SUs—it is plausible that SUs may also inhibit MRPs, thereby increasing DOXO retention inside cells. This mechanism has been previously demonstrated for GLIB, which inhibits MRP1 and MRP2, resulting in higher accumulation of their substrates in cancer cells [24]. Although this mechanism has not yet been confirmed for GLIM, GLIQ, and CHLO, our study showed that these SUs increased DOXO levels (up to 2.3 times) in sensitive cell lines, suggesting a similar mode of action. Synergistic effects were also seen in changes to the cell cycle—combinations with DOXO significantly increased the SubG1 and G2/M populations in sensitive cell lines, surpassing the effects of SUs or DOXO alone.

The most promising result in our study was observed with the DOXO + GLIM combination. Treatment with 0.12 µM DOXO and 45.1 µM GLIM resulted in a 4.4-fold increase in cytotoxicity against MCF-7 breast cancer cells, while also exhibiting cytoprotective effects in non-cancerous HMEC-1 cells. Preliminary in vivo studies demonstrated that this combination was significantly more effective at inhibiting 4T1 tumor growth in mice than monotherapies or the control group. Administering 5 mg/kg DOXO and 4 mg/kg GLIM improved efficacy by 32% compared to DOXO monotherapy. However, the combination therapy group experienced a 13% greater body weight loss than the DOXO-alone group by the end of treatment, indicating increased systemic toxicity. Importantly, this weight loss was reversible upon cessation of treatment.

We observed that co-administration of SUs and DOXO does not increase hemolysis. The combinations of GLIM, GLIQ, and CHLO with DOXO did not raise hemolysis above the level induced by DOXO alone (32.3%), indicating that SUs do not enhance DOXO’s toxicity to red blood cells. Clinically, this finding suggests that such combination therapies do not increase the risk of hemolytic anemia associated with DOXO, even in patients with underlying conditions such as diabetes or those receiving concurrent treatments.

Another clinically significant consideration when discussing combination therapies involving SUs is their potential to increase the risk of hypoglycemia, even in healthy individuals. Because SUs stimulate the pancreas to release insulin, they can lower blood sugar levels in both diabetic and non-diabetic individuals. However, not all SUs carry the same risk of hypoglycemia. In our study, the DOXO + GLIM combination demonstrated the most effective anticancer activity. At the same time, GLIM was associated with a lower risk of hypoglycemia compared to other SUs, both in diabetic patients (1–8 mg/day) [107,108] and in non-diabetic individuals [109]. In non-diabetic individuals, GLIM (4 mg) can still increase insulin and C-peptide levels even when blood sugar levels are normal, but it does not significantly interfere with the body’s natural hormonal defenses against low blood sugar. As a result, important counterregulatory responses—such as glucagon release and sympathetic activation—remain more intact if hypoglycemia occurs [110]. In other words, while GLIM lowers blood sugar in both groups, it poses a lower risk of severe hypoglycemia compared to other SUs. Moreover, GLIM may be a safer option for patients with cardiovascular disease because, unlike other SUs, it does not impair the ischemic preconditioning of cardiac myocytes [110].

SUs are widely used and popular treatments for T2DM, and the clinical use of newer agents like glimepiride is expected to continue, including in patients with concurrent cancers. While SUs are associated with drawbacks—including an increased risk of hypoglycemia, weight gain, and potential β-cell dysfunction—their potential to enhance the effectiveness of chemotherapeutic agents may outweigh these limitations. Our findings suggest that the synergistic effects of SUs, particularly GLIM, can improve the efficacy of DOXO-based cancer therapies and/or reduce their side effects. Future in vivo studies are planned to evaluate this therapeutic strategy in a human breast cancer model using immunodeficient BALB/c nude mice and to explore alternative formulations to minimize systemic toxicity. Additionally, further research is needed to confirm whether SUs can enhance the efficacy of other anticancer agents and to better understand the underlying mechanisms of their synergistic effects.

## Figures and Tables

**Table 1:** Summary of IC_50_ values for SUs determined by MTT assays.

Drug Name	IC_50_ Value in Specific Cell Line (µM)					
	A549	HepG2	HMEC-1	HuH7	MCF-7	U-87MG
2nd-generation SUs						
GLIM	367.7 ± 16.4	179.6 ± 21.6	135.5 ± 5.1	475.8 ± 7.0	180.3 ± 7.6	189.4 ± 14.8
GLIQ	259.1 ± 16.3	39.6 ± 2.6	156.6 ± 2.2	400.7 ± 2.2	68.8 ± 2.5	57.1 ± 1.8
GLIC		419.3 ± 53.2	447.7 ± 2.3	n.d.	n.d.	
GLIB		46.1 ± 1.0	248.0 ± 3.6	493.2 ± 1.3	86.6 ± 3.2	
GLIP		228.8 ± 10.9	454.1 ± 1.8	421.9 ± 0.5	339.0 ± 18.8	
1st-generation SUs						
CARB		70.7 ± 1.4	n.d.	n.d.	n.d.	
CHLO	n.d.	480.3 ± 54.1	218.2 ± 1.6	n.d.	n.d.	n.d.
TOLA		469.8 ± 12.4	468.6 ± 0.5	n.d.	n.d.	
TOLB		232.2 ± 21.6	88.2 ± 4.1	n.d.	460.9 ± 53.9	
Other compounds						
MESU		63.1 ± 0.3	78.3 ± 2.4	n.d.	495.9 ± 0.8	
DOXO	0.27 ± 0.02	0.30 ± 0.01	0.08 ± 0.01	0.28 ± 0.02	0.46 ± 0.06	0.57 ± 0.11

n.d.—Not determinable; indicates that the compound did not achieve the IC_50_ value within the range of concentrations tested. Results are presented as means ± SD (*n* = 3).

## Data Availability

The raw data supporting the conclusions of this article will be made available by the authors on request.

## Supplementary Materials

The following supporting information can be downloaded at: https://www.mdpi.com/article/10.3390/ijms26041429/s1.

## Abbreviations

BSA—Bovine serum albumin, CARB—Carbutamide, CDCF—5(6)-Carboxy-2′,7′-dichlorofluorescein, C_exp_—Concentration resulting from the total amount of drug added to the mixture, CHLO—Chlorpropamide, CI—Combination Index, C_obs_—Concentration measured after separation process, *d*_H_—hydrodynamic diameter, ${\bar{d}}_{I}$—intensity-weighted mean hydrodynamic diameter, ${\bar{d}}_{N}$—number-weighted mean hydrodynamic diameter, DCR—Derived Count Rate, DOXO—Doxorubicin, GLIB—Glibenclamide, GLIC—Gliclazide, GLIM—Glimepiride, GLIP—Glipizide, GLIQ—Gliquidone, HSA—Human serum albumin, i.p.—intraperitoneal, i.v.—intravenous, MCR—Mean Count Rate, MESU—*N*-(3-methoxy-propyl)-*N*’-(toluene-4-sulfoyl)-urea, RT—room temperature, s.c.—subcutaneous, T80—Tween-80, TOLA—Tolazamide, TOLB—Tolbutamide, ${nK}_{a}$—Global affinity constant.
