# Supplementary material for: Combining Sulfonylureas with Anticancer Drugs: Evidence of Synergistic Efficacy with Doxorubicin In Vitro and In Vivo
Source: Int J Mol Sci. 2025 Feb 8;26(4):1429. doi: 10.3390/ijms26041429 (PMC11855866; doi:10.3390/ijms26041429)
Supplement: Supplementary file 1 [file ijms-26-01429-s001.zip › ijms-3425333-supplementary.pdf]

Supplementary Materials

Combining Sulfonylureas with Anticancer Drugs: Evidence of Synergistic Efficacy with Doxorubicin In Vitro and In Vivo

Mateusz D. Tomczyk[1,*], Karolina Matczak[2], Marta Denel-Bobrowska[3], Grzegorz Dzido[4], Anna Kubicka[2], Daria Gendosz de Carrillo[5,6], Tomasz Cichoń[7], Marlena Golec[8], Beata Powieczko[1], Waldemar Rzetelny[9], Agnieszka B. Olejniczak[3], Horacio Pérez-Sánchez[10]

[1]*Department of Organic Chemistry, Bioorganic Chemistry and Biotechnology, Faculty of Chemistry, Silesian University of Technology, Krzywoustego 4, Gliwice, 44-100, Poland*

[2]*Department of Medical Biophysics, Faculty of Biology and Environmental Protection, University of Łódź, Pomorska 141/143, Łódź, 90-236, Poland*

[3]*Screening Laboratory, Institute of Medical Biology, Polish Academy of Sciences, Lodowa 106, Łódź, 93-232, Poland*

[4]*Department of Physical Chemistry and Technology of Polymers, Silesian University of Technology, Strzody 9, Gliwice, 44-100, Poland*

[5]*Department of Physiology, Faculty of Medical Sciences, Medical University of Silesia, Medyków 18, Katowice, 40-055, Poland*

[6]*Department of Histology and Cell Pathology, Faculty of Medical Sciences, Medical University of Silesia, Jordana 19, Zabrze, 41-808, Poland*

[7]*Center for Translational Research and Molecular Biology of Cancer, Maria Skłodowska-Curie National Re-search Institute of Oncology, Gliwice Branch, Wybrzeże Armii Krajowej Street 15, Gliwice, 44-102, Poland*

[8]*Department of Radiopharmacy and Preclinical PET Imaging, Maria Skłodowska-Curie National Research Institute of Oncology, Gliwice Branch, Wybrzeże Armii Krajowej Street 15, Gliwice, 44-102, Poland*

[9]*Department of Chemotherapy, Hospital of the Ministry of Interior and Administration in Łódź, Północna 42, Łódź, 91-425, Poland*

[10]*Computer Engineering Department, Structural Bioinformatics and High Performance Computing Research Group (BIO-HPC), Universidad Católica de Murcia (UCAM), Campus de los Jerónimos 135, Murcia, 30107, Spain*

* – Corresponding author: mateusz.d.tomczyk@polsl.pl

Figure S1. Structures and key information of studied compounds....................................p. 3

Figure S2. Literature summary on solubility and bioavailability of SUs..........................p. 4–5

Figure S3. DLS results for GLIM at various concentrations (100–500 μM) in PBS (pH 7.4) at 37 °C .... p. 6
Figure S4. DLS results for GLIM at various concentrations (100–500 μM) in PBS (pH 7.4) with 0.5% Tween-80 at 37 °C .... p. 7
Figure S5. Summary of DLS results for GLIM samples .... p. 8–9
Figure S6. HPLC analysis of BSA in separated samples .... p. 10
Figure S7. Stability analysis of drugs and their mixtures .... p. 11
Figure S8. Viability charts and curves for IC_{50} calculation .... p. 12–14
Figure S9. Cytotoxicity of DOXO+SUs combinations on MCF-7 cell line .... p. 15
Figure S10. Synergistic effects of DOXO+SUs combinations on MCF-7 cell line .... p. 16
Figure S11. Median-effect plots of DOXO+SUs combinations on MCF-7 cell line .... p. 17–20
Figure S12. Commentary on median-effect plots for the DOXO+CARB combination .... p. 21
Figure S13. Cytotoxicity results for GLIM, GLIQ, and CHLO on various cell lines .... p. 22–23
Figure S14. Synergistic effects of GLIM, GLIQ, and CHLO on various cell lines .... p. 24–25
Figure S15. Median-effect plots of GLIM, GLIQ and CHLO on various cell lines .... p. 26–31
Figure S16. Cell cycle analysis for GLIM, GLIQ, CHLO, and DOXO treatments .... p. 32
Figure S17. Retention times (t_R), separation conditions, and HPLC analysis details .... p. 33
Figure S18. Chromatogram of DOXO analysis .... p. 34
Figure S19. Chromatogram of BSA analysis .... p. 34
Figure S20. Comparative cytotoxicity of SUs from various studies .... p. 35

Figure S1. Structures and key information of studied compounds

Structure	Formula	Mol. wt.	Generic name	Abbrev.	Brand name
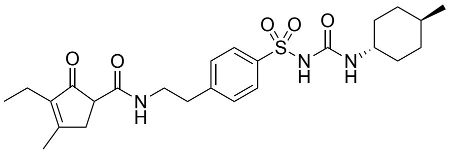	$C_{25}H_{35}N_3O_5S$	489.63	Glimepiride	GLIM	Amaryl
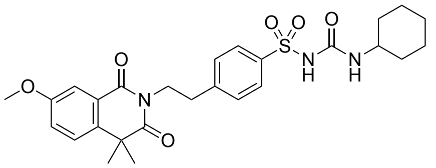	$C_{27}H_{33}N_3O_6S$	527.64	Gliquidone	GLIQ	Glurenorm
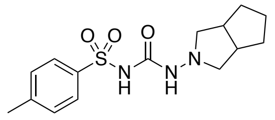	$C_{15}H_{21}N_3O_3S$	323.41	Gliclazide	GLIC	Diamicron
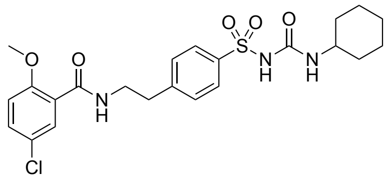	$C_{23}H_{28}ClN_3O_5S$	494.00	Glibenclamide<br>Glyburide	GLIB	Diabeta<br>Glucovance<br>Glynase
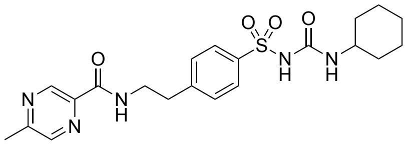	$C_{21}H_{27}N_5O_4S$	445.54	Glipizide	GLIP	Glucotrol
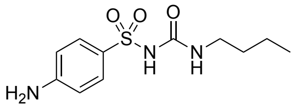	$C_{11}H_{17}N_3O_3S$	271.34	Carbutamide	CARB	Glucidoral
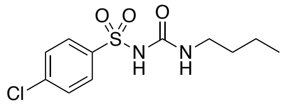	$C_{11}H_{15}ClN_2O_3S$	290.76	Chlorpropamide	CHLO	Diabinese
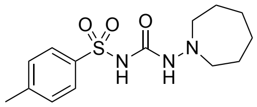	$C_{14}H_{21}N_3O_3S$	311.40	Tolazamide	TOLA	Tolinase
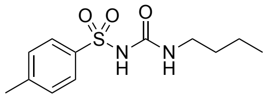	$C_{12}H_{18}N_2O_3S$	270.35	Tolbutamide	TOLB	Orinase
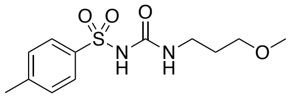	$C_{12}H_{18}N_2O_4S$	286.35	*N*-(3-methoxy-propyl)-*N'*-(toluene-4-sulfonyl)-urea	MESU	*Not a drug*

Figure S2. Literature summary on solubility and bioavailability of SUs

Drug	Solubility (conditions)	C_{max} (t_{max}, dose, route)	Drug-HSA global affinity constant (conditions)
GLIM	8.63 μg/mL = 17.59 μM (pH 7.4 PBS, 25 °C)[39]	0.55 μg/mL = 1.12 μM (2.9 h after 8 mg, PO)[42]	9.1×10^5 M^{-1} (pH 7.4 PB, 37 °C)[45]
	6.40 μg/mL = 13.04 μM (water, 25 °C)[39]	0.24 μg/mL = 0.49 μM (after 1.0 mg, IV)[43]	
	6.00 μg/mL = 12.23 μM (water, 37 °C)[40]	0.09 μg/mL = 0.18 μM (2.7 h after 1 mg, PO)[43]	
	0.56 μg/mL = 1.14 μM (water, 35 °C)[41]	0.19 μg/mL = 0.39 μM (4 h after 2 mg, PO)[44]	
	0.49 μg/mL = 1.00 μM (pH 7.2 PBS, 35 °C)[41]		
GLIQ	5.07 μg/mL = 9.61 μM (pH 7.4 PBS, 37 °C)[46]	0.60 μg/mL = 1.14 μM (2–3 h after 30 mg, PO)[48]	
	4.00 μg/mL = 7.58 μM (water, 37 °C)[47]		
	0.14 μg/mL = 0.27 μM (water, 37 °C)[46]		
GLIC	2.10 mg/mL = 6.49 mM (pH 7.4 PBS, 37 °C)[49]	15.0 μg/mL = 46.38 μM (2.8 h after 160 mg, PO)[52]	0.73×10^5 M^{-1} (pH 7.4 PB, 37 °C)[54]
	1.17 mg/mL = 3.62 mM (pH 7.4 PBS, 37 °C)[50]	3.9–6.3 μg/mL = 5.87–19.48 μM (4–6 h after 80 mg, PO)[53]	0.54×10^5 M^{-1} (pH 7.4 PB, 37 °C)[55]
	0.28 mg/mL = 0.87 mM (water pH 6.2, rt)[51]		

[39] Seedher, N.; Kanojia, M. Co-solvent solubilization of some poorly-soluble antidiabetic drugs. *Pharm. Dev. Technol.* **2009**, *14*, 185–192. https://doi.org/10.1080/10837450802498894.

[40] Rajesh, S.Y.; Singh, S.K.; Pandey, N.K.; Sharma, P.; Bawa, P.; Kumar, B.; Gulati, M.; Jain, S.K.; Gowthamarajan, K.; Singh, S. Impact of various solid carriers and spray drying on pre/post compression properties of solid SNEDDS loaded with glimepiride: in vitro-ex vivo evaluation and cytotoxicity assessment. *Drug Dev. Ind. Pharm.* **2018**, *44*, 1056–1069. https://doi.org/10.1080/03639045.2018.1431656.

[41] Pal, A.; Roy, S.; Kumar, A.; Mahmood, S.; Khodapanah, N.; Thomas, S.; Agatemor, C.; Ghosal, K. Physicochemical Characterization, Molecular Docking, and In Vitro Dissolution of Glimepiride-Captisol Inclusion Complexes. *ACS Omega* **2020**, *32*, 19968–19977. https://doi.org/10.1021/acsomega.0c01228.

[42] Shukla, U.A.; Chi, E.M.; Lehr, K.-H. Glimepiride Pharmacokinetics in Obese Versus Non-Obese Diabetic Patients. *Ann. Pharmacother.* **2004**, *38*, 30–35. https://doi.org/10.1345/aph.1C397.

[43] Badian, M.; Korn, A.; Lehr, K.-H.; Malerczyk, V.; Waldhäusl, W. Absolute bioavailability of glimepiride (Amaryl) after oral administration. *Drug Metabol. Drug Interact.* **1994**, *11*, 331–339. https://doi.org/10.1515/dmdi.1994.11.4.331.

[44] Yun, H.-Y.; Park, H.-C.; Kang, W.; Kwon, K.-I. Pharmacokinetic and pharmacodynamic modelling of the effects of glimepiride on insulin secretion and glucose lowering in healthy humans. *J. Clin. Pharm. Ther.* **2006**, *31*, 469–476. https://doi.org/10.1111/j.1365-2710.2006.00766.x.

[45] Yang, B.; Zheng, X.; Hage, D.S. Binding studies based on ultrafast affinity extraction and single- or two-column systems: Interactions of second- and third-generation sulfonylurea drugs with normal or glycated human serum albumin. *J. Chromatogr. B* **2018**, *1102–1103*, 8–16. https://doi.org/10.1016/j.jchromb.2018.10.015.

[46] Miro, A.; Quaglia, F.; Sorrentino, U.; Rotonda, M.L.; Bianca, R.D.D.V.; Sorrentino, R. Improvement of gliquidone hypoglycaemic effect in rats by cyclodextrin formulations. *Eur. J. Pharm. Sci.* **2004**, *23*, 57–64. https://doi.org/10.1016/j.ejps.2004.05.008.

[47] Jia, L.; Yu, Y.; Wang, M.; Ouyang, H.; Gong, J. Preparation and characterization for multicomponent crystals of the antidiabetic drug gliquidone based on crystal engineering. *CrystEngComm* **2019**, *21*, 1617–1625. https://doi.org/10.1039/C8CE01848K.

[48] Kopitar, Z.; Koss, F.W. Pharmakokinetisches Verhalten von Gliquidone (AR-DF 26), einem neuen Sulfonylharnstoff (Zusammenfassung der bisherigen Untersuchungen) [Pharmacokinetic behaviour of gliquidone (AR-DF 26), a new sulfonyl urea. Summary of the studies so far (author's transl)]. *Arzneimittelforschung* **1975**, *25*, 1933–1938.

[49] Chadha, R.; Rani, D.; Goyal, P. Novel cocrystals of gliclazide: characterization and evaluation. *CrystEngComm* **2016**, *18*, 2275–2283. https://doi.org/10.1039/C5CE02402A.

[50] Samie, A.; Desiraju, G.R.; Banik, M. Salts and Cocrystals of the Antidiabetic Drugs Gliclazide, Tolbutamide, and Glipizide: Solubility Enhancements through Drug–Coformer Interactions. *Cryst. Growth Des.* **2017**, *17*, 2406–2417. https://doi.org/10.1021/acs.cgd.6b01804.

[51] Ibrahim, A.Y.; El-Malah, Y.; Abourehab, M.A.S.; Aldawsari, H. Solubility Enhancement of Gliclazide via Co-crystallization with Malonic Acid. *Life Sci. J.* **2019**, *16*, 49–53. https://doi.org/10.7537/marslsj160119.06.

[52] Davis, T.M.E.; Daly, F.; Walsh, J.P.; Ilett, K.F.; Beilby, J.P.; Dusci, L.J.; Barrett, P.H.R. Pharmacokinetics and pharmacodynamics of gliclazide in Caucasians and Australian Aborigines with type 2 diabetes. *Br. J. Clin. Pharmacol.* **2000**, *49*, 223–230. https://doi.org/10.1046/j.1365-2125.2000.00162.x.

[53] Campbell, D.B.; Lavielle, R.; Nathan, C. The mode of action and clinical pharmacology of gliclazide: a review. *Diabetes Res. Clin. Pract.* **1991**, *14*, 21–36. https://doi.org/10.1016/0168-8227(91)90005-X.

[54] Zheng, X.; Matsuda, R.; Hage, D.S. Analysis of free drug fractions by ultrafast affinity extraction: Interactions of sulfonylurea drugs with normal or glycated human serum albumin. *J. Chromatogr. A* **2014**, *1371*, 82–89. https://doi.org/10.1016/j.chroma.2014.10.092.

[55] Poddar, S.; Woolfork, A.G.; Iftekhar, S.; Ovbude, S.T.; Hage, D.S. Characterization of binding by sulfonylureas with normal or modified human serum albumin using affinity microcolumns prepared by entrapment. *J. Chromatogr. B* **2023**, *1226*, 123798. https://doi.org/10.1016/j.jchromb.2023.123798.

Figure S2 cont. Literature summary on solubility and bioavailability of SUs

Drug	Solubility (conditions)	C_{max} (t_{max}, dose, route)	Drug-HSA global affinity constant (conditions)
GLIB	10.10 μg/mL = 20.44 μM (pH 7.4 PBS, 37 °C)[56]	0.59 μM = 1.19 μM (3.4 h after 5 mg, PO)[57]	21.1×10^5 M^{-1} (pH 7.4 PB, 37 °C)[54]
	8.22 μg/mL = 16.64 μM (pH 7.4 PBS, 25 °C)[39]	0.14–0.32 μg/mL = 0.28–0.65 μM (0.9–3 h after 2.5 mg, PO)[58]	20.9×10^5 M^{-1} (pH 7.4 PB, 37 °C)[45]
	5.96 μg/mL = 12.06 μM (water, 25 °C)[39]	0.11 μg/mL = 0.22 μM (4.8 h after 3.5 mg, PO)[59]	15.1×10^5 M^{-1} (pH 7.4 PB, 37 °C)[55]
GLIP	0.19 mg/mL = 0.43 mM (pH 7.4 PBS, 37 °C)[60]	0.70 μg/mL = 1.57 μM (2.1 h after 5 mg, PO)[57]	5.4×10^5 M^{-1} (pH 7.4 PB, 37 °C)[45]
	66.48 μg/mL = 0.15 mM (pH 7.4 PBS, 25 °C)[39]	0.47 μg/mL = 1.05 μM (2.1 h after 5 mg, PO)[62]	1.43×10^5 M^{-1} (pH 7.4 PB, 37 °C)[55]
	10.23 μg/mL = 22.96 μM (water, 25 °C)[39]	0.32 μg/mL = 0.72 μM (1.8 h after 20 mg, PO)[63]	
	1.35 μg/mL = 3.03 μM (water, rt)[61]		
CARB	0.54 mg/mL = 1.99 mM (water, 37 °C)[64]	0.10–0.15 mg/mL = 0.37–0.55 mM (2.1 h after 2.5 g, PO)[65]	
CHLO	2.10 mg/mL = 7.59 mM (pH 7.4 PBS, rt)[66]	28.5 μg/mL = 0.10 mM (1–3 h after 250 mg, PO)[69]	0.62×10^5 M^{-1} (pH 7.4 PB, 37 °C)[55]
	0.28 mg/mL = 1.01 mM (water, 37 °C)[67]	100 μM (3.2 h after 250 mg, PO)[57]	
	0.17 mg/mL = 0.61 mM (water, 25 °C)[68]		
TOLA	2.30 mg/mL = 7.39 mM (pH 7.4 PBS, 25 °C)[69]	7.7–27.8 μg/mL = 24.73–89.27 μM (3.3–7.0 h after 500 mg, PO)[70]	0.33×10^5 M^{-1} (pH 7.4 PB, 37 °C)[55]
TOLB	4.18 mg/mL = 15.46 mM (pH 7.4 PBS, 37 °C)[50]	46.9–50.4 μg/mL = 0.17–0.19 mM (2 h after 15 mg/kg, PO)[71]	1.0×10^5 M^{-1} (pH 7.4 PB, 37 °C)[54]
	2.00 mg/mL = 7.40 mM (pH 7.4 PBS, rt)[66]	153 μM (3.4 h after 500 mg, PO)[57]	1.14×10^5 M^{-1} (pH 7.4 PB, 37 °C)[55]

[56] Goya, P.; Ran, D.; Chadha, R. Crystal Engineering: A Remedy to Tailor the Biopharmaceutical Aspects of Glibenclamide. *Cryst. Growth Des.* **2018**, *18*, 105–118. https://doi.org/10.1021/acs.cgd.7b00933.
[57] Sartor, G.; Melander, A.; Scherstén, B.; Wåhlin-Boll, E. Comparative Single-Dose Kinetics and Effects of Four Sulfonylureas in Healthy Volunteers. *Acta Med. Scand.* **1980**, *208*, 301–307. https://doi.org/10.1111/j.0954-6820.1980.tb01199.x.
[58] Jaber, L.A.; Antal, E.J.; Welshman, I.R. Pharmacokinetics and Pharmacodynamics of Glyburide in Young and Elderly Patients with Non-Insulin-Dependent Diabetes Mellitus. *Ann. Pharmacother.* **1996**. *30*, 472–475. https://doi.org/10.1177/106002809603000507.
[59] Shaheen, O.; Othman, S.; Jalal, I.; Awidi, A.; Al-Turk, W. Comparison of pharmacokinetics and pharmacodynamics of a conventional and a new rapidly dissolving glibenclamide preparation. *Int. J. Pharm.* **1987**, *38*, 123–131. https://doi.org/10.1016/0378-5173(87)90107-4.
[60] Batisai, E. Solubility Enhancement of Antidiabetic Drugs Using a Co-Crystallization Approach. *ChemistryOpen* **2021**, *10*, 1260–1268. https://doi.org/10.1002/open.202100246.
[61] Pandey, N.K.; Sehal, H.R.; Garg, V.; Gaur, T.; Kumar, B.; Singh, S.K.; Gulati, M.; Gowthamarajan, K.; Bawa, P.; Rajesh, S.Y.; Sharma, P.; Narang, R. Stable Co-crystals of Glipizide with Enhanced Dissolution Profiles: Preparation and Characterization. AAPS PharmSciTech 2017, 18, 2454–2465. https://doi.org/10.1208/s12249-017-0727-z.
[62] Kradjan, W.A.; Kobayashi, K.A.; Bauer, L.A.; Horn, J.R.; Opheim, K.E.; Wood, F.J. Glipizide Pharmacokinetics: Effects of Age, Diabetes, and Multiple Dosing. *J. Clin. Pharmacol.* **1989**, *29*, 1121–1127. https://doi.org/10.1002/j.1552-4604.1989.tb03289.x.
[63] Jaber, L.A.; Antal, E.J.; Slaughter, R.L.; Welshman, I.R. The pharmacokinetics and pharmacodynamics of 12 weeks of glyburide therapy in obese diabetics. *Eur. J. Clin. Pharmacol.* **1993**, *45*, 459–463. https://doi.org/10.1007/BF00315518.
[64] Yalkowsky S.H.; Dannenfelser, R.M. The AQUASOL dATAbASE of Aqueous Solubility. Fifth ed, Tucson, AZ: Univ AZ, College of Pharmacy (1992).
[65] Ridolfo, A.S.; Kirtley, W.R. Clinical experiences with carbutamide, an orally given hypoglycemic agent; preliminary report. *JAMA* **1956**, *160*, 1285–1288. https://doi.org/10.1001/jama.1956.02960500015005.
[66] Sarraguça, M.C.; Ribeiro, P.R.S.; Nunes, C.; Seabra, C.L. Solids Turn into Liquids – Liquid Eutectic Systems of Pharmaceutics to Improve Drug Solubility. *Pharmaceuticals* **2022**, *15*, 279. https://doi.org/10.3390/ph15030279.
[67] Dodge, A.; Gould, P.L. Dissolution of Chlorpropamide Tablets in A Methanol-Water Binary Solvent System. *Drug Dev. Ind. Pharm.* **1987**, *13*, 1817–1826. https://doi.org/10.3109/03639048709068694.
[68] Fadda, H.M.; Chen, X.; Aburub, A.; Mishra, D.; Pinal, R. A Novel Method for Determining the Solubility of Small Molecules in Aqueous Media and Polymer Solvent Systems Using Solution Calorimetry. *Pharm. Res.* **2014**, *31*, 1735–17453. https://doi.org/10.1007/s11095-013-1278-y.
[69] Taylor, J.A. Pharmacokinetics and biotransformation of chlorpropamide in man. *Clin. Pharmacol. Ther.* **1972**, *13*, 710–718. https://doi.org/10.1002/cpt1972135part1710.
[70] Kuldipkumar, A.; Tan, Y.T.F.; Goldstein, M.; Nagasaki, Y.; Zhang, G.G.Z.; Kwon, G.S. Amphiphilic Block Copolymer as a Crystal Habit Modifier. *Cryst. Growth Des.* **2005**, *5*, 1781–1785. https://doi.org/10.1021/cg050049m.
[71] Welling, P.G.; Patel, R.B.; Patel, U.R.; Gillespie, W.R.; Craig, W.A.; Albert, K.S. Bioavailability of tolazamide from tablets: comparison of in vitro and in vivo results. *J. Pharm. Sci.* **1982**, *71*, 1259–1263. https://doi.org/10.1002/jps.2600711119.

Figure S3. DLS results for GLIM at various concentrations (100–500 μM) in PBS (pH 7.4) at 37 °C

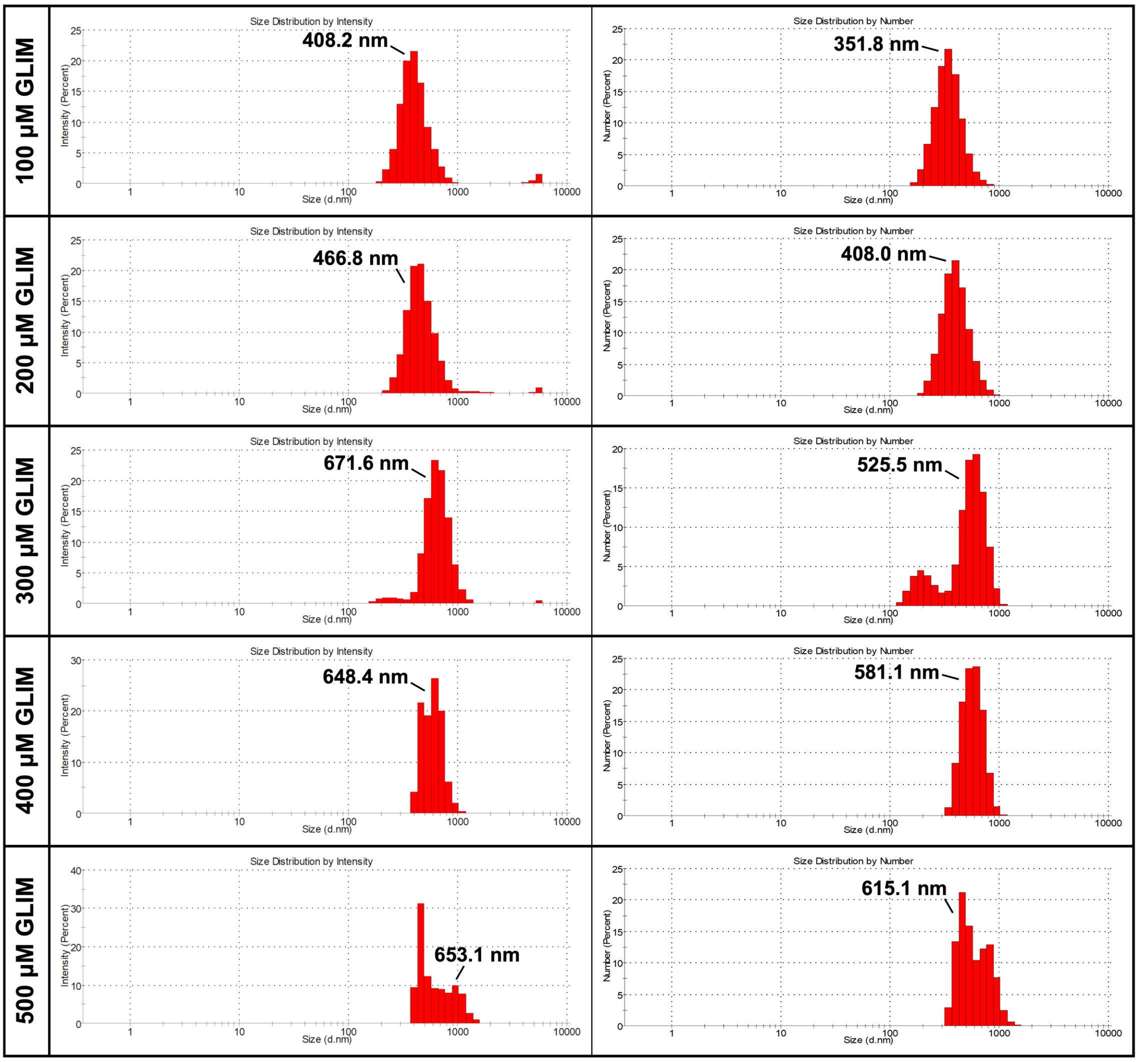

Figure S4. DLS results for GLIM at various concentrations (100–500 μM) in PBS (pH 7.4) with 0.5% Tween-80 at 37 °C

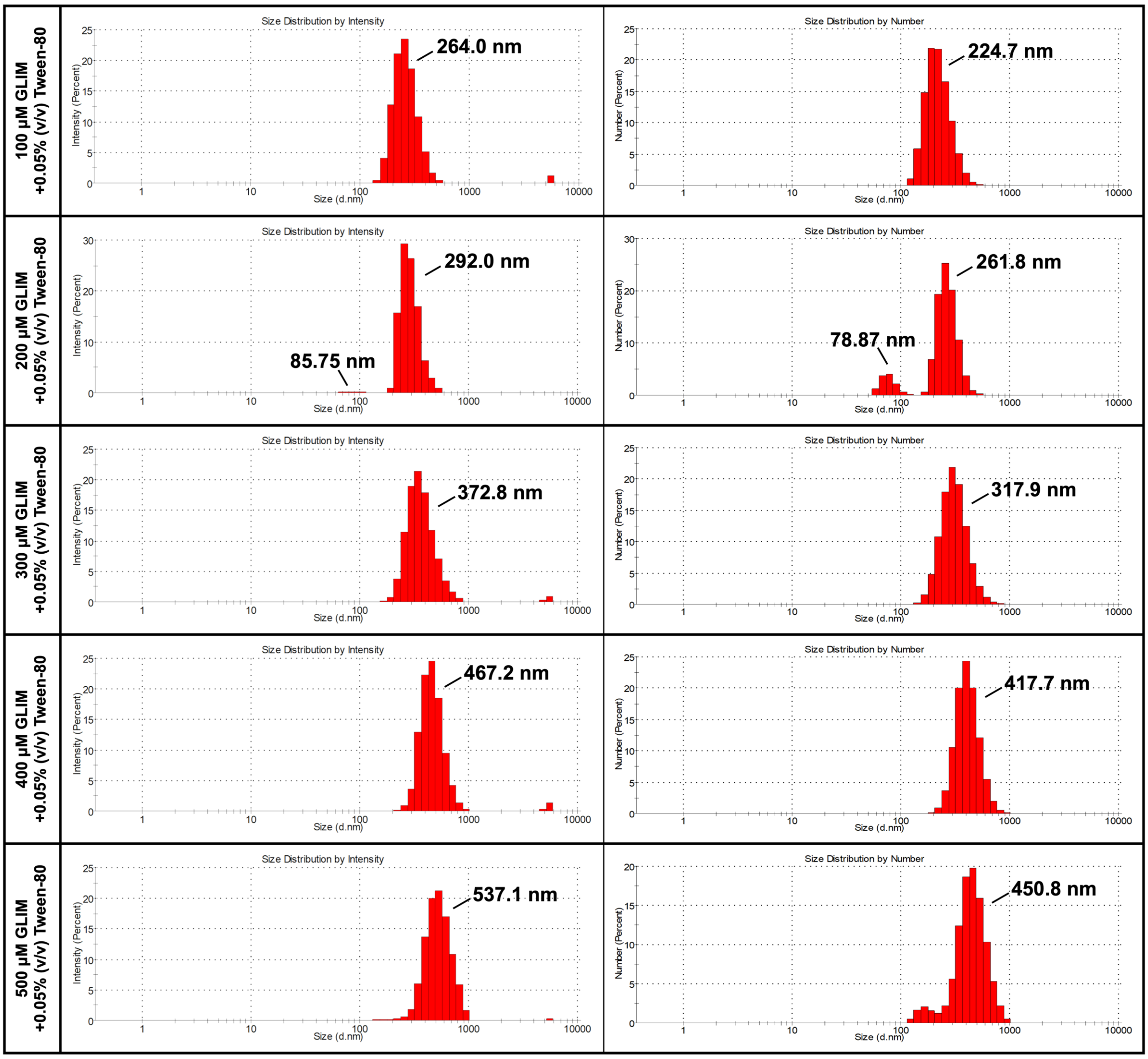

Figure S5. Summary of DLS results for GLIM samples

GLIM (μM)	BSA (mg/mL)	Tween-80 (v/v.%)	Z-Ave (d.nm)	PdI	Pk1 Mean Int (d.nm)	Pk2 Mean Int (d.nm)	Pk3 Mean Int (d.nm)	Pk1 Area Int (%)	Pk2 Area Int (%)	Pk3 Area Int (%)	Diffusion Coefficient (μ^2/s)	Intensity Mean d.nm	Volume Mean d.nm	Number Mean d.nm	DCR kcps	MCR kcps
100	0.0	0.0000	658.5	0.605	408.2	5215	0.000	97.8	2.2	0.0	1.01	516.2	604.7	351.8	36.0	42.0
200			899.6	0.744	466.8	1426	5462	97.7	1.3	1.0	0.736	524.9	572.5	408.0	55.1	60.4
300			1110	0.707	671.6	248.8	5560	95.4	4.2	0.4	0.596	674.7	695.3	525.5	190.1	147.5
400			2297	0.971	648.4	483.7	0.000	62.2	37.8	0.0	0.288	596.7	602.9	581.1	129.0	43.0
500			3008	0.915	546.1	1006	0.000	73.2	26.8	0.0	0.220	657.0	636.7	615.1	634.7	242.6
100	0.0	0.0500	343.5	0.509	264.0	5560	0.000	98.9	1.1	0.0	1.93	324.4	412.4	224.7	111.7	114.8
200			601.2	0.719	292.0	85.75	0.000	99.3	0.7	0.0	1.10	290.6	291.9	247.6	97.7	89.3
300			455.9	0.419	372.8	5358	0.000	98.9	1.1	0.0	1.45	429.8	494.8	317.9	312.4	321.0
400			627.3	0.462	467.2	5424	0.000	98.4	1.6	0.0	1.06	548.6	618.4	417.7	415.1	431.0
500			681.8	0.456	537.1	5560	0.000	99.8	0.2	0.0	0.971	548.8	579.6	450.8	695.4	210.1
0	4.5	0.0000	14.58	0.336	13.28	271.8	4130	82.0	15.3	2.7	45.4	165.6	9.475	7.579	59.0	59.7
100			14.82	0.398	13.01	265.6	4520	80.2	14.5	5.3	44.7	289.7	9.324	7.398	68.9	68.1
200			17.83	0.444	12.84	353.0	4489	70.0	20.2	5.7	37.1	338.7	9.474	7.580	76.3	76.0
300			80.65	0.418	14.71	313.5	5070	58.7	38.5	2.8	8.21	274.7	9.426	7.641	111.7	111.9
400			171.8	0.767	323.1	14.93	5094	83.6	13.2	3.2	3.85	433.0	11.37	8.128	442.3	122.4
500			307.0	0.212	351.4	5239	0.000	99.5	0.5	0.0	2.16	376.7	429.7	238.2	3324.7	375.0
300	4.5	0.0000	16.33	0.299	13.08	4293	401.2	82.8	8.7	7.1	40.5	414.1	9.478	7.485	54.7	54.3
		0.0125	115.7	0.434	14.17	367.8	5153	59.1	37.9	3.0	5.72	302.6	9.141	7.151	95.9	102.8
		0.0250	351.6	0.770	473.7	13.94	113.5	75.7	23.5	0.8	1.88	466.4	10.81	7.474	321.8	374.3
		0.0500	418.3	0.741	468.5	12.42	5108	88.4	10.0	1.6	1.58	499.7	14.26	8.705	534.9	152.2
		0.1000	510.8	0.592	491.7	14.04	5468	92.2	7.0	0.8	1.30	574.7	16.22	9.573	711.7	205.3
0	4.5	0.0500	40.17	0.236	12.00	160.7	5009	90.8	5.4	2.5	16.5	154.1	9.621	8.066	72.5	73.1
100			19.55	0.249	12.49	250.7	5036	88.7	5.7	5.0	33.9	278.6	9.850	8.041	71.3	71.8
200			1168	0.949	646.3	12.31	0.000	87.2	12.8	0.0	0.567	565.4	14.61	10.14	574.5	177.0
300			2673	0.553	1368	0.000	0.000	100.0	0.0	0.0	0.248	1368	1383	1273	1235.9	470.4
400			1821	0.504	1203	4665	0.000	94.7	5.3	0.0	0.363	1377	1434	901.0	3203.8	399.7
500			569.7	0.140	659.7	0.000	0.000	100.0	0.0	0.0	1.16	659.7	642.9	482.0	2258.7	238.6

Figure S5 cont. Summary of DLS results for GLIM samples

CHLO (µM)	BSA (mg/mL)	Tween-80 (v/v.%)	Z-Ave (d.nm)	PdI	Pk1 Mean Int (d.nm)	Pk2 Mean Int (d.nm)	Pk3 Mean Int (d.nm)	Pk1 Area Int (%)	Pk2 Area Int (%)	Pk3 Area Int (%)	Diffusion Coefficient (μ^2/s)	Intensity Mean d.nm	Volume Mean d.nm	Number Mean d.nm	DCR kcps	MCR kcps
0	4.5	0.0000	14.19	0.347	13.02	206.6	4360	83.6	12.4	4.0	46.6	212.1	9.729	7.889	42.5	41.9
100			14.15	0.339	13.25	236.9	3775	82.5	13.2	4.3	46.8	204.4	9.473	7.636	52.7	54.0
200			14.58	0.380	13.31	272.0	4230	81.9	15.4	2.8	45.4	165.6	9.476	7.579	59.0	59.7
300			13.19	0.292	13.49	258.1	4123	89.3	9.0	1.7	50.2	103.3	9.074	7.160	55.7	56.7
400			13.05	0.250	13.77	605.1	4254	91.3	7.1	1.6	50.7	121.1	9.393	7.414	50.6	52.0
500			14.13	0.347	12.91	216.2	3953	82.0	14.1	3.8	46.8	192.6	9.646	7.864	57.8	60.0

Figure S6. HPLC analysis of BSA in separated samples

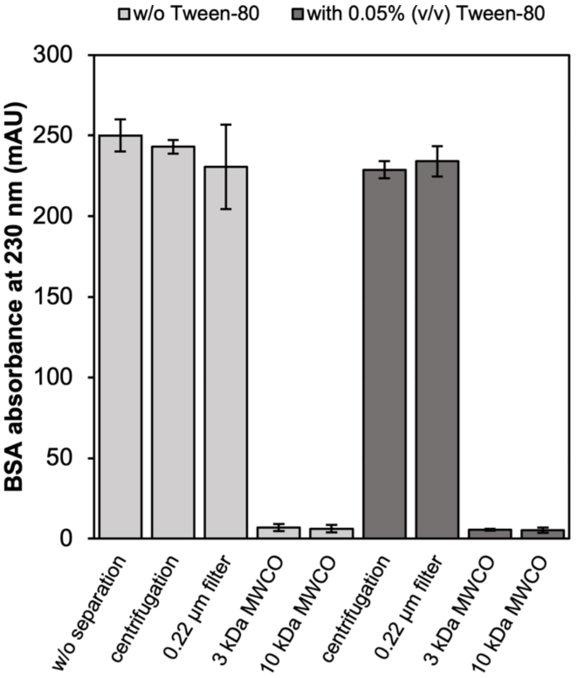

The experiment was conducted in triplicate, each time with three unique samples containing 4.5 mg/mL BSA. Results are presented as mean ± SD.

Figure S7. Stability analysis of drugs and their mixtures

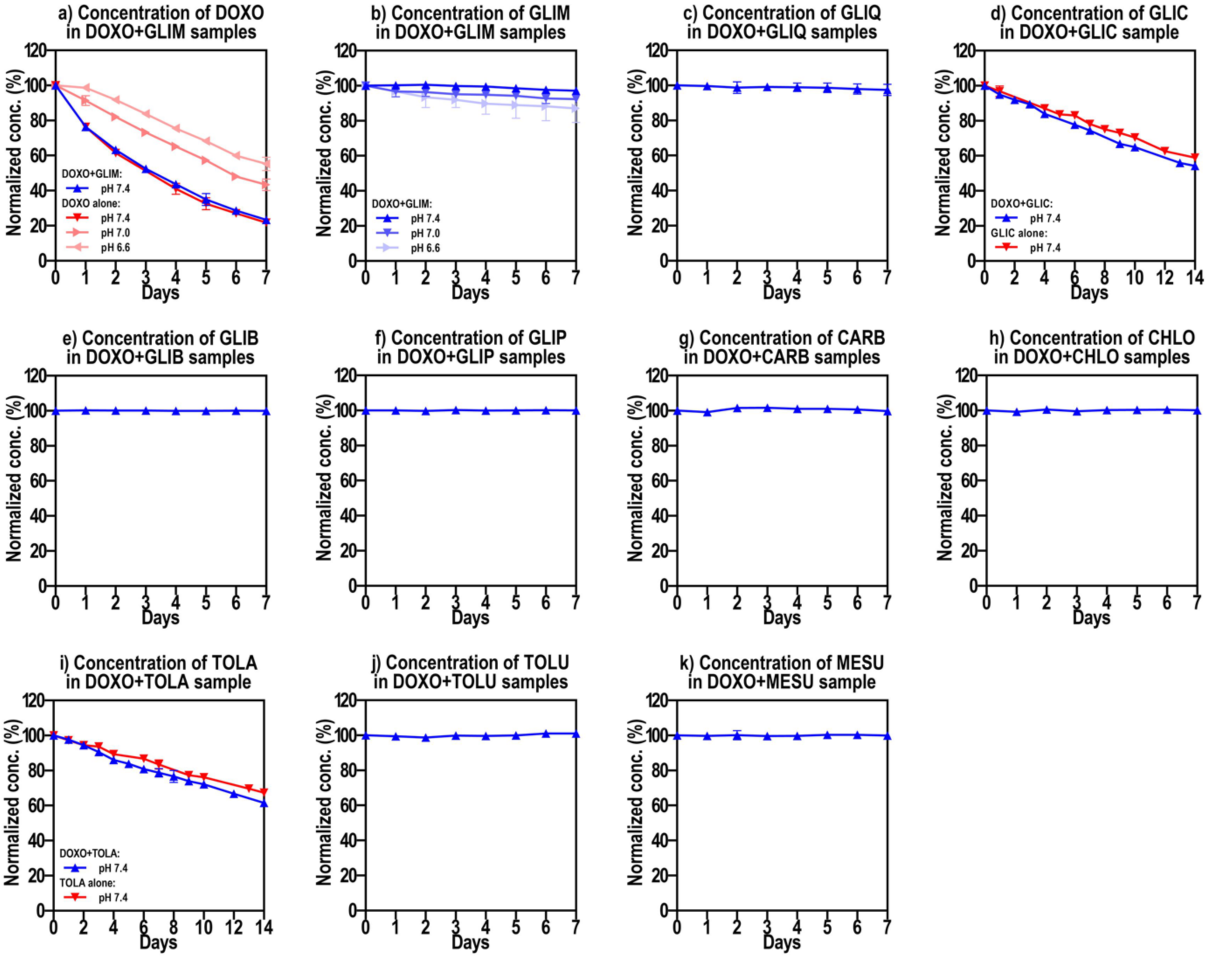

Figure S8. Viability charts and curves for IC_{50} calculation

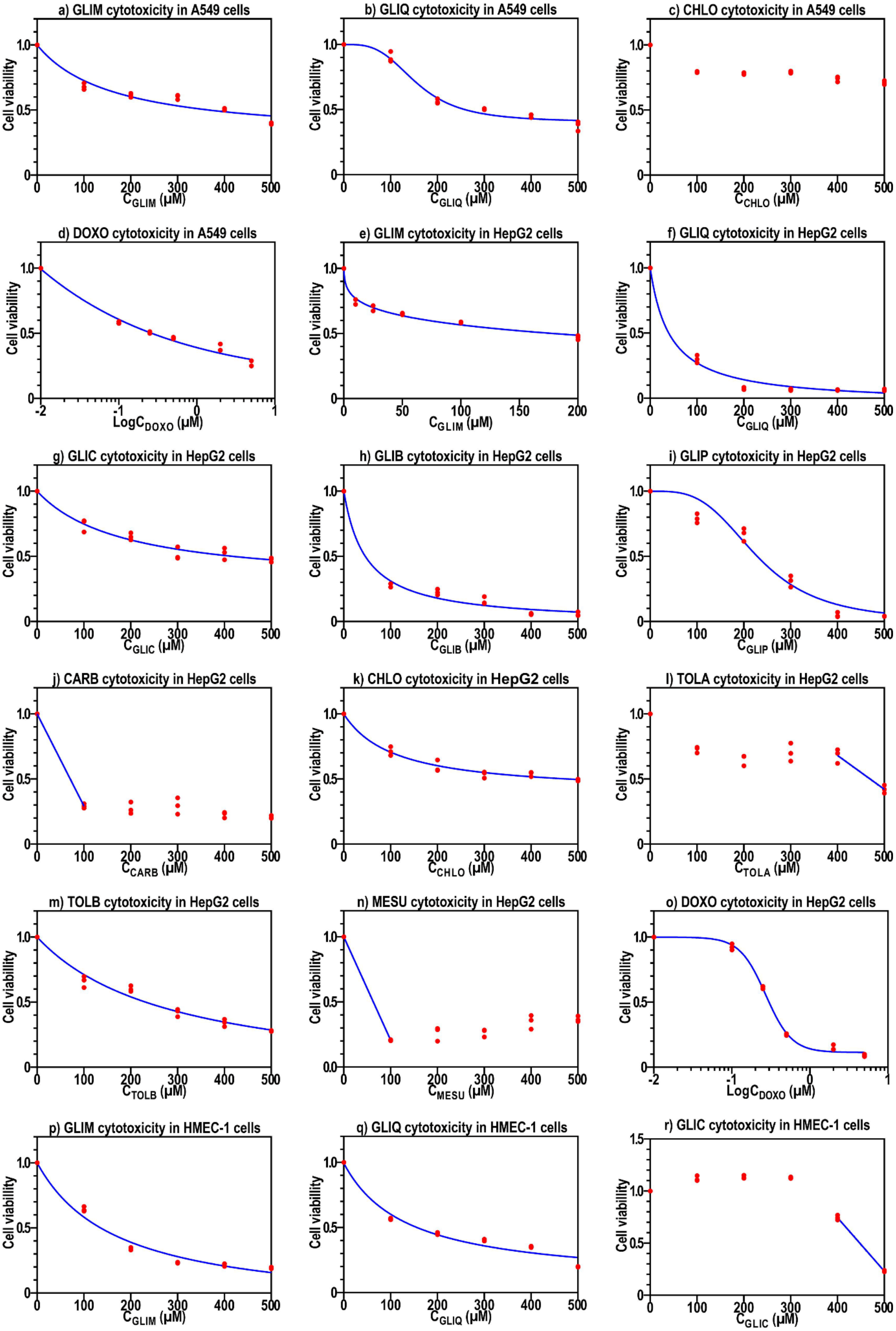

Figure S8 cont. Viability charts and curves for IC_{50} calculation

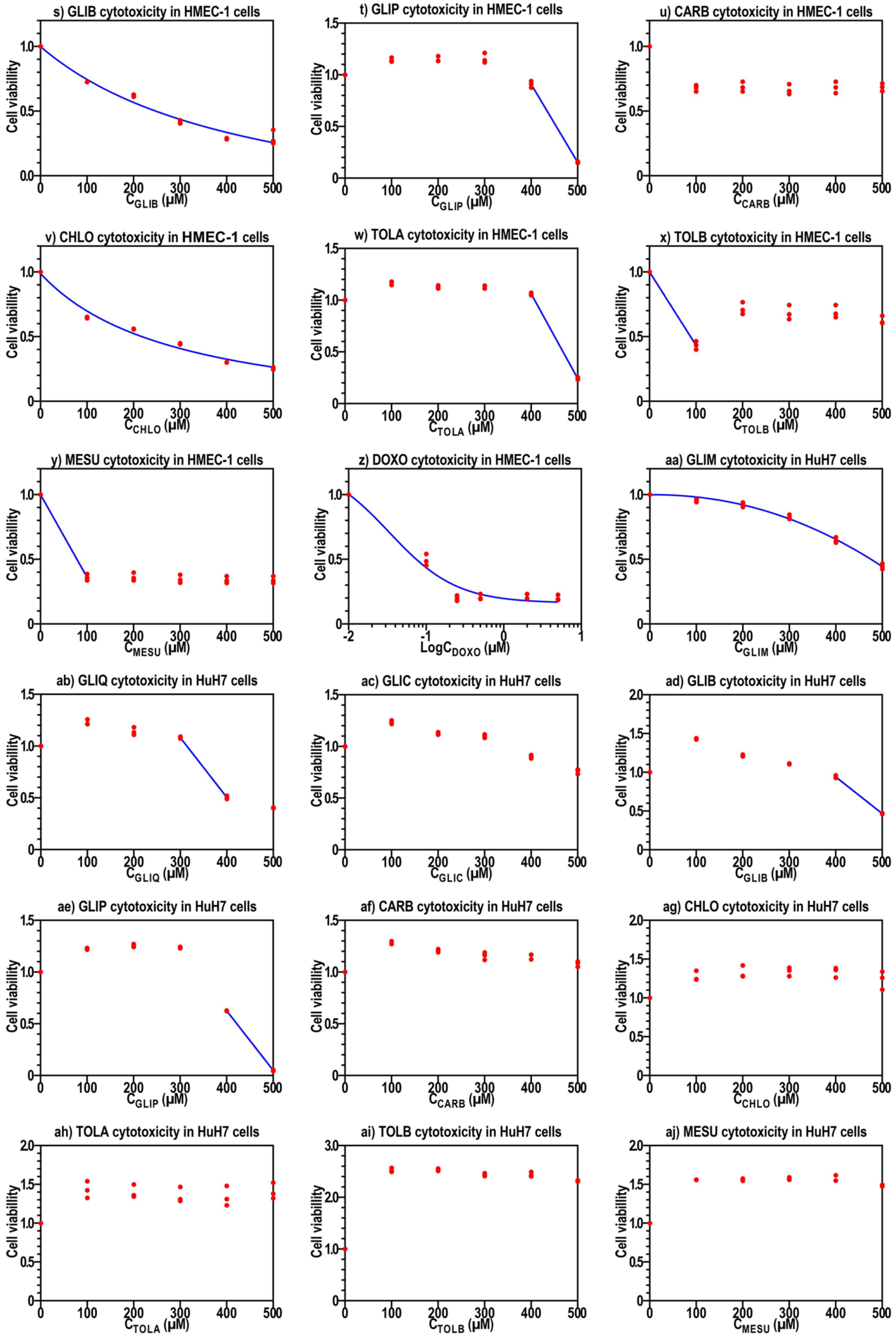

Figure S8 cont. Viability charts and curves for IC_{50} calculation

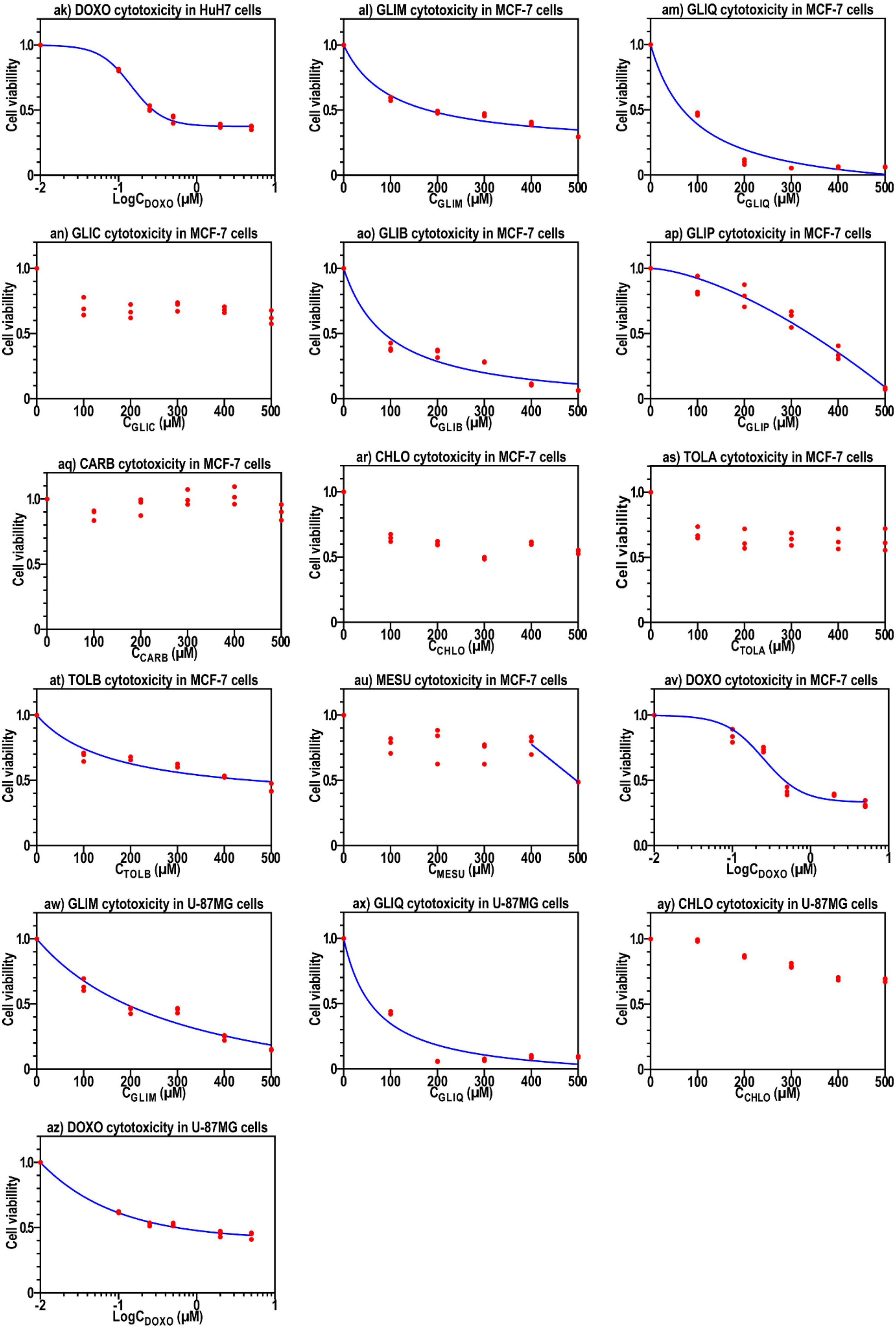

Figure S9. Cytotoxicity of DOXO+SUs combinations on MCF-7 cell line

GLIM (μM)	MCF-7 cell viability			GLIQ (μM)	MCF-7 cell viability		
DOXO (μM)	***0.225***	***0.450***	***0.900***	***DOXO (μM)***	***0.225***	***0.450***	***0.900***
**0**	0.769 ± 0.012	0.493 ± 0.006	0.259 ± 0.015	**0**	0.760 ± 0.005	0.491 ± 0.013	0.273 ± 0.002
**100**	0.130 ± 0.023	0.097 ± 0.065	0.146 ± 0.025	**100**	0.319 ± 0.027	0.393 ± 0.010	0.317 ± 0.023
**200**	0.151 ± 0.023	0.126 ± 0.010	0.089 ± 0.070	**200**	0.058 ± 0.001	0.064 ± 0.002	0.057 ± 0.003
**300**	0.138 ± 0.030	0.128 ± 0.024	0.124 ± 0.028	**300**	0.052 ± 0.001	0.055 ± 0.001	0.050 ± 0.000
**400**	0.119 ± 0.029	0.113 ± 0.034	0.112 ± 0.023	**400**	0.053 ± 0.001	0.057 ± 0.002	0.051 ± 0.001
**500**	0.098 ± 0.029	0.105 ± 0.027	0.105 ± 0.015	**500**	0.056 ± 0.001	0.060 ± 0.002	0.054 ± 0.000
**GLIC (μM)**	**MCF-7 cell viability**			**GLIB (μM)**	**MCF-7 cell viability**		
DOXO (μM)	***0.225***	***0.450***	***0.900***	***DOXO (μM)***	***0.225***	***0.450***	***0.900***
**0**	0.742 ± 0.001	0.495 ± 0.031	0.291 ± 0.020	**0**	0.762 ± 0.006	0.514 ± 0.007	0.284 ± 0.001
**100**	0.508 ± 0.052	0.404 ± 0.036	0.286 ± 0.021	**100**	0.320 ± 0.035	0.336 ± 0.033	0.296 ± 0.029
**200**	0.548 ± 0.068	0.472 ± 0.018	0.257 ± 0.022	**200**	0.207 ± 0.019	0.188 ± 0.036	0.210 ± 0.028
**300**	0.547 ± 0.040	0.486 ± 0.044	0.275 ± 0.009	**300**	0.132 ± 0.002	0.108 ± 0.016	0.114 ± 0.010
**400**	0.520 ± 0.066	0.414 ± 0.027	0.245 ± 0.022	**400**	0.077 ± 0.007	0.074 ± 0.001	0.076 ± 0.010
**500**	0.396 ± 0.036	0.414 ± 0.027	0.191 ± 0.004	**500**	0.073 ± 0.002	0.066 ± 0.003	0.069 ± 0.002
**GLIP (μM)**	**MCF-7 cell viability**			**CARB (μM)**	**MCF-7 cell viability**		
DOXO (μM)	***0.225***	***0.450***	***0.900***	***DOXO (μM)***	***0.225***	***0.450***	***0.900***
**0**	0.760 ± 0.005	0.491 ± 0.013	0.273 ± 0.002	**0**	0.752 ± 0.014	0.502 ± 0.019	0.285 ± 0.018
**100**	0.213 ± 0.049	0.283 ± 0.021	0.277 ± 0.021	**100**	0.237 ± 0.033	0.294 ± 0.023	0.248 ± 0.044
**200**	0.238 ± 0.035	0.332 ± 0.010	0.277 ± 0.017	**200**	0.230 ± 0.032	0.284 ± 0.034	0.241 ± 0.044
**300**	0.223 ± 0.033	0.273 ± 0.021	0.223 ± 0.008	**300**	0.296 ± 0.031	0.294 ± 0.037	0.243 ± 0.034
**400**	0.121 ± 0.007	0.151 ± 0.039	0.116 ± 0.006	**400**	0.313 ± 0.008	0.291 ± 0.014	0.221 ± 0.031
**500**	0.080 ± 0.006	0.082 ± 0.008	0.080 ± 0.006	**500**	0.376 ± 0.032	0.272 ± 0.019	0.254 ± 0.023
**CHLO (μM)**	**MCF-7 cell viability**			**TOLA (μM)**	**MCF-7 cell viability**		
DOXO (μM)	***0.225***	***0.450***	***0.900***	***DOXO (μM)***	***0.225***	***0.450***	***0.900***
**0**	0.771 ± 0.007	0.502 ± 0.004	0.303 ± 0.011	**0**	0.769 ± 0.005	0.520 ± 0.018	0.282 ± 0.014
**100**	0.294 ± 0.012	0.330 ± 0.053	0.139 ± 0.008	**100**	0.452 ± 0.042	0.357 ± 0.036	0.260 ± 0.035
**200**	0.305 ± 0.011	0.331 ± 0.091	0.132 ± 0.012	**200**	0.433 ± 0.044	0.379 ± 0.032	0.267 ± 0.028
**300**	0.281 ± 0.015	0.320 ± 0.073	0.128 ± 0.014	**300**	0.399 ± 0.043	0.400 ± 0.032	0.268 ± 0.036
**400**	0.363 ± 0.039	0.366 ± 0.063	0.128 ± 0.010	**400**	0.409 ± 0.061	0.396 ± 0.041	0.252 ± 0.023
**500**	0.324 ± 0.028	0.242 ± 0.036	0.122 ± 0.012	**500**	0.286 ± 0.027	0.353 ± 0.043	0.166 ± 0.009
**TOLB (μM)**	**MCF-7 cell viability**			**MESU (μM)**	**MCF-7 cell viability**		
DOXO (μM)	***0.225***	***0.450***	***0.900***	***DOXO (μM)***	***0.225***	***0.450***	***0.900***
**0**	0.737 ± 0.015	0.509 ± 0.006	0.279 ± 0.010	**0**	0.755 ± 0.006	0.523 ± 0.008	0.283 ± 0.004
**100**	0.469 ± 0.009	0.382 ± 0.020	0.217 ± 0.028	**100**	0.268 ± 0.045	0.243 ± 0.018	0.272 ± 0.020
**200**	0.404 ± 0.018	0.258 ± 0.016	0.202 ± 0.022	**200**	0.261 ± 0.048	0.216 ± 0.006	0.242 ± 0.015
**300**	0.395 ± 0.037	0.223 ± 0.022	0.188 ± 0.033	**300**	0.255 ± 0.035	0.222 ± 0.010	0.249 ± 0.013
**400**	0.303 ± 0.014	0.182 ± 0.011	0.178 ± 0.025	**400**	0.262 ± 0.019	0.224 ± 0.013	0.234 ± 0.017
**500**	0.211 ± 0.007	0.154 ± 0.010	0.151 ± 0.025	**500**	0.277 ± 0.033	0.232 ± 0.014	0.222 ± 0.012

Figure S10. Synergistic effects of DOXO+SUs combinations on MCF-7 cell line

GLIM (µM)	MCF-7 Combination Index			GLIQ (µM)	MCF-7 Combination Index		
DOXO (µM)	***0.225***	***0.450***	***0.900***	***DOXO (µM)***	***0.225***	***0.450***	***0.900***
**100**	0.044 ± 0.013	0.039 ± 0.039	0.098 ± 0.039	**100**	0.957 ± 0.041	1.319 ± 0.085	1.153 ± 0.087
**200**	0.099 ± 0.023	0.083 ± 0.016	0.076 ± 0.066	**200**	0.515 ± 0.022	0.551 ± 0.007	0.519 ± 0.035
**300**	0.122 ± 0.037	0.118 ± 0.031	0.132 ± 0.040	**300**	0.717 ± 0.031	0.749 ± 0.019	0.707 ± 0.023
**400**	0.122 ± 0.043	0.123 ± 0.053	0.136 ± 0.040	**400**	0.971 ± 0.028	1.016 ± 0.017	0.947 ± 0.025
**500**	0.109 ± 0.045	0.131 ± 0.045	0.142 ± 0.024	**500**	1.261 ± 0.048	1.316 ± 0.085	1.231 ± 0.041
**GLIC (µM)**	**MCF-7 Combination Index**			**GLIB (µM)**	**MCF-7 Combination Index**		
DOXO (µM)	***0.225***	***0.450***	***0.900***	***DOXO (µM)***	***0.225***	***0.450***	***0.900***
**100**	0.259 ± 0.092	0.253 ± 0.083	0.211 ± 0.049	**100**	0.670 ± 0.109	0.784 ± 0.075	0.789 ± 0.147
**200**	0.378 ± 0.200	0.398 ± 0.069	0.172 ± 0.070	**200**	0.797 ± 0.066	0.749 ± 0.119	0.890 ± 0.136
**300**	0.368 ± 0.132	0.451 ± 0.153	0.196 ± 0.058	**300**	0.791 ± 0.024	0.676 ± 0.080	0.727 ± 0.072
**400**	0.344 ± 0.215	0.276 ± 0.080	0.156 ± 0.064	**400**	0.677 ± 0.053	0.658 ± 0.021	0.684 ± 0.088
**500**	0.131 ± 0.055	0.280 ± 0.085	0.091 ± 0.027	**500**	0.803 ± 0.019	0.744 ± 0.008	0.785 ± 0.049
**GLIP (µM)**	**MCF-7 Combination Index**			**CARB (µM)**	**MCF-7 Combination Index**		
DOXO (µM)	***0.225***	***0.450***	***0.900***	***DOXO (µM)***	***0.225***	***0.450***	***0.900***
**100**	0.244 ± 0.029	0.354 ± 0.033	0.445 ± 0.046	**100**	0.035 ± 0.007	0.113 ± 0.010	0.155 ± 0.026
**200**	0.490 ± 0.040	0.677 ± 0.024	0.690 ± 0.031	**200**	0.034 ± 0.001	0.106 ± 0.005	0.148 ± 0.019
**300**	0.689 ± 0.061	0.823 ± 0.035	0.777 ± 0.037	**300**	0.059 ± 0.021	0.114 ± 0.018	0.149 ± 0.007
**400**	0.651 ± 0.084	0.732 ± 0.131	0.664 ± 0.077	**400**	0.068 ± 0.013	0.111 ± 0.022	0.122 ± 0.010
**500**	0.664 ± 0.093	0.677 ± 0.105	0.678 ± 0.097	**500**	0.108 ± 0.013	0.096 ± 0.016	0.164 ± 0.014
**CHLO (µM)**	**MCF-7 Combination Index**			**TOLA (µM)**	**MCF-7 Combination Index**		
DOXO (µM)	***0.225***	***0.450***	***0.900***	***DOXO (µM)***	***0.225***	***0.450***	***0.900***
**100**	0.061 ± 0.015	0.159 ± 0.044	0.051 ± 0.022	**100**	0.185 ± 0.035	0.183 ± 0.022	0.172 ± 0.009
**200**	0.071 ± 0.015	0.204 ± 0.151	0.047 ± 0.021	**200**	0.164 ± 0.027	0.217 ± 0.017	0.183 ± 0.030
**300**	0.062 ± 0.005	0.182 ± 0.109	0.044 ± 0.019	**300**	0.131 ± 0.017	0.254 ± 0.021	0.189 ± 0.065
**400**	0.166 ± 0.086	0.296 ± 0.190	0.044 ± 0.017	**400**	0.146 ± 0.040	0.251 ± 0.033	0.161 ± 0.022
**500**	0.116 ± 0.048	0.086 ± 0.017	0.039 ± 0.012	**500**	0.054 ± 0.004	0.183 ± 0.024	0.070 ± 0.023
**TOLB (µM)**	**MCF-7 Combination Index**			**MESU (µM)**	**MCF-7 Combination Index**		
DOXO (µM)	***0.225***	***0.450***	***0.900***	***DOXO (µM)***	***0.225***	***0.450***	***0.900***
**100**	0.343 ± 0.019	0.294 ± 0.056	0.144 ± 0.074	**100**	0.056 ± 0.026	0.084 ± 0.034	0.198 ± 0.025
**200**	0.318 ± 0.059	0.147 ± 0.072	0.141 ± 0.069	**200**	0.062 ± 0.032	0.069 ± 0.020	0.159 ± 0.036
**300**	0.394 ± 0.134	0.128 ± 0.048	0.141 ± 0.091	**300**	0.065 ± 0.026	0.078 ± 0.021	0.178 ± 0.034
**400**	0.240 ± 0.070	0.098 ± 0.034	0.139 ± 0.079	**400**	0.075 ± 0.018	0.085 ± 0.029	0.163 ± 0.044
**500**	0.137 ± 0.040	0.082 ± 0.036	0.113 ± 0.075	**500**	0.102 ± 0.066	0.097 ± 0.027	0.151 ± 0.045

Figure S11. Median-effect plots of DOXO+SUs combinations on MCF-7 cell line

DOXO+GLIM in MCF-7 cells

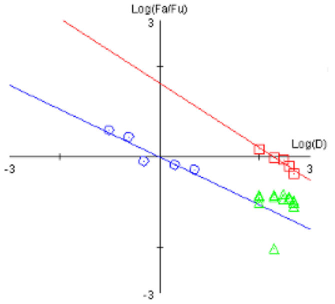

DOXO	GLIM	Effect	CI
0.225	100.0	0.11681	0.03492
0.225	200.0	0.12760	0.07517
0.225	300.0	0.10756	0.08333
0.225	400.0	0.10717	0.10914
0.225	500.0	0.08866	0.10058
0.450	100.0	0.08905	0.02512
0.450	200.0	0.11604	0.06907
0.450	300.0	0.13223	0.12274
0.450	400.0	0.11604	0.12866
0.450	500.0	0.07672	0.08246
0.900	100.0	0.12529	0.05603
0.900	200.0	0.00951	0.00166
0.900	300.0	0.13030	0.13194
0.900	400.0	0.10601	0.11899
0.900	500.0	0.09908	0.12978

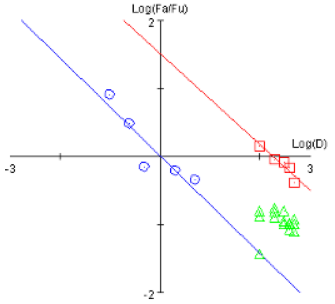

DOXO	GLIM	Effect	CI
0.225	100.0	0.11692	0.03936
0.225	200.0	0.15146	0.10201
0.225	300.0	0.13950	0.12420
0.225	400.0	0.09699	0.08690
0.225	500.0	0.07440	0.06911
0.450	100.0	0.03587	0.00842
0.450	200.0	0.13508	0.10014
0.450	300.0	0.10230	0.08433
0.450	400.0	0.07750	0.06731
0.450	500.0	0.10850	0.13938
0.900	100.0	0.13995	0.10508
0.900	200.0	0.11382	0.10021
0.900	300.0	0.09389	0.09183
0.900	400.0	0.09300	0.10845
0.900	500.0	0.09345	0.12744

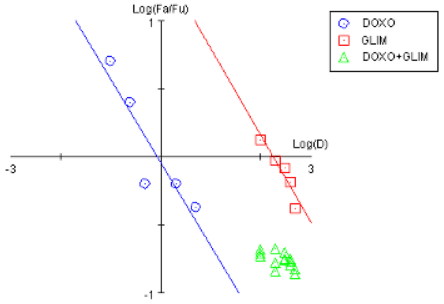

DOX	GLIM	Effect	CI
0.225	100.0	0.15747	0.05848
0.225	200.0	0.17438	0.12084
0.225	300.0	0.16682	0.15706
0.225	400.0	0.15169	0.17010
0.225	500.0	0.12989	0.15735
0.450	100.0	0.16504	0.08272
0.450	200.0	0.12678	0.07852
0.450	300.0	0.15080	0.14600
0.450	400.0	0.14591	0.17325
0.450	500.0	0.13078	0.17139
0.900	100.0	0.17393	0.13322
0.900	200.0	0.14368	0.12754
0.900	300.0	0.14858	0.17282
0.900	400.0	0.13746	0.18191
0.900	500.0	0.12100	0.17002

DOXO+GLIQ in MCF-7 cells

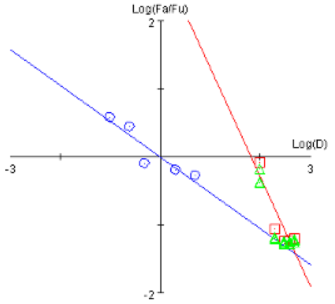

DOXO	GLIQ	Effect	CI
0.225	100.0	0.29392	0.91553
0.225	200.0	0.05873	0.53799
0.225	300.0	0.05247	0.74858
0.225	400.0	0.05273	1.00106
0.225	500.0	0.05664	1.31151
0.450	100.0	0.40407	1.39654
0.450	200.0	0.06160	0.55660
0.450	300.0	0.05456	0.76905
0.450	400.0	0.05534	1.03443
0.450	500.0	0.05847	1.34045
0.900	100.0	0.30384	1.08468
0.900	200.0	0.06082	0.55434
0.900	300.0	0.05038	0.73141
0.900	400.0	0.05038	0.97408
0.900	500.0	0.05403	1.27429

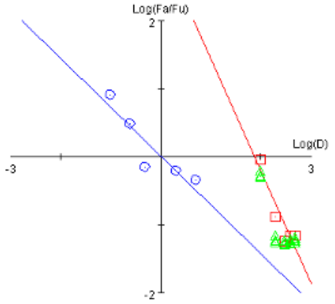

DOXO	GLIQ	Effect	CI
0.225	100.0	0.34734	0.99672
0.225	200.0	0.05759	0.49331
0.225	300.0	0.05162	0.68743
0.225	400.0	0.05422	0.94470
0.225	500.0	0.05681	1.21587
0.450	100.0	0.38573	1.22874
0.450	200.0	0.06563	0.54266
0.450	300.0	0.05629	0.73121
0.450	400.0	0.05888	1.00094
0.450	500.0	0.06226	1.29510
0.900	100.0	0.34397	1.25093
0.900	200.0	0.05396	0.48499
0.900	300.0	0.05006	0.68483
0.900	400.0	0.05136	0.92359
0.900	500.0	0.05422	1.19203

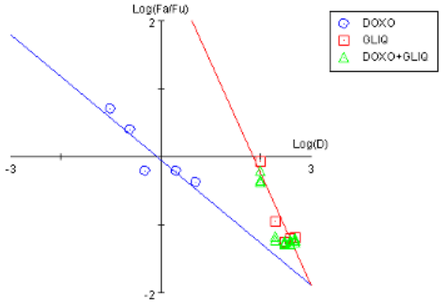

DOXO	GLIQ	Effect	CI
0.225	100.0	0.31659	0.95970
0.225	200.0	0.05741	0.51236
0.225	300.0	0.05137	0.71349
0.225	400.0	0.05279	0.96755
0.225	500.0	0.05600	1.25618
0.450	100.0	0.38980	1.33074
0.450	200.0	0.06409	0.55429
0.450	300.0	0.05471	0.74593
0.450	400.0	0.05638	1.01262
0.450	500.0	0.05959	1.31120
0.900	100.0	0.30311	1.12247
0.900	200.0	0.05690	0.51759
0.900	300.0	0.04958	0.70374
0.900	400.0	0.05022	0.94333
0.900	500.0	0.05343	1.22571

DOXO+GLIC in MCF-7 cells

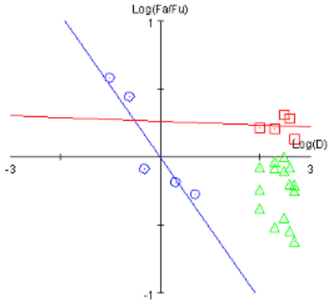

DOXO	GLIC	Effect	CI
0.225	100.0	0.45843	0.16313
0.225	200.0	0.48271	0.19630
0.225	300.0	0.50183	0.22699
0.225	400.0	0.45757	0.16206
0.225	500.0	0.36198	0.07630
0.450	100.0	0.36463	0.15595
0.450	200.0	0.45500	0.31779
0.450	300.0	0.44382	0.29168
0.450	400.0	0.38819	0.18871
0.450	500.0	0.38625	0.18581
0.900	100.0	0.29603	0.17280
0.900	200.0	0.23373	0.09391
0.900	300.0	0.26552	0.12968
0.900	400.0	0.22385	0.08444
0.900	500.0	0.19527	0.06083

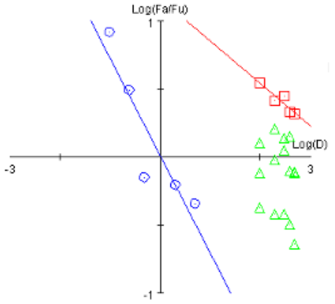

DOXO	GLIC	Effect	CI
0.225	100.0	0.56250	0.34668
0.225	200.0	0.61850	0.59259
0.225	300.0	0.57947	0.48802
0.225	400.0	0.58852	0.58172
0.225	500.0	0.43382	0.18708
0.450	100.0	0.43496	0.30811
0.450	200.0	0.49067	0.44206
0.450	300.0	0.53167	0.59627
0.450	400.0	0.44146	0.34512
0.450	500.0	0.44005	0.35024
0.900	100.0	0.29977	0.26690
0.900	200.0	0.27715	0.22958
0.900	300.0	0.27630	0.22902
0.900	400.0	0.24236	0.17902
0.900	500.0	0.18637	0.11214

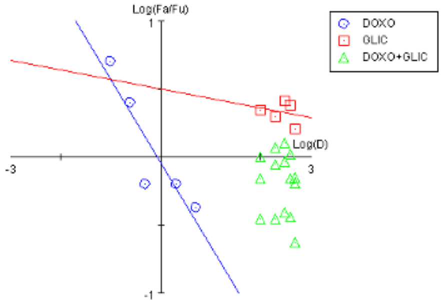

DOXO	GLIC	Effect	CI
0.225	100.0	0.50336	0.26839
0.225	200.0	0.54133	0.34387
0.225	300.0	0.55976	0.38865
0.225	400.0	0.51410	0.28786
0.225	500.0	0.39299	0.12956
0.450	100.0	0.41204	0.29471
0.450	200.0	0.47039	0.43314
0.450	300.0	0.48175	0.46640
0.450	400.0	0.41118	0.29302
0.450	500.0	0.41717	0.30500
0.900	100.0	0.26102	0.19361
0.900	200.0	0.26102	0.19361
0.900	300.0	0.28238	0.23076
0.900	400.0	0.26847	0.20603
0.900	500.0	0.19143	0.10107

Figure S11 cont. Median-effect plots of DOXO+SUs combinations on MCF-7 cell line

DOXO+GLIB in MCF-7 cells

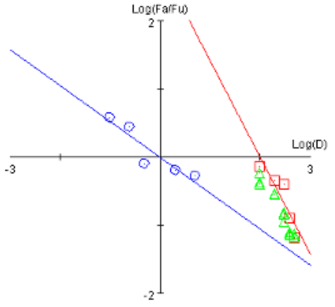

DOXO	GLIB	Effect	CI
0.225	100.0	0.29931	0.58907
0.225	200.0	0.22700	0.85817
0.225	300.0	0.13369	0.80078
0.225	400.0	0.07013	0.63952
0.225	500.0	0.07044	0.80168
0.450	100.0	0.36850	0.84015
0.450	200.0	0.22887	0.88613
0.450	300.0	0.12617	0.76913
0.450	400.0	0.07389	0.66748
0.450	500.0	0.06450	0.75103
0.900	100.0	0.28554	0.67638
0.900	200.0	0.22198	0.90108
0.900	300.0	0.10269	0.65736
0.900	400.0	0.06731	0.62434
0.900	500.0	0.06888	0.79286

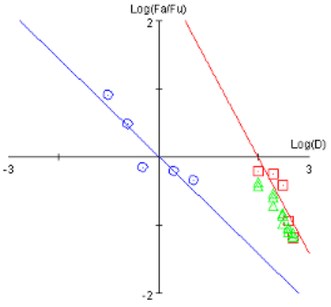

DOXO	GLIB	Effect	CI
0.225	100.0	0.30081	0.62766
0.225	200.0	0.20421	0.80417
0.225	300.0	0.13193	0.80787
0.225	400.0	0.08363	0.73789
0.225	500.0	0.07261	0.82311
0.450	100.0	0.30243	0.69912
0.450	200.0	0.16175	0.67158
0.450	300.0	0.09530	0.62643
0.450	400.0	0.07358	0.67305
0.450	500.0	0.06353	0.74660
0.900	100.0	0.27455	0.73494
0.900	200.0	0.22950	1.01993
0.900	300.0	0.12253	0.80099
0.900	400.0	0.08687	0.78526
0.900	500.0	0.07131	0.82978

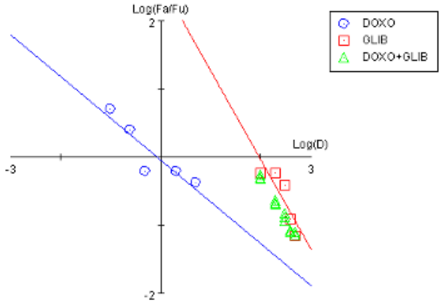

DOXO	GLIB	Effect	CI
0.225	100.0	0.36008	0.79335
0.225	200.0	0.18896	0.72750
0.225	300.0	0.13032	0.76408
0.225	400.0	0.07819	0.65344
0.225	500.0	0.07476	0.78565
0.450	100.0	0.33711	0.81379
0.450	200.0	0.17284	0.68794
0.450	300.0	0.10391	0.63390
0.450	400.0	0.07476	0.63380
0.450	500.0	0.06859	0.73526
0.900	100.0	0.32900	0.95518
0.900	200.0	0.17730	0.74891
0.900	300.0	0.11660	0.72156
0.900	400.0	0.07476	0.64261
0.900	500.0	0.06756	0.73346

DOXO+GLIP in MCF-7 cells

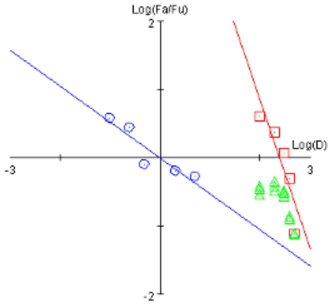

DOXO	GLIP	Effect	CI
0.225	100.0	0.22563	0.25521
0.225	200.0	0.25155	0.52738
0.225	300.0	0.24169	0.75617
0.225	400.0	0.12377	0.67504
0.225	500.0	0.07265	0.63971
0.450	100.0	0.25995	0.31610
0.450	200.0	0.31435	0.67774
0.450	300.0	0.24936	0.79994
0.450	400.0	0.11537	0.65477
0.450	500.0	0.07448	0.64961
0.900	100.0	0.28185	0.41964
0.900	200.0	0.28185	0.68770
0.900	300.0	0.21468	0.75703
0.900	400.0	0.11281	0.65576
0.900	500.0	0.07265	0.64504

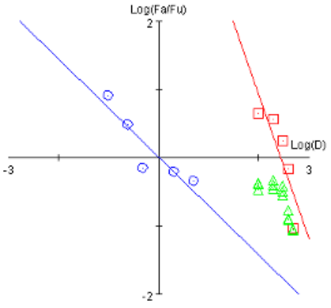

DOXO	GLIP	Effect	CI
0.225	100.0	0.25564	0.26601
0.225	200.0	0.26420	0.49548
0.225	300.0	0.24241	0.67418
0.225	400.0	0.11362	0.55696
0.225	500.0	0.08327	0.58592
0.450	100.0	0.30000	0.37418
0.450	200.0	0.31829	0.65210
0.450	300.0	0.27938	0.80556
0.450	400.0	0.14475	0.65770
0.450	500.0	0.08171	0.58756
0.900	100.0	0.29611	0.49834
0.900	200.0	0.29066	0.72212
0.900	300.0	0.22412	0.75383
0.900	400.0	0.11245	0.59042
0.900	500.0	0.08132	0.60088

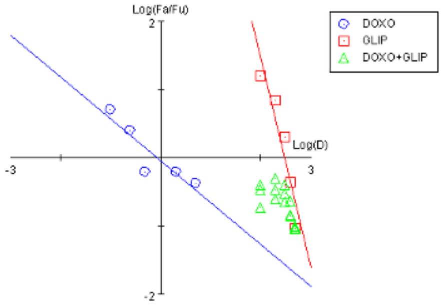

DOXO	GLIP	Effect	CI
0.225	100.0	0.15917	0.21069
0.225	200.0	0.19845	0.44862
0.225	300.0	0.18412	0.63680
0.225	400.0	0.12684	0.72023
0.225	500.0	0.08347	0.76663
0.450	100.0	0.29051	0.37123
0.450	200.0	0.33347	0.70015
0.450	300.0	0.28887	0.86403
0.450	400.0	0.19190	0.88234
0.450	500.0	0.08961	0.79289
0.900	100.0	0.25409	0.41629
0.900	200.0	0.25818	0.65980
0.900	300.0	0.23036	0.82032
0.900	400.0	0.12316	0.74437
0.900	500.0	0.08470	0.78720

DOXO+CARB in MCF-7 cells*

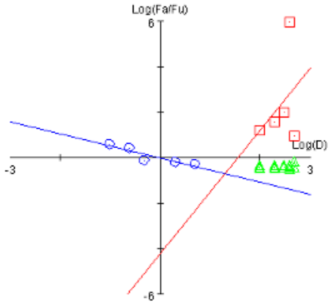

DOXO	CARB	Effect	CI
0.225	100.0	0.26888	4.07695
0.225	200.0	0.26683	8.15084
0.225	300.0	0.28654	11.7842
0.225	400.0	0.31897	14.8534
0.225	500.0	0.41174	16.0780
0.450	100.0	0.31938	3.80394
0.450	200.0	0.32266	7.46382
0.450	300.0	0.32841	11.0410
0.450	400.0	0.29803	15.4314
0.450	500.0	0.28900	19.5693
0.900	100.0	0.29721	4.01606
0.900	200.0	0.28859	7.96242
0.900	300.0	0.27997	12.0338
0.900	400.0	0.25369	16.7633
0.900	500.0	0.27956	19.9713

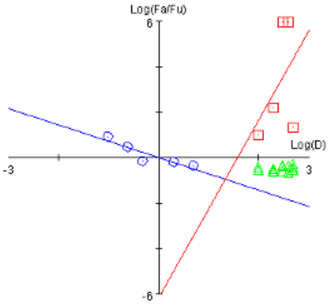

DOXO	CARB	Effect	CI
0.225	100.0	0.23815	3.64576
0.225	200.0	0.21031	7.53997
0.225	300.0	0.33042	9.70362
0.225	400.0	0.31712	13.1046
0.225	500.0	0.35453	15.7004
0.450	100.0	0.28969	3.49122
0.450	200.0	0.25894	7.10449
0.450	300.0	0.29925	10.1158
0.450	400.0	0.29967	13.4372
0.450	500.0	0.27515	17.2578
0.900	100.0	0.23358	3.79184
0.900	200.0	0.20033	7.74956
0.900	300.0	0.21446	11.3297
0.900	400.0	0.19160	15.5802
0.900	500.0	0.23525	18.2558

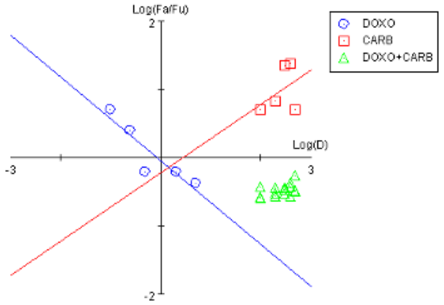

DOXO	CARB	Effect	CI
0.225	100.0	0.20370	562.111
0.225	200.0	0.21426	989.871
0.225	300.0	0.27046	805.394
0.225	400.0	0.30366	777.174
0.225	500.0	0.36024	583.944
0.450	100.0	0.27310	261.516
0.450	200.0	0.27122	532.900
0.450	300.0	0.25500	944.223
0.450	400.0	0.27386	1037.81
0.450	500.0	0.25236	1617.96
0.900	100.0	0.21237	506.272
0.900	200.0	0.23501	780.807
0.900	300.0	0.23425	1181.04
0.900	400.0	0.21690	1919.20
0.900	500.0	0.24632	1725.44

* – A commentary on these results is provided in Figure S12.

Figure S11 cont. Median-effect plots of DOXO+SUs combinations on MCF-7 cell line

DOXO+CHLO in MCF-7 cells

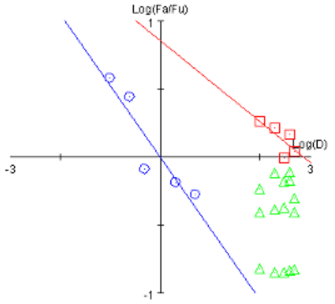

DOXO	CHLO	Effect	CI
0.225	100.0	0.28219	0.04372
0.225	200.0	0.29205	0.05507
0.225	300.0	0.29791	0.06606
0.225	400.0	0.40511	0.25261
0.225	500.0	0.33457	0.12586
0.450	100.0	0.37184	0.18799
0.450	200.0	0.43407	0.37847
0.450	300.0	0.39926	0.30608
0.450	400.0	0.43900	0.51195
0.450	500.0	0.28250	0.10490
0.900	100.0	0.13278	0.02561
0.900	200.0	0.12569	0.02289
0.900	300.0	0.12446	0.02259
0.900	400.0	0.12847	0.02445
0.900	500.0	0.13001	0.02533

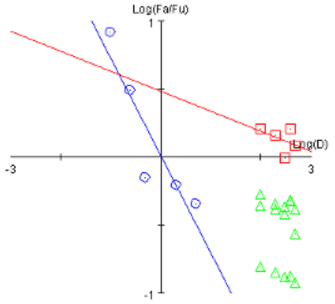

DOXO	CHLO	Effect	CI
0.225	100.0	0.30548	0.06916
0.225	200.0	0.31316	0.07297
0.225	300.0	0.27666	0.05700
0.225	400.0	0.32853	0.08146
0.225	500.0	0.29171	0.06352
0.450	100.0	0.34678	0.17985
0.450	200.0	0.29395	0.12806
0.450	300.0	0.30451	0.13760
0.450	400.0	0.32373	0.15646
0.450	500.0	0.21646	0.07229
0.900	100.0	0.13545	0.06567
0.900	200.0	0.12584	0.05838
0.900	300.0	0.11591	0.05126
0.900	400.0	0.11848	0.05306
0.900	500.0	0.10759	0.04562

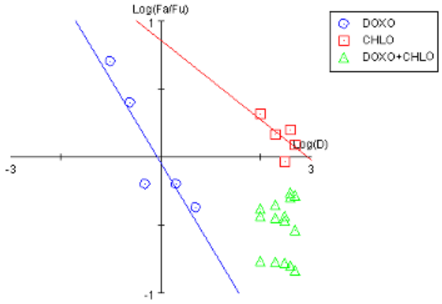

DOXO	CHLO	Effect	CI
0.225	100.0	0.29527	0.06988
0.225	200.0	0.30870	0.08576
0.225	300.0	0.26903	0.06332
0.225	400.0	0.35637	0.16259
0.225	500.0	0.34517	0.15841
0.450	100.0	0.27063	0.10884
0.450	200.0	0.26392	0.10625
0.450	300.0	0.25592	0.10189
0.450	400.0	0.33653	0.22035
0.450	500.0	0.22649	0.08022
0.900	100.0	0.14811	0.06150
0.900	200.0	0.14587	0.06001
0.900	300.0	0.14395	0.05877
0.900	400.0	0.13852	0.05482
0.900	500.0	0.12796	0.04729

DOXO+TOLA in MCF-7 cells

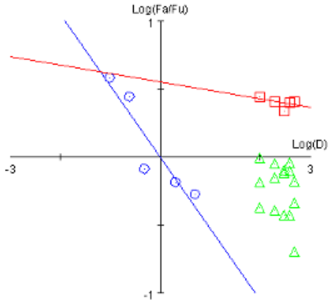

DOXO	TOLA	Effect	CI
0.225	100.0	0.49650	0.21799
0.225	200.0	0.47524	0.18545
0.225	300.0	0.44783	0.15040
0.225	400.0	0.47916	0.19107
0.225	500.0	0.31692	0.05207
0.450	100.0	0.39580	0.20051
0.450	200.0	0.41678	0.23653
0.450	300.0	0.43636	0.27537
0.450	400.0	0.44084	0.28505
0.450	500.0	0.40140	0.20961
0.900	100.0	0.29846	0.17667
0.900	200.0	0.28979	0.16320
0.900	300.0	0.27497	0.14203
0.900	400.0	0.27385	0.14052
0.900	500.0	0.16867	0.04330

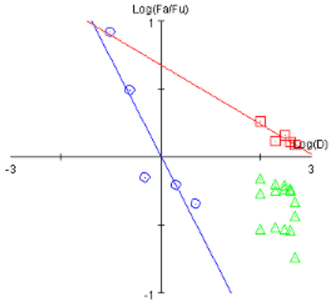

DOX	TOLA	Effect	CI
0.225	100.0	0.41347	0.14883
0.225	200.0	0.38803	0.13431
0.225	300.0	0.36858	0.12239
0.225	400.0	0.36858	0.12910
0.225	500.0	0.27082	0.05877
0.450	100.0	0.35162	0.18939
0.450	200.0	0.36110	0.20710
0.450	300.0	0.38504	0.25318
0.450	400.0	0.36135	0.21909
0.450	500.0	0.32095	0.16525
0.900	100.0	0.23017	0.16162
0.900	200.0	0.23491	0.16809
0.900	300.0	0.22868	0.16034
0.900	400.0	0.22743	0.15904
0.900	500.0	0.15636	0.08313

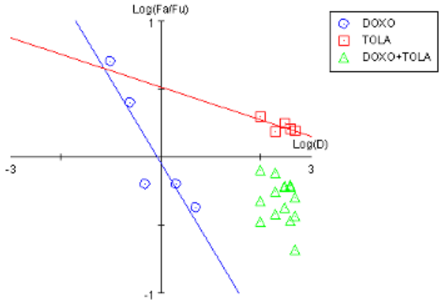

DOXO	TOLA	Effect	CI
0.225	100.0	0.44716	0.18684
0.225	200.0	0.43467	0.17239
0.225	300.0	0.38011	0.11886
0.225	400.0	0.37782	0.11705
0.225	500.0	0.27100	0.05260
0.450	100.0	0.32377	0.15871
0.450	200.0	0.36023	0.20661
0.450	300.0	0.37731	0.23291
0.450	400.0	0.38598	0.24740
0.450	500.0	0.33576	0.17340
0.900	100.0	0.25163	0.17872
0.900	200.0	0.27533	0.21798
0.900	300.0	0.29904	0.26314
0.900	400.0	0.25510	0.18413
0.900	500.0	0.17310	0.08275

DOXO+TOLB in MCF-7 cells

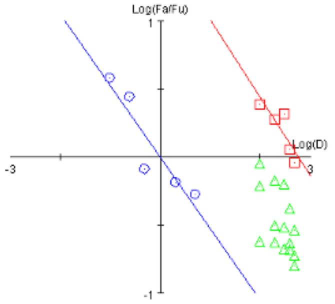

DOXO	TOLU	Effect	CI
0.225	100.0	0.47256	0.32239
0.225	200.0	0.40485	0.28187
0.225	300.0	0.38845	0.32643
0.225	400.0	0.29579	0.19356
0.225	500.0	0.22701	0.12270
0.450	100.0	0.37990	0.24914
0.450	200.0	0.24020	0.09612
0.450	300.0	0.23521	0.11327
0.450	400.0	0.19066	0.08371
0.450	500.0	0.15895	0.06572
0.900	100.0	0.19530	0.07532
0.900	200.0	0.19244	0.08680
0.900	300.0	0.17498	0.08143
0.900	400.0	0.17391	0.09163
0.900	500.0	0.13756	0.06225

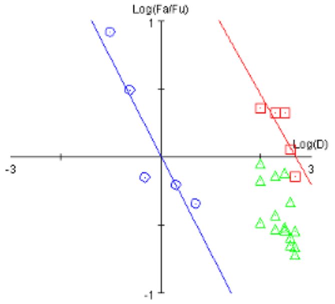

DOXO	TOLU	Effect	CI
0.225	100.0	0.47494	0.35820
0.225	200.0	0.42271	0.38602
0.225	300.0	0.43498	0.54864
0.225	400.0	0.31826	0.32095
0.225	500.0	0.22257	0.18287
0.450	100.0	0.40238	0.35745
0.450	200.0	0.27199	0.19725
0.450	300.0	0.23589	0.18197
0.450	400.0	0.18577	0.13713
0.450	500.0	0.16158	0.12261
0.900	100.0	0.24886	0.22234
0.900	200.0	0.22748	0.21845
0.900	300.0	0.22573	0.24516
0.900	400.0	0.20505	0.23010
0.900	500.0	0.17911	0.19949

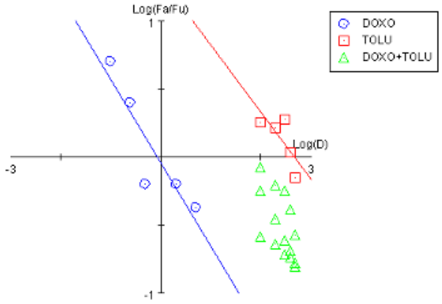

DOXO	TOLU	Effect	CI
0.225	100.0	0.45816	0.34923
0.225	200.0	0.38580	0.28701
0.225	300.0	0.36283	0.30648
0.225	400.0	0.29390	0.20559
0.225	500.0	0.21331	0.10630
0.450	100.0	0.36248	0.27672
0.450	200.0	0.26063	0.14736
0.450	300.0	0.19684	0.09004
0.450	400.0	0.17044	0.07428
0.450	500.0	0.14266	0.05635
0.900	100.0	0.20782	0.13340
0.900	200.0	0.18621	0.11685
0.900	300.0	0.16289	0.09643
0.900	400.0	0.15535	0.09435
0.900	500.0	0.13546	0.07635

Figure S11 cont. Median-effect plots of DOXO+SUs combinations on MCF-7 cell line

DOXO+MESU in MCF-7 cells

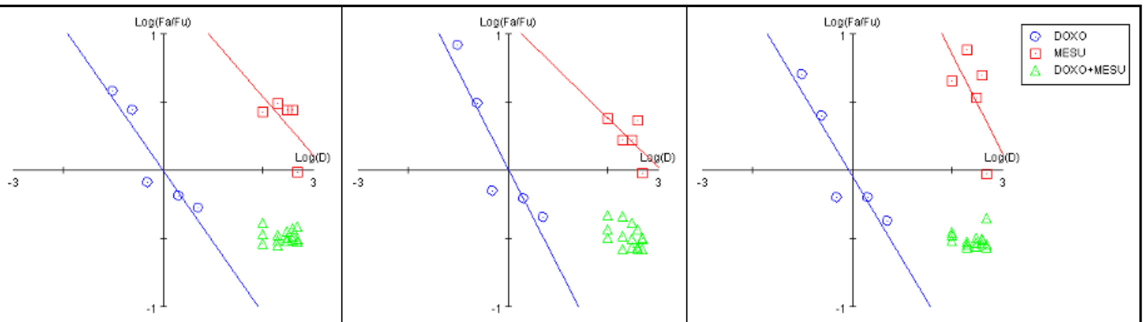

DOXO	MESU	Effect	CI
0.225	100.0	0.25378	0.03309
0.225	200.0	0.23546	0.03059
0.225	300.0	0.24223	0.03660
0.225	400.0	0.27410	0.05682
0.225	500.0	0.28008	0.06625
0.450	100.0	0.22510	0.04571
0.450	200.0	0.22191	0.04690
0.450	300.0	0.23386	0.05683
0.450	400.0	0.23665	0.06199
0.450	500.0	0.23904	0.06715
0.900	100.0	0.29243	0.17390
0.900	200.0	0.25179	0.12142
0.900	300.0	0.26375	0.14177
0.900	400.0	0.24861	0.12542
0.900	500.0	0.23307	0.10944

DOXO	MESU	Effect	CI
0.225	100.0	0.31850	0.08513
0.225	200.0	0.31656	0.09379
0.225	300.0	0.29450	0.08619
0.225	400.0	0.27090	0.07578
0.225	500.0	0.24303	0.06216
0.450	100.0	0.24342	0.09286
0.450	200.0	0.21091	0.07324
0.450	300.0	0.21633	0.07912
0.450	400.0	0.20975	0.07663
0.450	500.0	0.24033	0.10417
0.900	100.0	0.27128	0.22414
0.900	200.0	0.24884	0.19373
0.900	300.0	0.23916	0.18291
0.900	400.0	0.21556	0.15258
0.900	500.0	0.20898	0.14604

DOXO	MESU	Effect	CI
0.225	100.0	0.23311	0.05064
0.225	200.0	0.23194	0.06272
0.225	300.0	0.22804	0.07286
0.225	400.0	0.23975	0.09377
0.225	500.0	0.30847	0.17888
0.450	100.0	0.26045	0.11193
0.450	200.0	0.21515	0.08616
0.450	300.0	0.21671	0.09856
0.450	400.0	0.22413	0.11721
0.450	500.0	0.21632	0.12046
0.900	100.0	0.25264	0.19501
0.900	200.0	0.22413	0.16334
0.900	300.0	0.24444	0.20945
0.900	400.0	0.23810	0.21152
0.900	500.0	0.22374	0.19818

Figure S12. Commentary median-effect plots for the DOXO+CARB combination

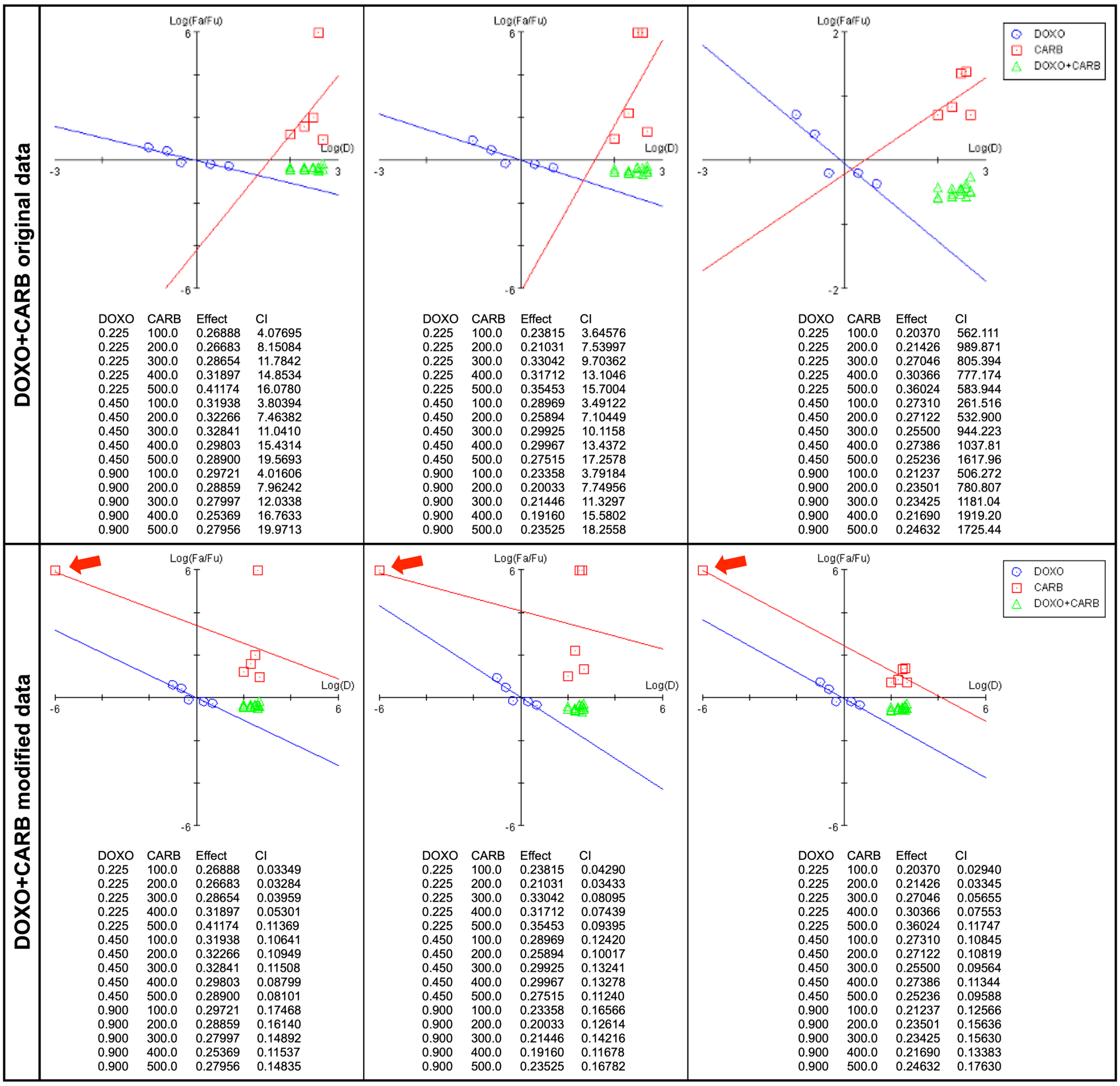

DOXO+CARB original data

DOXO	CARB	Effect	CI
0.225	100.0	0.26888	4.07695
0.225	200.0	0.26683	8.15084
0.225	300.0	0.28654	11.7842
0.225	400.0	0.31897	14.8534
0.225	500.0	0.41174	16.0780
0.450	100.0	0.31938	3.80394
0.450	200.0	0.32266	7.46382
0.450	300.0	0.32841	11.0410
0.450	400.0	0.29803	15.4314
0.450	500.0	0.28900	19.5693
0.900	100.0	0.29721	4.01606
0.900	200.0	0.28859	7.96242
0.900	300.0	0.27997	12.0338
0.900	400.0	0.25369	16.7633
0.900	500.0	0.27956	19.9713

DOXO	CARB	Effect	CI
0.225	100.0	0.23815	3.64576
0.225	200.0	0.21031	7.53997
0.225	300.0	0.33042	9.70362
0.225	400.0	0.31712	13.1046
0.225	500.0	0.35453	15.7004
0.450	100.0	0.28969	3.49122
0.450	200.0	0.25894	7.10449
0.450	300.0	0.29925	10.1158
0.450	400.0	0.29967	13.4372
0.450	500.0	0.27515	17.2578
0.900	100.0	0.23358	3.79184
0.900	200.0	0.20033	7.74956
0.900	300.0	0.21446	11.3297
0.900	400.0	0.19160	15.5802
0.900	500.0	0.23525	18.2558

DOXO	CARB	Effect	CI
0.225	100.0	0.20370	562.111
0.225	200.0	0.21426	989.871
0.225	300.0	0.27046	805.394
0.225	400.0	0.30366	777.174
0.225	500.0	0.36024	583.944
0.450	100.0	0.27310	261.516
0.450	200.0	0.27122	532.900
0.450	300.0	0.25500	944.223
0.450	400.0	0.27386	1037.81
0.450	500.0	0.25236	1617.96
0.900	100.0	0.21237	506.272
0.900	200.0	0.23501	780.807
0.900	300.0	0.23425	1181.04
0.900	400.0	0.21690	1919.20
0.900	500.0	0.24632	1725.44

DOXO+CARB modified data

DOXO	CARB	Effect	CI
0.225	100.0	0.26888	0.03349
0.225	200.0	0.26683	0.03284
0.225	300.0	0.28654	0.03959
0.225	400.0	0.31897	0.05301
0.225	500.0	0.41174	0.11369
0.450	100.0	0.31938	0.10641
0.450	200.0	0.32266	0.10949
0.450	300.0	0.32841	0.11508
0.450	400.0	0.29803	0.08799
0.450	500.0	0.28900	0.08101
0.900	100.0	0.29721	0.17468
0.900	200.0	0.28859	0.16140
0.900	300.0	0.27997	0.14892
0.900	400.0	0.25369	0.11537
0.900	500.0	0.27956	0.14835

DOXO	CARB	Effect	CI
0.225	100.0	0.23815	0.04290
0.225	200.0	0.21031	0.03433
0.225	300.0	0.33042	0.08095
0.225	400.0	0.31712	0.07439
0.225	500.0	0.35453	0.09395
0.450	100.0	0.28969	0.12420
0.450	200.0	0.25894	0.10017
0.450	300.0	0.29925	0.13241
0.450	400.0	0.29967	0.13278
0.450	500.0	0.27515	0.11240
0.900	100.0	0.23358	0.16566
0.900	200.0	0.20033	0.12614
0.900	300.0	0.21446	0.14216
0.900	400.0	0.19160	0.11678
0.900	500.0	0.23525	0.16782

DOXO	CARB	Effect	CI
0.225	100.0	0.20370	0.02940
0.225	200.0	0.21426	0.03345
0.225	300.0	0.27046	0.05655
0.225	400.0	0.30366	0.07553
0.225	500.0	0.36024	0.11747
0.450	100.0	0.27310	0.10845
0.450	200.0	0.27122	0.10819
0.450	300.0	0.25500	0.09564
0.450	400.0	0.27386	0.11344
0.450	500.0	0.25236	0.09588
0.900	100.0	0.21237	0.12566
0.900	200.0	0.23501	0.15636
0.900	300.0	0.23425	0.15630
0.900	400.0	0.21690	0.13383
0.900	500.0	0.24632	0.17630

In this particular case, we slightly adjusted the data. We introduced a 0 point (red arrow) that represents untreated MCF-7 cells and assigned them a survival rate of 1. This addition transformed the initially increasing trend line (original data) for the CARB effect into a decreasing one (modified data). This decreasing trend signifies a reduction in the survival rate of MCF-7 cells when treated with CARB. Consequently, we were able to attribute a cytotoxic effect to both drugs and compute the synergy of their cytotoxicity.

Figure S13. Cytotoxicity results for GLIM, GLIQ, and CHLO on various cell lines

**GLIM (µM)**	**A549 cell viability**			**GLIQ (µM)**	**A549 cell viability**		
DOXO (µM)	***0.073***	***0.145***	***0.290***	***DOXO (µM)***	**0.073**	***0.145***	***0.290***
**0**	0.631 ± 0.008	0.549 ± 0.005	0.502 ± 0.001	**0**	0.820 ± 0.004	0.655 ± 0.009	0.502 ± 0.004
**95.13**	0.559 ± 0.004	0.533 ± 0.004	0.240 ± 0.004	**81.30**	0.395 ± 0.005	0.301 ± 0.008	0.210 ± 0.005
**190.25**	0.474 ± 0.003	0.440 ± 0.001	0.224 ± 0.004	**162.60**	0.354 ± 0.016	0.306 ± 0.002	0.196 ± 0.001
**380.50**	0.428 ± 0.008	0.362 ± 0.001	0.121 ± 0.006	**325.20**	0.332 ± 0.006	0.255 ± 0.003	0.182 ± 0.002
**GLIM (µM)**	**HepG2 cell viability**			**GLIQ (µM)**	**HepG2 cell viability**		
DOXO (µM)	***0.180***	***0.360***	***0.720***	***DOXO (µM)***	***0.180***	***0.360***	***0.720***
**0**	0.801 ± 0.014	0.692 ± 0.016	0.501 ± 0.017	**0**	0.811 ± 0.010	0.701 ± 0.005	0.516 ± 0.004
**48.20**	0.638 ± 0.013	0.648 ± 0.019	0.375 ± 0.017	**8.48**	0.249 ± 0.006	0.100 ± 0.016	0.093 ± 0.016
**96.40**	0.616 ± 0.017	0.561 ± 0.034	0.244 ± 0.024	**16.95**	0.209 ± 0.004	0.099 ± 0.018	0.092 ± 0.010
**192.80**	0.395 ± 0.018	0.397 ± 0.026	0.220 ± 0.025	**33.90**	0.124 ± 0.011	0.094 ± 0.021	0.094 ± 0.013
**GLIM (µM)**	**HMEC-1 cell viability**			**GLIQ (µM)**	**HMEC-1 cell viability**		
DOXO (µM)	***0.020***	***0.040***	***0.080***	***DOXO (µM)***	***0.020***	***0.040***	***0.080***
**0**	0.870 ± 0.045	0.640 ± 0.013	0.525 ± 0.012	**0**	0.824 ± 0.034	0.602 ± 0.007	0.508 ± 0.007
**34.70**	0.522 ± 0.019	0.494 ± 0.030	0.357 ± 0.011	**38.78**	0.701 ± 0.004	0.651 ± 0.003	0.608 ± 0.001
**69.40**	0.483 ± 0.042	0.309 ± 0.001	0.292 ± 0.026	**77.55**	0.607 ± 0.011	0.555 ± 0.005	0.501 ± 0.001
**138.80**	0.235 ± 0.029	0.164 ± 0.010	0.131 ± 0.017	**155.10**	0.548 ± 0.001	0.449 ± 0.003	0.410 ± 0.003
**GLIM (µM)**	**HuH7 cell viability**			**GLIQ (µM)**	**HuH7 cell viability**		
DOXO (µM)	***0.163***	***0.325***	***0.650***	***DOXO (µM)***	***0.163***	***0.325***	***0.650***
**0**	0.963 ± 0.005	0.953 ± 0.009	0.535 ± 0.004	**0**	0.948 ± 0.007	0.959 ± 0.007	0.502 ± 0.013
**115.83**	0.947 ± 0.010	0.920 ± 0.017	0.860 ± 0.014	**106.10**	1.013 ± 0.004	1.020 ± 0.007	1.099 ± 0.005
**231.65**	0.804 ± 0.009	0.617 ± 0.010	0.549 ± 0.007	**212.20**	0.805 ± 0.012	0.898 ± 0.005	1.048 ± 0.006
**463.30**	0.510 ± 0.012	0.305 ± 0.001	0.203 ± 0.005	**424.40**	0.302 ± 0.004	0.396 ± 0.008	0.862 ± 0.006
**GLIM (µM)**	**MCF-7 cell viability**			**GLIQ (µM)**	**MCF-7 cell viability**		
DOXO (µM)	***0.113***	***0.225***	***0.450***	***DOXO (µM)***	***0.113***	***0.225***	***0.450***
**0**	0.702 ± 0.005	0.712 ± 0.007	0.508 ± 0.004	**0**	0.742 ± 0.016	0.739 ± 0.010	0.501 ± 0.010
**46.55**	0.160 ± 0.006	0.149 ± 0.008	0.143 ± 0.004	**17.68**	0.286 ± 0.009	0.243 ± 0.016	0.237 ± 0.009
**93.10**	0.144 ± 0.012	0.150 ± 0.011	0.147 ± 0.008	**35.35**	0.244 ± 0.011	0.228 ± 0.010	0.239 ± 0.007
**186.20**	0.147 ± 0.008	0.145 ± 0.004	0.139 ± 0.012	**70.70**	0.193 ± 0.006	0.182 ± 0.012	0.188 ± 0.013
**GLIM (µM)**	**U-87MG cell viability**			**GLIQ (µM)**	**U-87MG cell viability**		
DOXO (µM)	***0.260***	***0.520***	***1.040***	***DOXO (µM)***	***0.260***	***1.040***	***1.040***
**0**	0.733 ± 0.003	0.739 ± 0.008	0.501 ± 0.007	**0**	0.752 ± 0.010	0.740 ± 0.002	0.502 ± 0.005
**43.70**	0.277 ± 0.006	0.268 ± 0.011	0.267 ± 0.009	**10.62**	0.317 ± 0.017	0.319 ± 0.025	0.260 ± 0.020
**87.40**	0.272 ± 0.007	0.258 ± 0.025	0.265 ± 0.008	**21.25**	0.302 ± 0.012	0.300 ± 0.016	0.269 ± 0.017
**174.80**	0.255 ± 0.006	0.210 ± 0.007	0.243 ± 0.017	**42.50**	0.245 ± 0.007	0.254 ± 0.006	0.246 ± 0.10

Figure S13 cont. Cytotoxicity results for GLIM, GLIQ, and CHLO on various cell lines

**CHLO (μM)**	**A549 cell viability**		
DOXO (μM)	**0.073**	***0.145***	***0.290***
**0**	0.831 ± 0.004	0.643 ± 0.002	0.502 ± 0.010
**125**	0.673 ± 0.005	0.541 ± 0.004	0.444 ± 0.001
**250**	0.580 ± 0.002	0.464 ± 0.003	0.233 ± 0.005
**500**	0.405 ± 0.008	0.275 ± 0.004	0.170 ± 0.005
**CHLO (μM)**	**HepG2 cell viability**		
DOXO (μM)	***0.180***	***0.360***	***0.720***
**0**	0.791 ± 0.008	0.695 ± 0.003	0.506 ± 0.013
**112.78**	0.405 ± 0.029	0.263 ± 0.004	0.099 ± 0.012
**255.55**	0.397 ± 0.027	0.264 ± 0.002	0.094 ± 0.006
**451.10**	0.349 ± 0.032	0.200 ± 0.014	0.096 ± 0.003
**CHLO (μM)**	**HMEC-1 cell viability**		
DOXO (μM)	***0.020***	***0.040***	***0.080***
**0**	0.813 ± 0.004	0.605 ± 0.002	0.510 ± 0.002
**51.33**	0.759 ± 0.005	0.694 ± 0.013	0.607 ± 0.012
**102.65**	0.654 ± 0.001	0.610 ± 0.004	0.544 ± 0.003
**205.30**	0.548 ± 0.008	0.511 ± 0.001	0.448 ± 0.009
**CHLO (μM)**	**HuH7 cell viability**		
DOXO (μM)	***0.163***	***0.325***	***0.650***
**0**	0.943 ± 0.013	0.953 ± 0.005	0.499 ± 0.003
**125**	1.500 ± 0.001	1.801 ± 0.011	2.508 ± 0.014
**250**	1.986 ± 0.019	2.408 ± 0.012	2.804 ± 0.005
**500**	1.985 ± 0.009	2.994 ± 0.017	2.723 ± 0.020
**CHLO (μM)**	**MCF-7 cell viability**		
DOXO (μM)	***0.113***	***0.225***	***0.450***
**0**	0.770 ± 0.012	0.757 ± 0.010	0.508 ± 0.002
**125**	0.257 ± 0.007	0.201 ± 0.004	0.157 ± 0.004
**250**	0.247 ± 0.012	0.201 ± 0.007	0.152 ± 0.005
**500**	0.239 ± 0.005	0.152 ± 0.006	0.135 ± 0.004
**CHLO (μM)**	**U-87MG cell viability**		
DOXO (μM)	***0.260***	***1.040***	***1.040***
**0**	0.706 ± 0.006	0.764 ± 0.007	0.496 ± 0.007
**125**	0.498 ± 0.005	0.397 ± 0.009	0.303 ± 0.005
**250**	0.494 ± 0.008	0.400 ± 0.003	0.311 ± 0.007
**500**	0.494 ± 0.003	0.402 ± 0.003	0.295 ± 0.011

Figure S14. Synergistic effects of GLIM, GLIQ, and CHLO on various cell lines

GLIM (µM)	A549 Combination Index			GLIQ (µM)	A549 Combination Index		
DOXO (µM)	***0.073***	***0.145***	***0.290***	***DOXO (µM)***	***0.073***	***0.145***	***0.290***
**95.13**	1.214 ± 0.601	1.078 ± 0.176	0.073 ± 0.021	**81.30**	0.260 ± 0.013	0.187 ± 0.017	0.129 ± 0.004
**190.25**	0.611 ± 0.088	0.590 ± 0.083	0.099 ± 0.032	**162.60**	0.383 ± 0.031	0.342 ± 0.013	0.223 ± 0.023
**380.50**	0.749 ± 0.142	0.513 ± 0.098	0.052 ± 0.017	**325.20**	0.679 ± 0.046	0.535 ± 0.034	0.406 ± 0.045
**GLIM (µM)**	**HepG2 Combination Index**			**GLIQ (µM)**	**HepG2 Combination Index**		
DOXO (µM)	***0.180***	***0.360***	***0.720***	***DOXO (µM)***	***0.180***	***0.360***	***0.720***
**48.20**	0.924 ± 0.179	1.352 ± 0.307	0.621 ± 0.087	**8.48**	0.172 ± 0.015	0.089 ± 0.023	0.133 ± 0.038
**96.40**	1.218 ± 0.219	1.097 ± 0.167	0.336 ± 0.043	**16.95**	0.217 ± 0.020	0.123 ± 0.035	0.163 ± 0.033
**192.80**	0.441 ± 0.147	0.584 ± 0.189	0.317 ± 0.046	**33.90**	0.211 ± 0.036	0.184 ± 0.061	0.233 ± 0.054
**GLIM (µM)**	**HMEC-1 Combination Index**			**GLIQ (µM)**	**HMEC-1 Combination Index**		
DOXO (µM)	***0.020***	***0.040***	***0.080***	***DOXO (µM)***	***0.020***	***0.040***	***0.080***
**34.70**	3.473 ± 3.142	3.753 ± 2.423	0.772 ± 0.489	**38.78**	65.02 ± 80.44	46.54 ± 51.83	41.75 ± 43.64
**69.40**	1.561 ± 0.478	0.265 ± 0.077	0.347 ± 0.189	**77.55**	10.61 ± 9.832	8.753 ± 7.835	7.154 ± 6.044
**138.80**	0.193 ± 0.074	0.099 ± 0.013	0.072 ± 0.028	**155.10**	4.883 ± 3.531	2.146 ± 1.046	2.138 ± 1.236
**GLIM (µM)**	**HuH7 Combination Index**			**GLIQ (µM)**	**HuH7 Combination Index**		
DOXO (µM)	***0.163***	***0.325***	***0.650***	***DOXO (µM)***	***0.163***	***0.325***	***0.650***
**115.83**	97.73 ± 54.21	69.14 ± 27.34	32.60 ± 8.051	**106.10**	>100	>100	>100
**231.65**	4.097 ± 0.602	1.358 ± 0.098	1.349 ± 0.131	**212.20**	4.014 ± 1.849	39.52 ± 24.24	>100
**463.30**	1.019 ± 0.047	0.609 ± 0.052	0.459 ± 0.056	**424.40**	0.645 ± 0.005	0.765 ± 0.028	35.61 ± 16.27
**GLIM (µM)**	**MCF-7 Combination Index**			**GLIQ (µM)**	**MCF-7 Combination Index**		
DOXO (µM)	***0.113***	***0.225***	***0.450***	***DOXO (µM)***	***0.113***	***0.225***	***0.450***
**46.55**	0.030 ± 0.003	0.033 ± 0.008	0.044 ± 0.012	**17.68**	0.169 ± 0.004	0.162 ± 0.014	0.193 ± 0.017
**93.10**	0.043 ± 0.009	0.054 ± 0.013	0.065 ± 0.016	**35.35**	0.268 ± 0.005	0.270 ± 0.009	0.317 ± 0.005
**186.20**	0.081 ± 0.011	0.086 ± 0.009	0.093 ± 0.024	**70.70**	0.429 ± 0.006	0.420 ± 0.009	0.453 ± 0.035
**GLIM (µM)**	**U-87MG Combination Index**			**GLIQ (µM)**	**U-87MG Combination Index**		
DOXO (µM)	***0.260***	***0.520***	***1.040***	***DOXO (µM)***	***0.260***	***1.040***	***1.040***
**43.70**	0.117 ± 0.004	0.115 ± 0.011	0.117 ± 0.011	**10.62**	0.131 ± 0.011	0.136 ± 0.015	0.102 ± 0.008
**87.40**	0.228 ± 0.012	0.220 ± 0.032	0.226 ± 0.017	**21.25**	0.239 ± 0.007	0.240 ± 0.014	0.209 ± 0.013
**174.80**	0.425 ± 0.017	0.352 ± 0.022	0.410 ± 0.046	**42.50**	0.369 ± 0.021	0.387 ± 0.022	0.374 ± 0.030

Figure S14 cont. Synergistic effects of GLIM, GLIQ, and CHLO on various cell lines

**CHLO (μM)**	**A549 Combination Index**		
DOXO (μM)	***0.073***	***0.145***	***0.290***
**125**	2.504 ± 0.382	0.866 ± 0.185	0.513 ± 0.121
**250**	0.729 ± 0.092	0.337 ± 0.087	0.026 ± 0.009
**500**	0.086 ± 0.037	0.027 ± 0.012	0.008 ± 0.004
**CHLO (μM)**	**HepG2 Combination Index**		
DOXO (μM)	***0.180***	***0.360***	***0.720***
**112.78**	0.287 ± 0.035	0.207 ± 0.022	0.110 ± 0.018
**255.55**	0.394 ± 0.037	0.250 ± 0.033	0.110 ± 0.015
**451.10**	0.458 ± 0.105	0.207 ± 0.059	0.123 ± 0.012
**CHLO (μM)**	**HMEC-1 Combination Index**		
DOXO (μM)	***0.020***	***0.040***	***0.080***
**51.33**	>100	83.11 ± 75.73	34.89 ± 29.00
**102.65**	24.97 ± 27.09	23.70 ± 25.97	13.99 ± 12.45
**205.30**	4.948 ± 3.744	4.881 ± 3.457	3.483 ± 2.045
**CHLO (μM)**	**HuH7 Combination Index**		
DOXO (μM)	***0.163***	***0.325***	***0.650***
**125**	>100	>100	>100
**250**	>100	>100	>100
**500**	>100	>100	>100
**CHLO (μM)**	**MCF-7 Combination Index**		
DOXO (μM)	***0.113***	***0.225***	***0.450***
**125**	0.024 ± 0.002	0.026 ± 0.009	0.032 ± 0.013
**250**	0.025 ± 0.005	0.027 ± 0.009	0.030 ± 0.013
**500**	0.027 ± 0.007	0.016 ± 0.004	0.025 ± 0.011
**CHLO (μM)**	**U-87MG Combination Index**		
DOXO (μM)	***0.260***	***1.040***	***1.040***
**125**	0.498 ± 0.067	0.248 ± 0.086	0.168 ± 0.035
**250**	0.690 ± 0.063	0.433 ± 0.093	0.330 ± 0.060
**500**	1.118 ± 0.166	0.807 ± 0.134	0.618 ±0.099

Figure S15. Median-effect plots of GLIM, GLIQ and CHLO on various cell lines

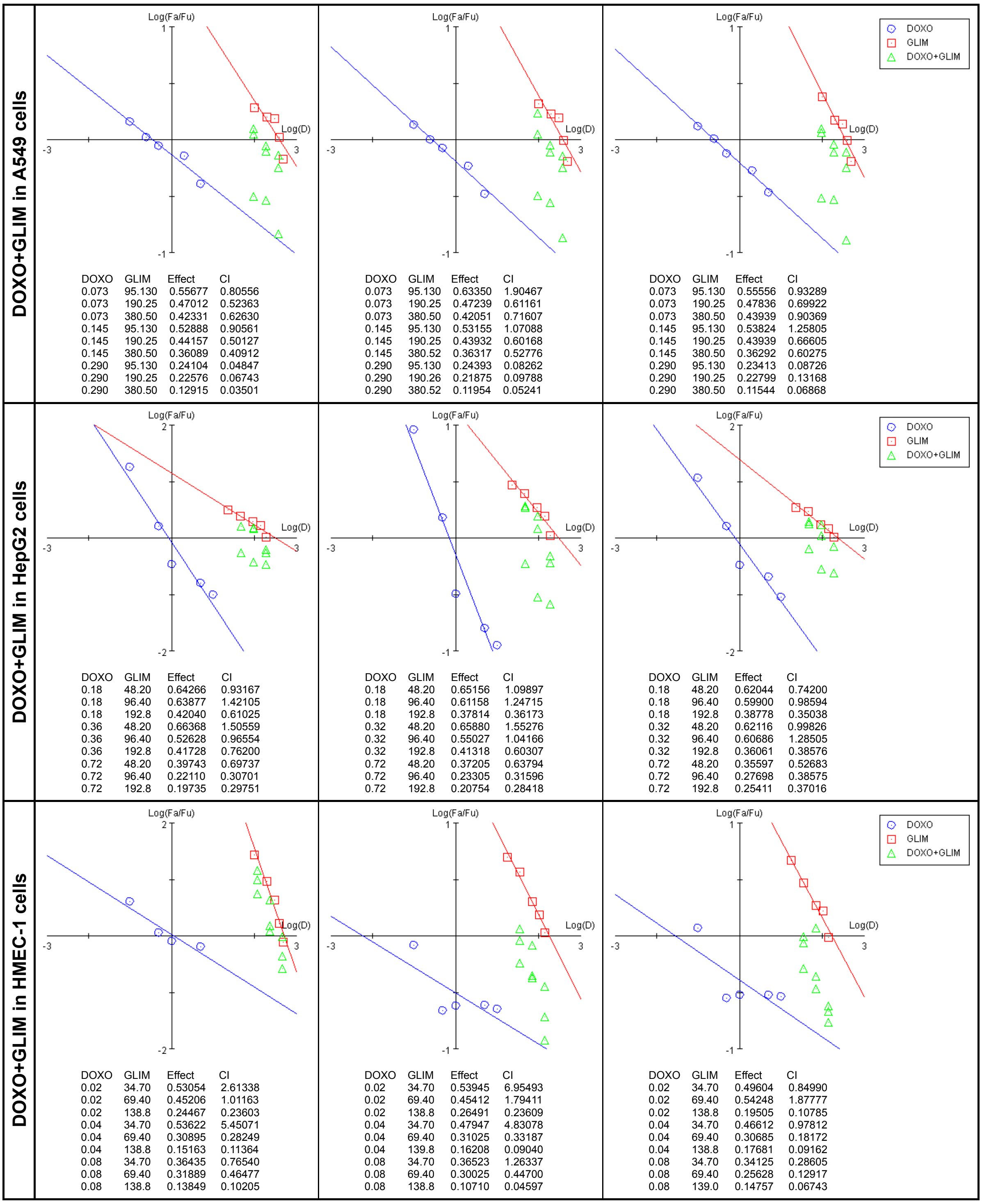

DOXO+GLIM in A549 cells

DOXO	GLIM	Effect	CI
0.073	95.130	0.55677	0.80556
0.073	190.25	0.47012	0.52363
0.073	380.50	0.42331	0.62630
0.145	95.130	0.52888	0.90561
0.145	190.25	0.44157	0.50127
0.145	380.50	0.36089	0.40912
0.290	95.130	0.24104	0.04847
0.290	190.25	0.22576	0.06743
0.290	380.50	0.12915	0.03501

DOXO	GLIM	Effect	CI
0.073	95.130	0.63350	1.90467
0.073	190.25	0.47239	0.61161
0.073	380.50	0.42051	0.71607
0.145	95.130	0.53155	1.07088
0.145	190.25	0.43932	0.60168
0.145	380.52	0.36317	0.52776
0.290	95.130	0.24393	0.08262
0.290	190.26	0.21875	0.09788
0.290	380.52	0.11954	0.05241

DOXO	GLIM	Effect	CI
0.073	95.130	0.55556	0.93289
0.073	190.25	0.47836	0.69922
0.073	380.50	0.43939	0.90369
0.145	95.130	0.53824	1.25805
0.145	190.25	0.43939	0.66605
0.145	380.50	0.36292	0.60275
0.290	95.130	0.23413	0.08726
0.290	190.25	0.22799	0.13168
0.290	380.50	0.11544	0.06868

DOXO+GLIM in HepG2 cells

DOXO	GLIM	Effect	CI
0.18	48.20	0.64266	0.93167
0.18	96.40	0.63877	1.42105
0.18	192.8	0.42040	0.61025
0.36	48.20	0.66368	1.50559
0.36	96.40	0.52628	0.96554
0.36	192.8	0.41728	0.76200
0.72	48.20	0.39743	0.69737
0.72	96.40	0.22110	0.30701
0.72	192.8	0.19735	0.29751

DOXO	GLIM	Effect	CI
0.18	48.20	0.65156	1.09897
0.18	96.40	0.61158	1.24715
0.18	192.8	0.37814	0.36173
0.32	48.20	0.65880	1.55276
0.32	96.40	0.55027	1.04166
0.32	192.8	0.41318	0.60307
0.72	48.20	0.37205	0.63794
0.72	96.40	0.23305	0.31596
0.72	192.8	0.20754	0.28418

DOXO	GLIM	Effect	CI
0.18	48.20	0.62044	0.74200
0.18	96.40	0.59900	0.98594
0.18	192.8	0.38778	0.35038
0.32	48.20	0.62116	0.99826
0.32	96.40	0.60686	1.28505
0.32	192.8	0.36061	0.38576
0.72	48.20	0.35597	0.52683
0.72	96.40	0.27698	0.38575
0.72	192.8	0.25411	0.37016

DOXO+GLIM in HMEC-1 cells

DOXO	GLIM	Effect	CI
0.02	34.70	0.53054	2.61338
0.02	69.40	0.45206	1.01163
0.02	138.8	0.24467	0.23603
0.04	34.70	0.53622	5.45071
0.04	69.40	0.30895	0.28249
0.04	138.8	0.15163	0.11364
0.08	34.70	0.36435	0.76540
0.08	69.40	0.31889	0.46477
0.08	138.8	0.13849	0.10205

DOXO	GLIM	Effect	CI
0.02	34.70	0.53945	6.95493
0.02	69.40	0.45412	1.79411
0.02	138.8	0.26491	0.23609
0.04	34.70	0.47947	4.83078
0.04	69.40	0.31025	0.33187
0.04	139.8	0.16208	0.09040
0.08	34.70	0.36523	1.26337
0.08	69.40	0.30025	0.44700
0.08	138.8	0.10710	0.04597

DOXO	GLIM	Effect	CI
0.02	34.70	0.49604	0.84990
0.02	69.40	0.54248	1.87777
0.02	138.8	0.19505	0.10785
0.04	34.70	0.46612	0.97812
0.04	69.40	0.30685	0.18172
0.04	138.8	0.17681	0.09162
0.08	34.70	0.34125	0.28605
0.08	69.40	0.25628	0.12917
0.08	139.0	0.14757	0.06743

Figure S15 cont. Median-effect plots of GLIM, GLIQ and CHLO on various cell lines

DOXO+GLIM in HuH7 cells

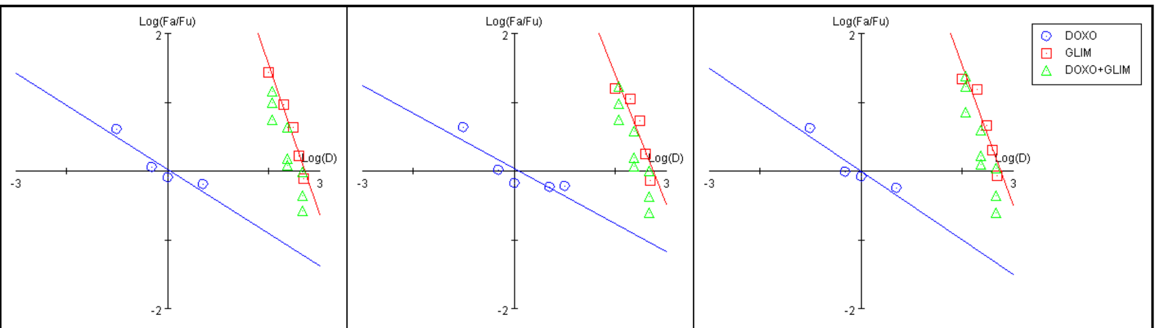

DOXO	GLIM	Effect	CI
0.163	115.83	0.93635	45.0695
0.163	231.65	0.81591	4.31916
0.163	463.30	0.49953	1.04149
0.325	115.83	0.91014	40.4680
0.325	231.65	0.60624	1.26391
0.325	463.30	0.30577	0.66486
0.650	115.83	0.84992	23.5227
0.650	231.65	0.54945	1.36188
0.650	463.30	0.20998	0.52054

DOXO	Glim	Effect	CI
0.163	115.83	0.94552	153.363
0.163	231.65	0.79493	4.55572
0.163	463.30	0.50362	0.96430
0.325	115.83	0.90631	72.0420
0.325	231.65	0.61520	1.35145
0.325	463.30	0.30338	0.56162
0.650	115.83	0.85062	38.8714
0.650	231.65	0.53981	1.21162
0.650	463.30	0.20145	0.41207

DOXO	GLIM	Effect	CI
0.163	115.83	0.96027	94.7597
0.163	231.65	0.80112	3.41587
0.163	463.30	0.52673	1.05006
0.325	115.83	0.94487	94.9115
0.325	231.65	0.63033	1.45917
0.325	463.30	0.30622	0.60018
0.650	115.83	0.88059	35.4140
0.650	231.65	0.55752	1.47230
0.650	463.30	0.19846	0.44298

DOXO+GLIM in MCF-7 cells

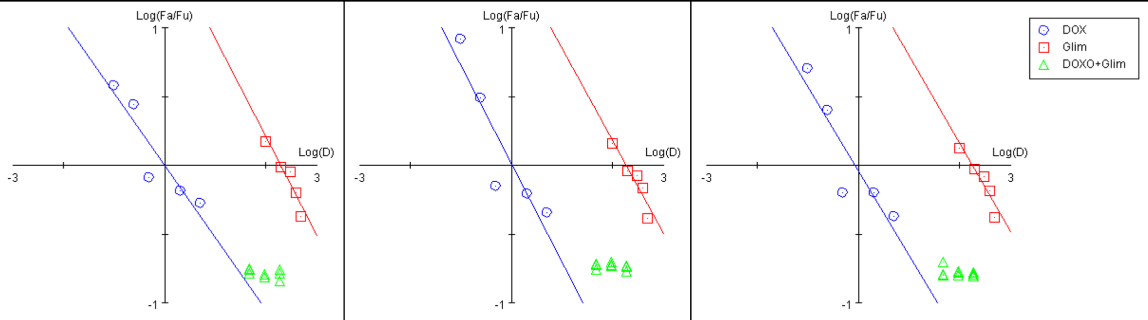

DOXO	GLIM	Effect	CI
0.1125	46.55	0.15224	0.02581
0.1125	93.10	0.13340	0.03789
0.1125	186.2	0.14104	0.07955
0.225	46.55	0.14868	0.02884
0.225	93.10	0.14053	0.04493
0.225	186.2	0.14969	0.09199
0.450	46.55	0.14104	0.03347
0.450	93.10	0.14002	0.05177
0.450	186.2	0.12627	0.07486

DOXO	GLIM	Effect	CI
0.1125	46.55	0.16146	0.03239
0.1125	93.10	0.16030	0.05317
0.1125	186.2	0.15799	0.09312
0.225	46.55	0.15914	0.04226
0.225	93.10	0.16609	0.06805
0.225	186.2	0.14525	0.08962
0.450	46.55	0.14873	0.05677
0.450	93.10	0.15741	0.08296
0.450	186.2	0.15509	0.12084

DOXO	GLIM	Effect	CI
0.1125	46.55	0.16650	0.03089
0.1125	93.10	0.13841	0.03708
0.1125	186.2	0.14300	0.07156
0.225	46.55	0.13994	0.02922
0.225	93.10	0.14454	0.04744
0.225	186.2	0.13994	0.07566
0.450	46.55	0.13841	0.04208
0.450	93.10	0.14351	0.06123
0.450	186.2	0.13585	0.08467

DOXO+GLIM in U-87MG cells

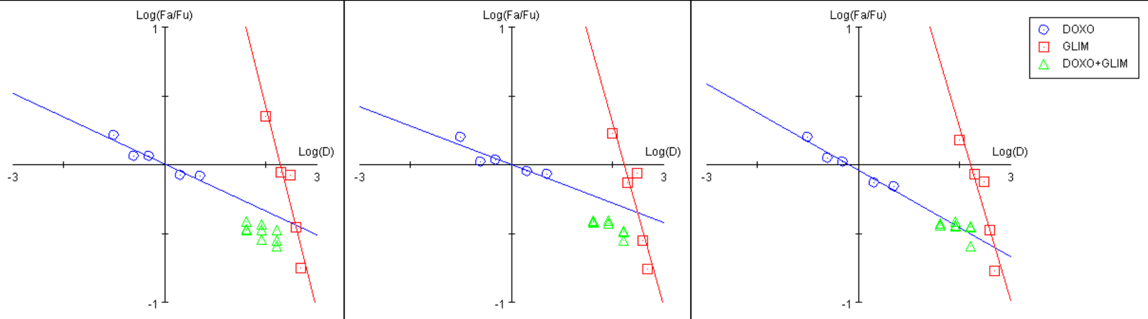

DOXO	GLIM	Effect	CI
0.26	43.70	0.27967	0.11299
0.26	87.40	0.27149	0.21858
0.26	174.8	0.25321	0.40869
0.52	43.70	0.25184	0.10240
0.52	87.40	0.22347	0.18271
0.52	174.8	0.20464	0.33791
1.04	43.70	0.25539	0.10476
1.04	87.40	0.25348	0.20611
1.04	174.8	0.21965	0.35983

DOXO	GLIM	Effect	CI
0.26	43.70	0.28325	0.12168
0.26	87.40	0.28158	0.24151
0.26	174.8	0.24847	0.42399
0.52	43.70	0.27713	0.11910
0.52	87.40	0.27240	0.23338
0.52	174.8	0.22065	0.37682
1.04	43.70	0.27852	0.12029
1.04	87.40	0.27295	0.23432
1.04	174.8	0.24708	0.42184

DOXO	GLIM	Effect	CI
0.26	43.70	0.26813	0.11554
0.26	87.40	0.26331	0.22305
0.26	174.8	0.26278	0.44218
0.52	43.70	0.27402	0.12252
0.52	87.40	0.27883	0.24286
0.52	174.8	0.20551	0.34144
1.04	43.70	0.26786	0.12518
1.04	87.40	0.26706	0.23633
1.04	174.8	0.26171	0.44859

Figure S15 cont. Median-effect plots of GLIM, GLIQ and CHLO on various cell lines

DOXO+GLIQ in A549 cells

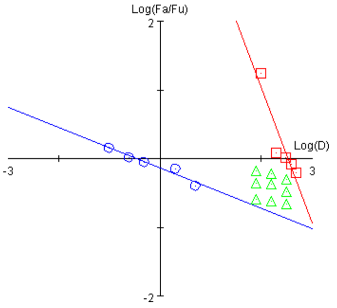

DOXO	GLIQ	Effect	CI
0.073	81.30	0.40183	0.25050
0.073	162.6	0.37700	0.41134
0.073	325.2	0.33383	0.69905
0.145	81.30	0.30751	0.18557
0.145	162.6	0.30337	0.34044
0.145	325.2	0.25222	0.56516
0.290	81.30	0.20727	0.13031
0.290	162.6	0.19781	0.24342
0.290	325.2	0.18037	0.45094

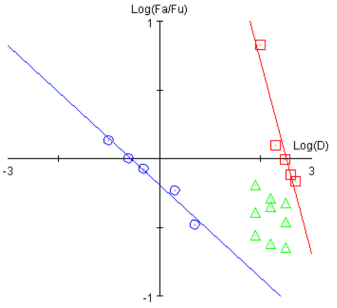

DOXO	GLIQ	Effect	CI
0.073	81.30	0.39077	0.25448
0.073	162.6	0.33930	0.34958
0.073	325.2	0.32448	0.62651
0.145	81.30	0.29035	0.17051
0.145	162.6	0.30825	0.33098
0.145	325.2	0.25902	0.49869
0.290	81.30	0.21678	0.12415
0.290	162.6	0.19469	0.19832
0.290	325.2	0.18434	0.36079

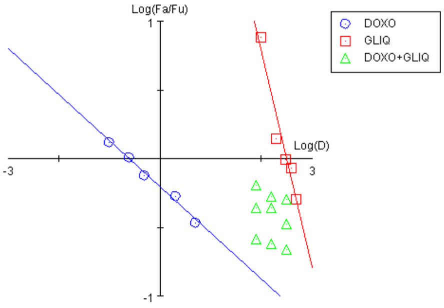

DOXO	GLIQ	Effect	CI
0.073	81.30	0.39217	0.27459
0.073	162.6	0.34638	0.38808
0.073	325.2	0.33839	0.71190
0.145	81.30	0.30564	0.20397
0.145	162.6	0.30511	0.35582
0.145	325.2	0.25346	0.54186
0.290	81.30	0.20687	0.13119
0.290	162.6	0.19489	0.22639
0.290	325.2	0.18024	0.40649

DOXO+GLIQ in HepG2 cells

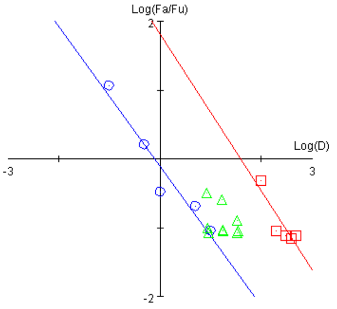

DOXO	GLIQ	Effect	CI
0.18	8.475	0.24364	0.15470
0.18	16.95	0.20686	0.19405
0.18	33.90	0.11280	0.17249
0.36	8.475	0.09194	0.07671
0.36	16.94	0.08699	0.10019
0.36	33.90	0.07885	0.14130
0.72	8.475	0.07921	0.10617
0.72	16.95	0.08239	0.13698
0.72	33.90	0.08239	0.18967

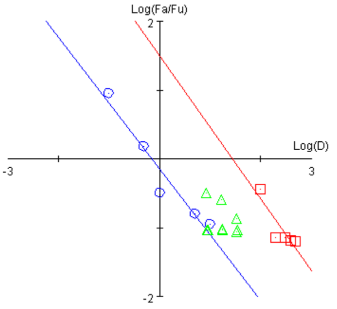

DOXO	GLIQ	Effect	CI
0.18	8.475	0.24546	0.18364
0.18	16.95	0.20642	0.23164
0.18	33.90	0.12138	0.21639
0.36	8.475	0.08581	0.07467
0.36	16.95	0.08581	0.10644
0.36	33.90	0.08002	0.15768
0.72	8.475	0.08504	0.11638
0.72	16.95	0.08736	0.15232
0.72	33.90	0.08659	0.21494

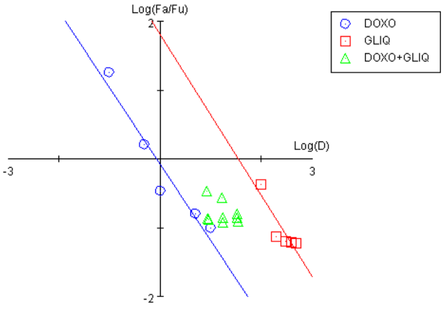

DOXO	GLIQ	Effect	CI
0.18	8.475	0.25766	0.17825
0.18	16.95	0.21488	0.22501
0.18	33.90	0.13807	0.24306
0.36	8.475	0.12154	0.11473
0.36	16.95	0.12445	0.16340
0.36	33.90	0.12397	0.25415
0.72	8.475	0.11619	0.17641
0.72	16.95	0.10647	0.20113
0.72	33.90	0.11181	0.29385

DOXO+GLIQ in HMEC-1 cells

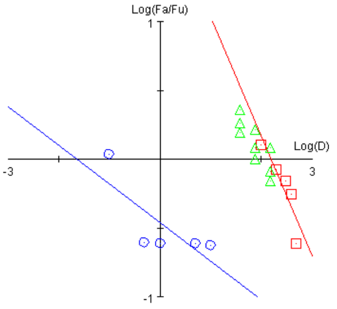

DOXO	GLIQ	Effect	CI
0.02	38.78	0.69630	17.0541
0.02	77.55	0.62272	5.99795
0.02	155.1	0.54963	3.01028
0.04	38.78	0.64790	15.5229
0.04	77.55	0.55012	4.14892
0.04	155.0	0.44938	1.63068
0.08	38.78	0.60988	17.2464
0.08	77.55	0.50321	4.11029
0.08	155.0	0.40988	1.60207

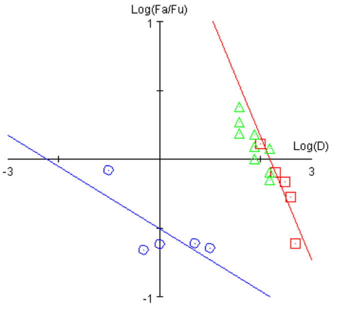

DOXO	GLIQ	Effect	CI
0.02	38.78	0.70553	157.878
0.02	77.55	0.60302	21.9032
0.02	155.1	0.54724	8.95552
0.04	38.78	0.65176	106.375
0.04	77.55	0.55327	17.7988
0.04	155.1	0.44573	3.35007
0.08	38.78	0.60754	92.1345
0.08	77.55	0.50101	14.1150
0.08	155.1	0.41357	3.55192

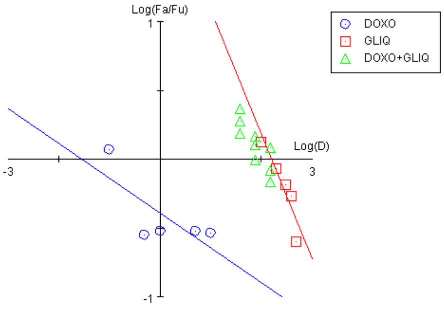

DOXO	GLIQ	Effect	CI
0.02	38.78	0.70000	20.1178
0.02	77.55	0.59563	3.93584
0.02	155.1	0.54762	2.68305
0.04	38.78	0.65476	17.7212
0.04	77.55	0.56151	4.30983
0.04	155.1	0.45317	1.45788
0.08	38.78	0.60754	15.8711
0.08	77.55	0.49960	3.23810
0.08	155.1	0.40595	1.26141

Figure S15 cont. Median-effect plots of GLIM, GLIQ and CHLO on various cell lines

DOXO+GLIQ in HUH7 cells

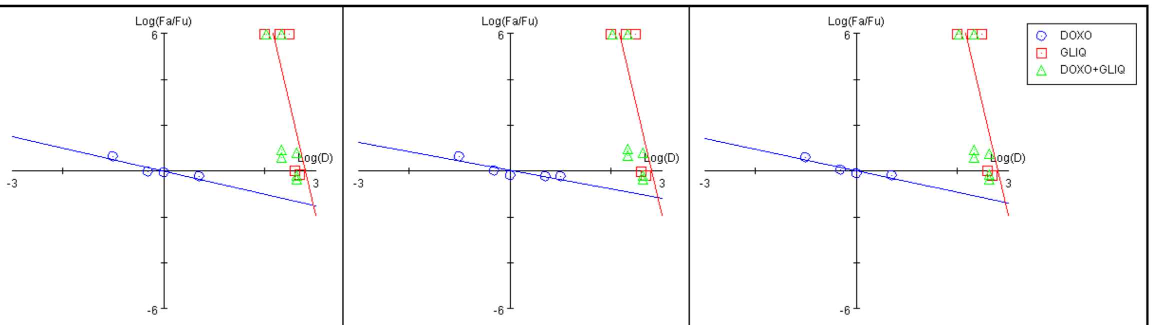

DOXO	GLIQ	Effect	CI
0.163	106.10	1.00000	1.59E11
0.163	212.20	0.79571	2.83378
0.163	424.40	0.29831	0.64911
0.325	106.10	1.00000	3.16E11
0.325	212.20	0.89526	23.8472
0.325	424.40	0.39336	0.78365
0.650	106.10	1.00000	6.32E11
0.650	212.20	1.00000	6.32E11
0.650	424.40	0.86554	27.4003

DOXO	GLIQ	Effect	CI
0.163	106.10	1.00000	9.71E13
0.163	212.20	0.82224	6.14448
0.163	424.40	0.30060	0.63892
0.325	106.10	1.00000	1.94E14
0.325	212.20	0.90414	67.4419
0.325	424.40	0.38855	0.73290
0.650	106.10	1.00000	3.87E14
0.650	212.20	1.00000	3.87E14
0.650	424.40	0.86691	54.3560

DOXO	GLIQ	Effect	CI
0.163	106.10	1.00000	9.14E11
0.163	212.20	0.79781	3.06230
0.163	424.40	0.30716	0.64731
0.325	106.10	1.00000	1.82E12
0.325	212.20	0.89387	27.2813
0.325	424.40	0.40667	0.77867
0.650	106.10	1.00000	3.64E12
0.650	212.20	1.00000	3.64E12
0.650	424.40	0.85303	25.0795

DOXO+GLIQ in MCF-7 cells

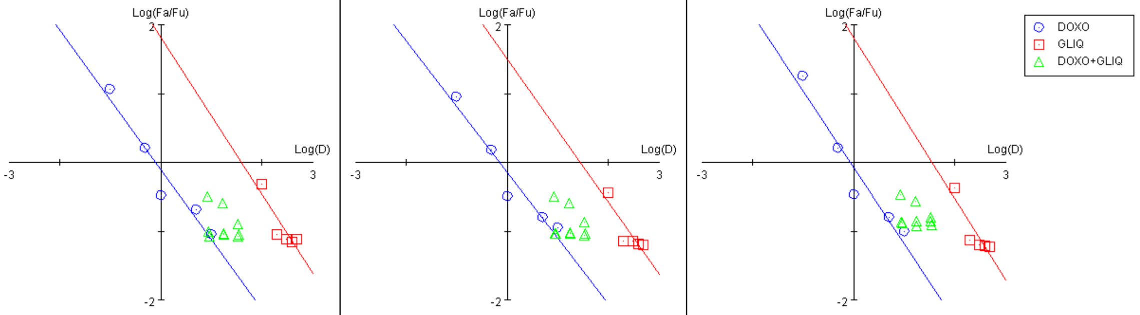

DOXO	GLIQ	Effect	CI
0.113	17.68	0.28565	0.17028
0.113	35.35	0.23703	0.26963
0.113	70.70	0.18747	0.43494
0.225	17.68	0.24030	0.15538
0.225	35.35	0.22581	0.26914
0.225	70.70	0.18093	0.42932
0.450	17.68	0.23796	0.17829
0.450	35.35	0.24544	0.31800
0.450	70.70	0.20383	0.49029

DOXO	GLIQ	Effect	CI
0.113	17.68	0.29718	0.17220
0.113	35.35	0.25837	0.27308
0.113	70.70	0.20202	0.42501
0.225	17.68	0.26369	0.17802
0.225	35.35	0.24136	0.27883
0.225	70.70	0.19777	0.43331
0.450	17.68	0.24774	0.21220
0.450	35.35	0.24083	0.32202
0.450	70.70	0.18926	0.44658

DOXO	GLIQ	Effect	CI
0.113	17.68	0.27413	0.16502
0.113	35.35	0.23515	0.26260
0.113	70.70	0.19059	0.42566
0.225	17.68	0.22525	0.15285
0.225	35.35	0.21782	0.26185
0.225	70.70	0.16832	0.39607
0.450	17.68	0.22587	0.18879
0.450	35.35	0.22958	0.31211
0.450	70.70	0.17141	0.42217

DOXO+GLIQ in U-87MG cells

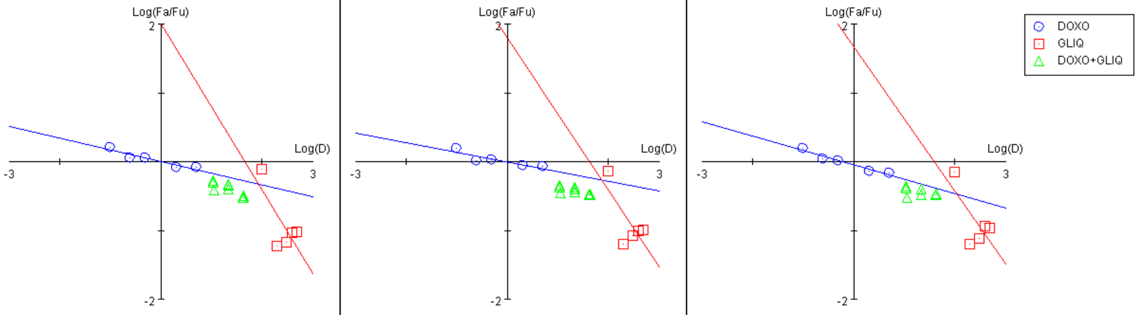

DOXO	GLIQ	Effect	CI
0.26	10.62	0.33900	0.13694
0.26	21.25	0.31747	0.24643
0.26	42.50	0.23506	0.34642
0.52	10.62	0.35316	0.15311
0.52	21.25	0.32172	0.25382
0.52	42.50	0.24526	0.36319
1.04	10.62	0.28377	0.11109
1.04	21.25	0.29029	0.22438
1.04	42.50	0.23195	0.34214

DOXO	GLIQ	Effect	CI
0.26	10.62	0.30051	0.11812
0.26	21.25	0.29840	0.23351
0.26	42.50	0.24977	0.37386
0.52	10.62	0.31139	0.12475
0.52	21.25	0.28994	0.22538
0.52	42.50	0.25914	0.39101
1.04	10.62	0.26155	0.09937
1.04	21.25	0.26669	0.20304
1.04	42.50	0.25128	0.37687

DOX	GLIQ	Effect	CI
0.26	10.62	0.31099	0.13720
0.26	21.25	0.28891	0.23759
0.26	42.50	0.25013	0.38711
0.52	10.62	0.29321	0.13031
0.52	21.25	0.28729	0.24083
0.52	42.50	0.25875	0.40722
1.04	10.62	0.23560	0.09504
1.04	21.25	0.24987	0.20058
1.04	42.50	0.25417	0.40224

Figure S15 cont. Median-effect plots of GLIM, GLIQ and CHLO on various cell lines

DOXO+CHLO in A549 cells

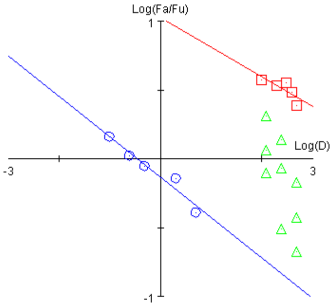

DOXO	CHLO	Effect	CI
0.073	125.0	0.67427	2.54130
0.073	250.0	0.58016	0.64213
0.073	500.0	0.40237	0.05480
0.145	125.0	0.53677	0.68827
0.145	250.0	0.46295	0.25113
0.145	500.0	0.27332	0.01475
0.290	125.0	0.44379	0.38340
0.290	250.0	0.23725	0.01535
0.290	500.0	0.17554	0.00421

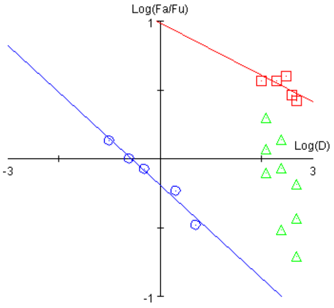

DOXO	CHLO	Effect	CI
0.073	125.0	0.66694	2.10424
0.073	250.0	0.58237	0.71931
0.073	500.0	0.39621	0.07651
0.145	125.0	0.53981	0.85241
0.145	250.0	0.46101	0.33451
0.145	500.0	0.27128	0.02844
0.290	125.0	0.44206	0.53243
0.290	250.0	0.23558	0.03249
0.290	500.0	0.16392	0.00851

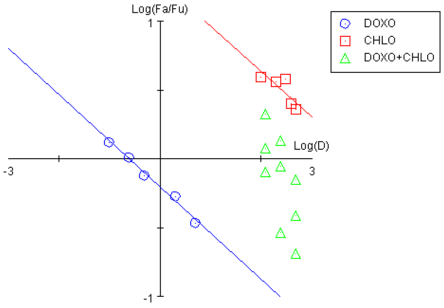

DOXO	CHLO	Effect	CI
0.073	125.0	0.67853	2.86644
0.073	250.0	0.57786	0.82498
0.073	500.0	0.41515	0.12606
0.145	125.0	0.54684	1.05735
0.145	250.0	0.46867	0.42523
0.145	500.0	0.27981	0.03846
0.290	125.0	0.44556	0.62256
0.290	250.0	0.22628	0.03099
0.290	500.0	0.17123	0.01123

DOXO+CHLO in HepG2 cells

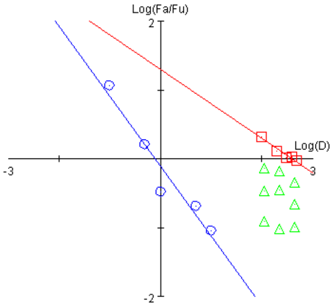

DOXO	CHLO	Effect	CI
0.18	112.78	0.42383	0.31903
0.18	225.55	0.39832	0.39615
0.18	451.10	0.31376	0.33848
0.36	112.78	0.25805	0.19408
0.36	225.55	0.26174	0.23356
0.36	451.10	0.18054	0.15633
0.72	112.78	0.11174	0.12196
0.72	225.55	0.08758	0.09515
0.72	451.10	0.09362	0.10866

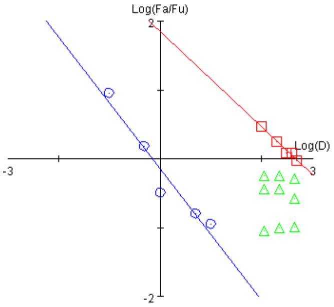

DOXO	CHLO	Effect	CI
0.18	112.78	0.36401	0.24878
0.18	225.55	0.36401	0.35573
0.18	451.10	0.34214	0.50094
0.36	112.78	0.26707	0.23276
0.36	225.55	0.26669	0.28788
0.36	451.10	0.21162	0.27225
0.72	112.78	0.08223	0.08911
0.72	225.55	0.09166	0.11007
0.72	451.10	0.09430	0.13231

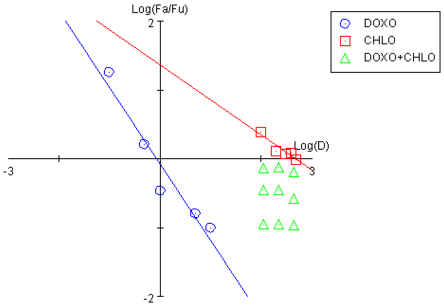

DOXO	CHLO	Effect	CI
0.18	112.78	0.42726	0.29234
0.18	225.55	0.42909	0.43002
0.18	451.10	0.39113	0.53511
0.36	112.78	0.26258	0.19493
0.36	225.55	0.26349	0.22723
0.36	451.10	0.20860	0.19369
0.72	112.78	0.10156	0.11973
0.72	225.55	0.10247	0.12417
0.72	451.10	0.09973	0.12689

DOXO+CHLO in HMEC-1 cells

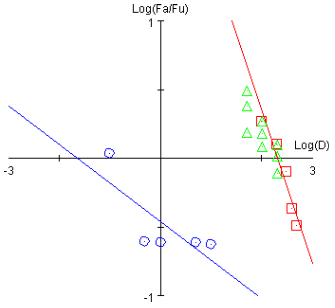

DOXO	CHLO	Effect	CI
0.02	51.330	0.75771	50.1118
0.02	102.65	0.65305	9.01923
0.02	205.30	0.55699	3.16609
0.04	51.330	0.70753	40.2443
0.04	102.65	0.60502	8.55821
0.04	205.30	0.51075	3.04031
0.08	51.330	0.60824	16.7914
0.08	102.65	0.54875	7.48694
0.08	205.30	0.43978	2.25644

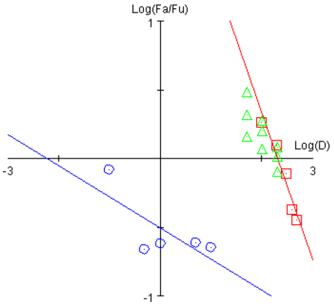

DOXO	CHLO	Effect	CI
0.02	51.330	0.75335	459.057
0.02	102.65	0.65404	56.2454
0.02	205.30	0.54965	9.25001
0.04	51.330	0.67575	170.548
0.04	102.65	0.61524	53.6852
0.04	205.30	0.50901	8.86942
0.08	51.330	0.59122	68.3396
0.08	102.65	0.54134	28.3370
0.08	205.30	0.44480	5.84420

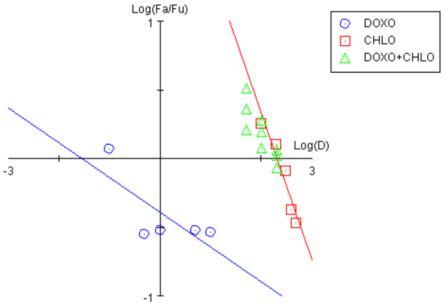

DOXO	CHLO	Effect	CI
0.02	51.330	0.76481	72.8669
0.02	102.65	0.65556	9.64964
0.02	205.30	0.53843	2.42702
0.04	51.330	0.69861	38.5291
0.04	102.65	0.61019	8.84661
0.04	205.30	0.51250	2.73414
0.08	51.330	0.62037	19.5498
0.08	102.65	0.54398	6.13170
0.08	205.30	0.46065	2.34869

Figure S15 cont. Median-effect plots of GLIM, GLIQ and CHLO on various cell lines

DOXO+CHLO in HuH7 cells

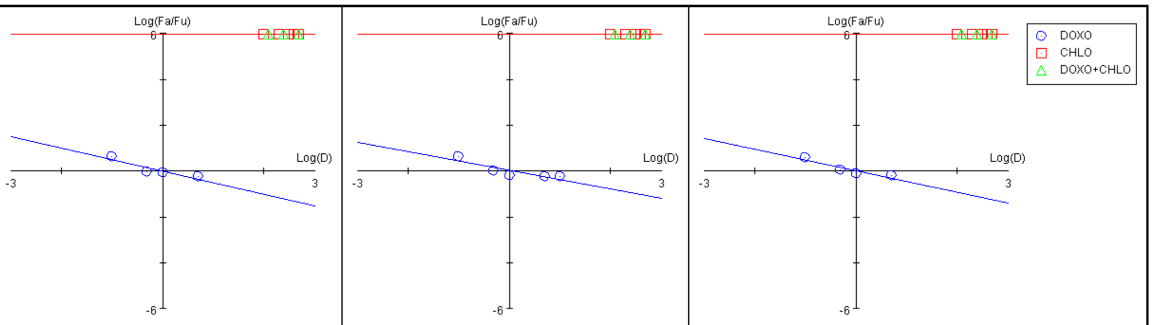

DOXO	CHLO	Effect	CI
0.163	125.0	1.00000	NaN
0.163	250.0	1.00000	NaN
0.163	500.0	1.00000	NaN
0.325	125.0	1.00000	NaN
0.325	250.0	1.00000	NaN
0.325	500.0	1.00000	NaN
0.650	125.0	1.00000	NaN
0.650	250.0	1.00000	NaN
0.650	500.0	1.00000	NaN

DOXO	CHLO	Effect	CI
0.163	125.0	1.00000	NaN
0.163	250.0	1.00000	NaN
0.163	500.0	1.00000	NaN
0.325	125.0	1.00000	NaN
0.325	250.0	1.00000	NaN
0.325	500.0	1.00000	NaN
0.650	125.0	1.00000	NaN
0.650	250.0	1.00000	NaN
0.650	500.0	1.00000	NaN

DOXO	CHLO	Effect	CI
0.163	125.0	1.00000	NaN
0.163	250.0	1.00000	NaN
0.163	500.0	1.00000	NaN
0.325	125.0	1.00000	NaN
0.325	250.0	1.00000	NaN
0.325	500.0	1.00000	NaN
0.650	125.0	1.00000	NaN
0.650	250.0	1.00000	NaN
0.650	500.0	1.00000	NaN

DOXO+CHLO in MCF-7 cells

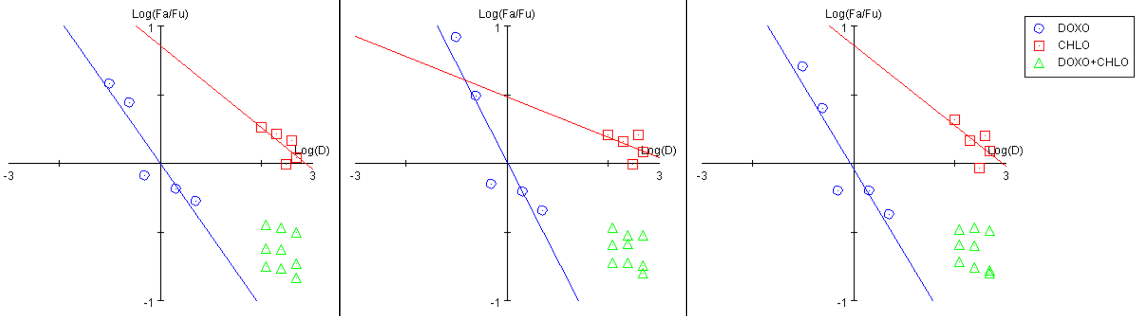

DOXO	CHLO	Effect	CI
0.113	125.0	0.26555	0.02159
0.113	250.0	0.25637	0.02398
0.113	500.0	0.23967	0.02606
0.225	125.0	0.19541	0.01664
0.225	250.0	0.19207	0.01725
0.225	500.0	0.15699	0.01151
0.450	125.0	0.15157	0.01750
0.450	250.0	0.14823	0.01709
0.450	500.0	0.12902	0.01298

DOXO	CHLO	Effect	CI
0.113	125.0	0.25390	0.02418
0.113	250.0	0.23029	0.02021
0.113	500.0	0.23296	0.02068
0.225	125.0	0.20356	0.03243
0.225	250.0	0.20846	0.03382
0.225	500.0	0.15457	0.02035
0.450	125.0	0.15902	0.04265
0.450	250.0	0.15902	0.04265
0.450	500.0	0.13808	0.03387

DOXO	CHLO	Effect	CI
0.113	125.0	0.25014	0.02550
0.113	250.0	0.25477	0.03045
0.113	500.0	0.24552	0.03386
0.225	125.0	0.20451	0.03030
0.225	250.0	0.20104	0.03053
0.225	500.0	0.14327	0.01567
0.450	125.0	0.16176	0.03678
0.450	250.0	0.14905	0.03174
0.450	500.0	0.13865	0.02815

DOXO+CHLO in U-87MG cells

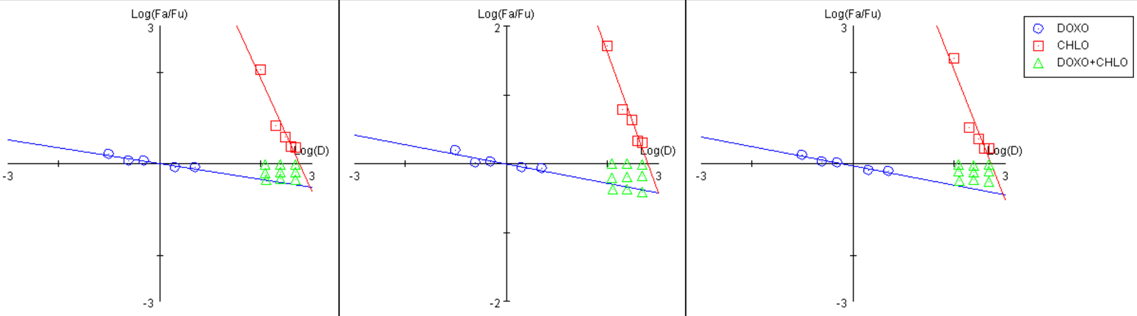

DOXO	CHLO	Effect	CI
0.26	125.0	0.49805	0.44280
0.26	250.0	0.49312	0.63099
0.26	500.0	0.49052	1.04448
0.52	125.0	0.39314	0.21974
0.52	250.0	0.39782	0.40894
0.52	500.0	0.39886	0.77766
1.04	125.0	0.31057	0.16590
1.04	250.0	0.31888	0.32986
1.04	500.0	0.30927	0.63410

DOXO	CHLO	Effect	CI
0.26	125.0	0.50417	0.47892
0.26	250.0	0.50417	0.68239
0.26	500.0	0.49362	1.00059
0.52	125.0	0.38758	0.17937
0.52	250.0	0.39813	0.35433
0.52	500.0	0.40157	0.69080
1.04	125.0	0.29823	0.13434
1.04	250.0	0.30290	0.26985
1.04	500.0	0.28351	0.51183

DOXO	CHLO	Effect	CI
0.26	125.0	0.49210	0.57283
0.26	250.0	0.48487	0.75705
0.26	500.0	0.49665	1.30825
0.52	125.0	0.40964	0.34501
0.52	250.0	0.40482	0.53626
0.52	500.0	0.40669	0.95383
1.04	125.0	0.30174	0.20354
1.04	250.0	0.31084	0.39012
1.04	500.0	0.29103	0.70859

Figure S16. Cell cycle analysis for GLIM, GLIQ, CHLO, and DOXO treatments

**Drug**	**A549 cell cycle distribution**			
Stage	***subG1***	***G1***	***S***	***G2/M***
**CTRL**	2.04 ± 0.18	81.13 ± 7.88	10.27 ± 4.35	6.70 ± 2.24
**GLIM**	8.12 ± 5.97	76.53 ± 12.79	7.55 ± 2.21	6.70 ± 2.75
**GLIQ**	5.19 ± 4.30	80.17 ± 10.98	6.55 ± 3.96	6.13 ± 2.27
**CHLO**	10.71 ± 5.15	70.67 ± 4.83	9.05 ± 2.28	7.59 ± 3.78
**DOXO**	12.11 ± 2.23	71.93 ± 2.61	6.39 ± 2.37	6.41 ± 3.07
**DOXO+GLIM**	16.84 ± 3.22	63.37 ± 6.12	7.39 ± 1.89	9.74 ± 5.02
**DOXO+GLIQ**	13.63 ± 1.02	61.90 ± 7.56	7.74 ± 4.39	16.21 ± 6.33
**DOXO+CHLO**	10.40 ± 2.17	62.03 ± 8.49	9.68 ± 4.72	16.50 ± 8.21
Drug	**HepG2 cell cycle distribution**			
Stage	***subG1***	***G1***	***S***	***G2/M***
**CTRL**	8.77 ± 4.47	70.30 ± 5.87	7.95 ± 3.73	10.25 ± 3.54
**GLIM**	17.17 ± 3.71	63.13 ± 9.32	5.96 ± 3.68	10.51 ± 4.49
**GLIQ**	14.60 ± 5.46	68.87 ± 5.86	6.14 ± 1.45	8.30 ± 1.06
**CHLO**	23.65 ± 15.93	60.63 ± 10.43	6.70 ± 3.13	7.44 ± 2.48
**DOXO**	24.23 ± 13.25	47.67 ± 14.75	10.52 ± 9.09	14.52 ± 8.96
**DOXO+GLIM**	31.20 ± 18.24	45.23 ± 6.20	8.40 ± 6.62	10.91 ± 7.85
**DOXO+GLIQ**	24.43 ± 7.61	46.50 ± 13.99	9.10 ± 6.32	16.66 ± 12.66
**DOXO+CHLO**	27.53 ± 12.98	40.33 ± 14.40	10.09 ± 4.83	18.98 ± 9.64
**Drug**	**MCF-7 cell cycle distribution**			
Stage	***subG1***	***G1***	***S***	***G2/M***
**CTRL**	5.26 ± 3.45	73.93 ± 10.52	11.53 ± 2.17	6.36 ± 1.89
**GLIM**	9.88 ± 5.89	72.50 ± 8.29	10.32 ± 3.74	5.77 ± 2.40
**GLIQ**	8.50 ± 3.31	78.37 ± 2.78	6.88 ± 2.29	5.39 ± 2.42
**CHLO**	9.51 ± 4.64	73.77 ± 7.60	6.20 ± 2.69	10.36 ± 7.09
**DOXO**	8.51 ± 5.50	74.43 ± 13.06	5.71 ± 1.98	11.26 ± 8.76
**DOXO+GLIM**	6.16 ± 3.86	66.03 ± 3.81	9.94 ± 4.20	17.83 ± 4.84
**DOXO+GLIQ**	**10.40** ± 1.73	65.03 ± 3.30	8.03 ± 4.68	16.07 ± 2.39
**DOXO+CHLO**	12.14 ± 4.69	67.43 ± 10.11	7.18 ± 3.65	13.47 ± 9.11
**Drug**	**U-87MG cell cycle distribution**			
Stage	***subG1***	***G1***	***S***	***G2/M***
**CTRL**	2.10 ± 2.08	80.87 ± 5.26	5.65 ± 1.47	9.12 ± 3.00
**GLIM**	1.55 ± 1.51	87.63 ± 3.27	4.00 ± 0.99	6.59 ± 2.18
**GLIQ**	1.96 ± 0.34	78.47 ± 13.63	4.67 ± 1.33	16.67 ± 15.93
**CHLO**	3.84 ± 1.62	73.83 ± 12.39	7.82 ± 5.48	10.65 ± 8.19
**DOXO**	5.20 ± 3.02	80.13 ± 6.63	4.17 ± 1.16	10.18 ± 8.03
**DOXO+GLIM**	11.53 ± 9.67	40.23 ± 14.04	7.25 ± 3.00	37.37 ± 5.81
**DOXO+GLIM**	11.27 ± 6.66	38.33 ± 1.42	6.51 ± 4.16	43.13 ± 2.30
**DOXO+GLIM**	20.90 ± 7.61	38.70 ± 12.11	6.63 ± 4.95	32.20 ± 1.36

Figure S17. Retention times (t_R), separation conditions, and HPLC analysis details

Compound	t_R ± SD (min)	Linear equation (μM)	Correlation coefficient (R^2)	Mobile phase (v/v)
GLIM	48.38 ± 0.30	y = 0.4663x + 0.5029	0.9999	40:60, acetonitrile–20 mM KH_2PO_4 (pH 3.0 at 30 °C)
GLIQ	24.86 ± 0.16	y = 0.4990x + 2.5136	0.9996	50:50, acetonitrile–20 mM KH_2PO_4 (pH 3.0 at 30 °C)
GLIC	8.51 ± 0.01	y = 0.2334x + 2.8665	0.9983	50:50, acetonitrile–20 mM KH_2PO_4 (pH 3.0 at 30 °C)
GLIB	13.49 ± 0.02	y = 0.4524x + 3.1147	0.9992	50:50, acetonitrile–20 mM KH_2PO_4 (pH 3.0 at 30 °C)
GLIP	4.90 ± 0.01	y = 0.2909x + 4.5368	0.9954	50:50, acetonitrile–20 mM KH_2PO_4 (pH 3.0 at 30 °C)
CARB	3.30 ± 0.01	y = 0.0354x + 0.4035	0.9987	50:50, acetonitrile–20 mM KH_2PO_4 (pH 3.0 at 30 °C)
CHLO	5.05 ± 0.01	y = 0.2239x + 4.8406	0.9955	50:50, acetonitrile–20 mM KH_2PO_4 (pH 3.0 at 30 °C)
TOLA	6.95 ± 0.08	y = 0.2133x + 1.1084	0.9996	50:50, acetonitrile–20 mM KH_2PO_4 (pH 3.0 at 30 °C)
TOLB	5.92 ± 0.06	y = 0.2216x + 2.7567	0.9976	50:50, acetonitrile–20 mM KH_2PO_4 (pH 3.0 at 30 °C)
MESU	5.17 ± 0.01	y = 0.2043x + 3.9920	0.9948	50:50, acetonitrile–20 mM KH_2PO_4 (pH 3.0 at 30 °C)

Figure S18. Chromatogram of DOXO analysis

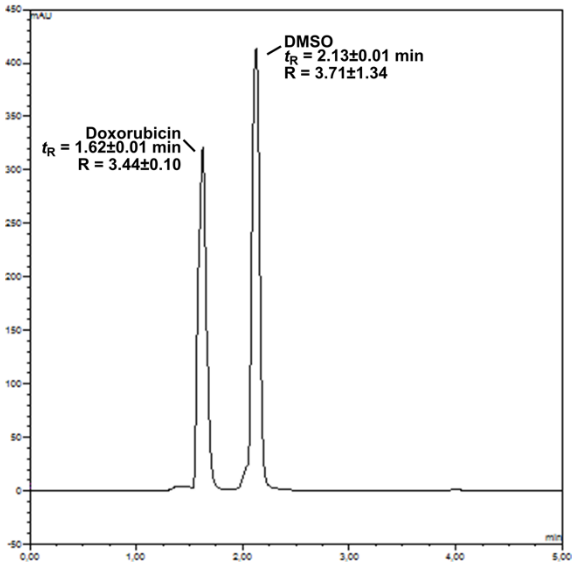

The analysis of doxorubicin decomposition in the presence of sulfonylureas was conducted on samples containing a DOXO+GLIM combination. An isocratic mobile phase, composed of a 40:60 (v/v) acetonitrile–buffer was employed. In this setup, doxorubicin (t_R = 1.62 ± 0.01 min, R = 3.44 ± 0.10) and DMSO (t_R = 2.13 ± 0.01 min, R = 3.71 ± 1.34) generated two well-separated peaks. While this HPLC system effectively separated the peaks of doxorubicin and DMSO, it resulted in prolonged retention times for the sulfonylureas. Consequently, in subsequent analyses, we utilized a mobile phase composed of a 50:50 (v/v) acetonitrile-buffer solution. Under these conditions, isocratic elution was unable to adequately separate doxorubicin and DMSO. However, the retention time of the sulfonylureas was significantly reduced.

Figure S19. Chromatogram of BSA analysis

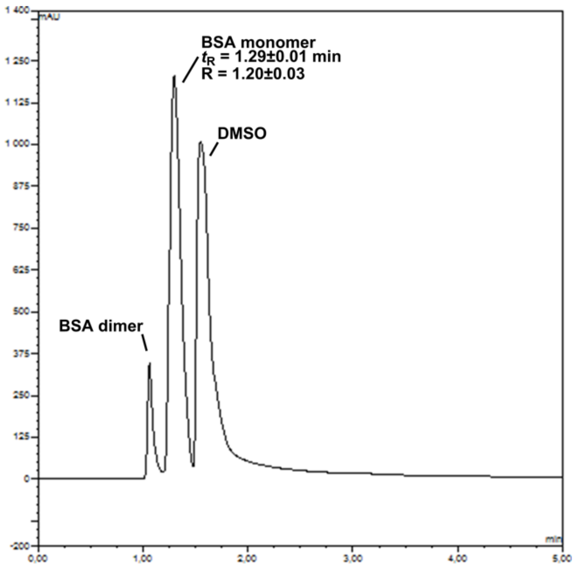

The determination of BSA levels in the samples was based on the monomeric BSA peak. An isocratic mobile phase, comprising a 50:50 (v/v) acetonitrile-buffer mixture, was used for this purpose. In each case, the BSA peak (t_R = 1.29 ± 0.01 min, R = 1.20 ± 0.03) provided sufficient chromatographic resolution to enable its quantification.

Figure S20. Comparative cytotoxicity of SUs from various studies

**Cell line**	**IC_{50} value (μM)**		
Drug	***GLIM***	***GLIC***	***GLIB***
**A375.S2**	198.6[10]	144.8[10]	305.3[10]
**A549**	285.8[10]; >200[26]; 352.9[a]	182.8[10]	436.4[10]
**HeLa**	184.4[10]	83.9[10]	177.4[10]
**HepG2**	176.6[a]	153.5[11]; 46.1[a]	410.6[a]
**K562**	89.4[10]	92.1[10]	85.3[10]
**MCF-7**	310.0[10]; 79.8[26]; 175.2[a]	216.3[10]; 87.9[a]	325.2[10]; >500[a]
**PANC-1**	409.0[10]	135.1[10]	495.0[10]
**T47D**	426.1[10]	81.9[10]	613.0[10]
**U-87MG**	186.2[a]	178.4[16]	

[a] – Our results.

[10] Alkhalil, M.; Al-Hiari, Y.; Kasabri, V.; Arabiyat, S.; Al-Zweiri, M.; Mamdooh, N.; Telfah, A. Selected pharmacotherapy agents as antiproliferative and anti-inflammatory compounds. *Drug Dev. Res.* **2020**, *81*, 470–490. https://doi.org/10.1002/ddr.21640.

[11] Subramaniyam, N.; Arumugam, S.; Ezthupurakkal, P.B.; Ariraman, S.; Biswas, I.; Muthuvel, S.K.; Balakrishnan, A.; Alsham-mari, G.M.; Chinnasamy, T. Unveiling anticancer potential of glibenclamide: Its synergistic cytotoxicity with doxorubicin on cancer cells. *J. Pharm. Biomed. Anal.* **2018**, *154*, 294–301. https://doi.org/10.1016/j.jpba.2018.03.025.

[16] Ru, Q.; Tian, X.; Wu, Y.-X.; Wu, R.-H.; Pi, M.-S.; Li, C.-Y. Voltage-gated and ATP-sensitive K+ channels are associated with cell proliferation and tumorigenesis of human glioma. *Oncol. Rep.* **2014**, *31*, 842–848. https://doi.org/10.3892/or.2013.2875.

[26] Faridi, U.; Al-Mutairi, F.; Parveen, H.; Khateeb, S. An in-vitro and in Silico Anticancer Study of FDA Approved Antidiabetic Drugs Glimepiride and Empagliflozin: Life Sciences-Biochemistry for better diagnosis and therapy. *Int. J. Life Sci. Biotechnol. Pharma Res.* **2022**, *10*, 52–57. https://doi.org/10.22376/ijpbs/lpr.2020.10.2.L52-57.
